# Nucleotide diversity patterns at the *DREB*1 transcriptional factor gene in the genome donor species of wheat (*Triticum aestivum* L)

**DOI:** 10.1371/journal.pone.0217081

**Published:** 2019-05-28

**Authors:** Yi Xu, Fang-Yao Sun, Chun Ji, Quan-Wen Hu, Cheng-Yu Wang, De-Xiang Wu, Genlou Sun

**Affiliations:** 1 College of Agronomy, Anhui Agricultural University, Hefei, Anhui, China; 2 Biology Department, Saint Mary’s University, Halifax, NS, Canada; Institute of Genetics and Developmental Biology Chinese Academy of Sciences, CHINA

## Abstract

Bread wheat (AABBDD) originated from the diploid progenitor *Triticum urartu* (AA), a relative of *Aegilops speltoides* (BB), and *Ae*. *tauschii* (DD). The *DREB*1 transcriptional factor plays key regulatory role in low-temperature tolerance. The modern breeding strategies resulted in serious decrease of the agricultural biodiversity, which led to a loss of elite genes underlying abiotic stress tolerance in crops. However, knowledge of this gene’s natural diversity is largely unknown in the genome donor species of wheat. We characterized the dehydration response element binding protein 1 (*DREB*1) gene-diversity pattern in *Ae*. *speltoides*, *Ae*. *tauschii*, *T*. *monococcum* and *T*. *urartu*. The highest nucleotide diversity value was detected in *Ae*. *speltoides*, followed by *Ae*. *tauschii* and *T*. *monococcum*. The lowest nucleotide diversity value was observed in *T*. *urartu*. Nucleotide diversity and haplotype data might suggest no reduction of nucleotide diversity during *T*. *monococcum* domestication. Alignment of the 68 *DREB*1 sequences found a large-size (70 bp) insertion/deletion in the accession PI486264 of *Ae*. *speltoides*, which was different from the copy of sequences from other accessions of *Ae*. *speltoides*, suggesting a likely existence of two different ancestral *Ae*. *speltoides* forms. Implication of sequences variation of *Ae*. *speltoides* on origination of B genome in wheat was discussed.

## Introduction

Frequent changes in climate, such as sudden low temperature, high temperature, and flooding, have caused serious damage to crop growth and development [1, 2], while, gradually, drought and salinity under increased agricultural pressure have constrained the yield and geographical distribution of global crops, resulting in a 70% reduction in their potential yield [3] and caused irreversible damage to field ecology [4]. Unreasonable practices will continue to increase the soil salinity [5]. The global drought problem may gradually increase in the foreseeable future [6]. Under the influence of plant growth and development [1], the mechanism of tolerance and adaptation of plant to abiotic stress has been a research hotspot.

In response to stress, plants respond with variety of complex processes of molecular regulation. When plant cells perceive external pressure, through signal transduction, plants turn on metabolic pathways to tolerate abiotic stress and synthesize a wide variety of stress-responsive proteins. These stress-responsive proteins include changes not only on the level of enzymes at the metabolic level, but also in composition at the transcriptional and translational levels.

The ethylene response element binding factor *(AP*2/*ERF*) family is a group of plant-specific transcriptional factors. Studies have shown that *AP*2/*ERF* transcriptional factors are involved in plant development and stress pathways [7]. Their main features contain at least an *AP*2 binding domain, a typical three-dimensional structure consisting of approximately 60 amino acid residues in the form of three β-sheets and one α-helix [8]. The *AP*2/*ERF* family is divided into 4 subfamilies based on their domains, namely the *AP*2 subfamily, the *RAV* subfamily, the *ERF* subfamily, and the dehydration response element binding protein (*DREB*) subfamily [9]. The *AP*2 subfamily contains two *AP*2 domains that are highly similar and repeat in tandem. The *AP*2/*ERF* transcriptional factor containing one *AP*2 domain and one *B*3 domain was named as the *RAV* subfamily. Both the *DREB* subfamily and the *ERF* (ethylene response transcription factor) subfamily contain only one *AP*2 domain, but their main differences are the fourteenth and nineteenth amino acid residues of the *AP*2 domain. The fourteenth amino acid in the *DREB* subfamily is proline, the nineteenth is glutamic acid, the fourteenth amino acid in the ERF subfamily is alanine, and the nineteenth is aspartic acid [9].

The *DREB* subfamily includes two subgroups, *DREB*1 and *DREB*2 [10]. *DREB* transcriptional factor specifically binds to the *DRE*/*CRT* cis-element in the promoter and can activate the expression of many stress-inducible genes, thereby enhancing plant tolerance to stress. *Arabidopsis DREB1A* and *DREB*2*A* transcriptional factor genes were induced by low temperature or drought and high salinity, and *DREB*1*A* and *DREB*2 transcription factors were generated to regulate *rd*17, *kin*1, *cor*6.6, *cor*15a, *erd*10, which are related to low temperature and high salt stress tolerance, respectively [10]. *HvDREB*1 was isolated from barley and found to bind as a transcriptional factor to *DRE/CRT* elements. Overexpression of *HvDREB*1 increased *Arabidopsis* resistance to salt stress [11]. Under drought and high temperature stress, *DREB*2s binds to *DRE/CRT* elements as transcriptional factors in *Arabidopsis* to induce downstream gene expression and increase plant resistance to abiotic stresses [12].

Hexaploid *Triticum aestivum* L. (AABBDD) (2n = 6x = 42) is derived from the three homologous genomes, A, B, and D, with an approximate genome size of 16–17 Gb [13–15]. The progenitor of the A genome contains *Triticum urartu* Thum ex Gand (genome A^u^) [16] and *Triticum monococcum* Linn (genome A^m^). *T*. *urartu* has been considered as the A-genome donor to tetraploid and hexaploid wheat species [17, 18]. The origin of the B genome is still under debate, in spite of a large number of attempts to identify the parental species [17], but *Ae*. *speltoides* (S genome) has been suggested as the most likely progenitor of the B genome [19, 20]. *Ae*. *tauschii* Coss (genome DD) has been documented as the D genome progenitor of *T*. *aestivum* [17].

The modern breeding strategies resulted in serious decrease of agricultural biodiversity, which led to a loss of elite genes underlying abiotic stress tolerance in crop [21]; therefore, exploitation of the genetic resources of wild relatives is a widely used strategy to increase biodiversity for crop improvement. In recent years, new introgression lines between commercial cultivars and wild relatives have been generated in many crops. As an example, the genes from wheat wild relatives *Aegilops umbellulata* and *Elytrigia elongata* [22–24] have been introgressed into wheat to significantly improve abiotic stress tolerance traits in wheat.

DREB*1*genes in wheat were located on chromosomes 3A, 3B and 3D [25], and can be induced by low temperature, abscisic acid (ABA), salinity and drought [26]. The nucleotide diversity π and *θ* values of wheat *DREB* gene on 1A chromosome have been characterized [27]. However, the nucleotide diversity of *DREB1*gene in wheat genome donor species is uncharacterized. In order to efficiently use of wild relatives for improving wheat tolerance to abiotic stress, we analyzed the nucleotide diversity of *DREB*1 gene among the genomes of *Ae*. *speltoides*, *Ae*. *tauschii*, *T*. *monococcum*, and *T*. *urartu*. The haplotype diversity and evolutionary factors combined with the relationship between *DREB1* transcriptional factors and stress resistance further explore the evolution and origin of the wheat-tribe genome.

## Materials and methods

### Plant materials

Thirteen accessions of *Aegilops speltoides* (S genome), 12 accessions of *Ae*. *tauschii* (D genome), 24 accession of *Triticum monococcum* (A^m^ genome) and 19 accessions of *T*. *urartu* (A^u^ genome) were sampled (Table 1). The seeds were provided by USDA (United States Department of Agriculture). Germinated seeds were transplanted to a sand-peat mixture, and the plants maintained in a greenhouse. Twenty-three sequences from other Triticeae species and *Brachypodium distachyon* downloaded from NCBI website were included in phylogenetic analysis.

**Table 1:** The accessions of *Aegilops speltoides*, *Ae*. *tauschii*, *Triticum monococcum* and *T*. *urartu* used in this study.

Species	Accession No.	Genome	Origin
*Ae*. *speltoides*	PI 170204	S	Kirklareli, Turkey
*Ae*. *speltoides*	PI 219867	S	Arbil, Iraq
*Ae*. *speltoides*	PI 369602	S	Unknown
*Ae*. *speltoides*	PI 369608	S	Unknown
*Ae*. *speltoides*	PI 393493	S	Israel
*Ae*. *speltoides*	PI 487235	S	Al Ladhjiqiyah. Syria
*Ae*. *speltoides*	PI 487236	S	Al Ladhjiqiyah. Syria
*Ae*. *speltoides*	PI 487237	S	Al Ladhjiqiyah. Syria
*Ae*. *speltoides*	PI 486263	S	Diyarbakir, Turkey
*Ae*. *speltoides*	PI 486264	S	Diyarbakir, Turkey
*Ae*. *speltoides*	PI 554297	S	Diyarbakir, Turkey
*Ae*. *speltoides*	PI 573450	S	Ankara, Turkey
*Ae*. *speltoides*	PI 573452	S	Ankara, Turkey
*Ae*. *tauschii*	PI 220642	D	Faryab, Afghanistan
*Ae*. *tauschii*	PI 317392	D	Badghis, Afghanistan
*Ae*. *tauschii*	PI 452130	D	Henan, China
*Ae*. *tauschii*	PI 452131	D	Qinghai, China
*Ae*. *tauschii*	PI 486275	D	Kars, Turkey
*Ae*. *tauschii*	PI 508260	D	Xinjiang, China
*Ae*. *tauschii*	PI 511379	D	West Azerbaijan, Iran
*Ae*. *tauschii*	PI 554320	D	Hakkari, Turkey
*Ae*. *tauschii*	PI 554323	D	Van, Turkey
*Ae*. *tauschii*	PI 554324	D	Kars, Turkey
*Ae*. *tauschii*	PI 603230	D	Azerbaijan
*Ae*. *tauschii*	PI 603255	D	Erevan, Armenia
*T*. *monococcum*	Cltr14520	A^m^	Canada
*T*. *monococcum*	PI 10474	A^m^	Thuringia, Germany
*T*. *monococcum*	PI 94740	A^m^	Spain
*T*. *monococcum*	PI 168804	A^m^	Kansas, USA
*T*. *monococcum*	PI 190915	A^m^	Belgium
*T*. *monococcum*	PI 190946	A^m^	Portugal
*T*. *monococcum*	PI 191383	A^m^	Ethiopia
*T*. *monococcum*	PI 225164	A^m^	Greece
*T*. *monococcum*	PI 237659	A^m^	Rift Valley, Kenya
*T*. *monococcum*	PI 265008	A^m^	Bosnia
*T*. *monococcum*	PI 272561	A^m^	Hungary
*T*. *monococcum*	PI 277130	A^m^	Permet, Albania
*T*. *monococcum*	PI 286068	A^m^	Poland
*T*. *monococcum*	PI 306543	A^m^	Romania
*T*. *monococcum*	PI 307984	A^m^	Morocco
*T*. *monococcum*	PI 326317	A^m^	Azerbaijan
*T*. *monococcum*	PI 343181	A^m^	Santiago, Chile
*T*. *monococcum*	PI 352486	A^m^	Switzerland
*T*. *monococcum*	PI 355519	A^m^	Iran
*T*. *monococcum*	PI 362610	A^m^	Macedonia
*T*. *monococcum*	PI 393496	A^m^	Israel
*T*. *monococcum*	PI 418582	A^m^	Azerbaijan
*T*. *monococcum*	PI 427927	A^m^	Iraq
*T*. *monococcum*	PI 560720	A^m^	Turkey
*T*. *urartu*	Cltr17668	A^u^	Armenia
*T*. *urartu*	PI 428180	A^u^	Armenia
*T*. *urartu*	PI 428183	A^u^	Armenia
*T*. *urartu*	PI 428208	A^u^	Mardin, Turkey
*T*. *urartu*	PI 428215	A^u^	Mardin, Turkey
*T*. *urartu*	PI 428231	A^u^	Urfa, Turkey
*T*. *urartu*	PI 428237	A^u^	Urfa, Turkey
*T*. *urartu*	PI 428241	A^u^	Urfa, Turkey
*T*. *urartu*	PI 428287	A^u^	El Beqaa, Lebanon
*T*. *urartu*	PI 428323	A^u^	El Beqaa, Lebanon
*T*. *urartu*	PI 487267	A^u^	Al Hasakah, Syria
*T*. *urartu*	PI 538726	A^u^	Mardin, Turkey
*T*. *urartu*	PI 538727	A^u^	Mardin, Turkey
*T*. *urartu*	PI 538728	A^u^	Urfa, Turkey
*T*. *urartu*	PI 662238	A^u^	Iran
*T*. *urartu*	PI 662239	A^u^	Iran
*T*. *urartu*	PI 662241	A^u^	Iran
*T*. *urartu*	PI 662242	A^u^	Iran
*T*. *urartu*	PI 662264	A^u^	Jordan

### DNA extraction, amplification and sequencing

Leaf tissue samples were collected and frozen in liquid nitrogen. DNA was isolated using the GeneJet Plant Genomic DNA Purification Mini Kit according to the manufacture’s instruction (Thermo Scientific). The isolated genomic DNA was stored at -20°C for use.

The primers for amplifying the *DREB1* sequence were designed based on *Triticum aestivum AP*2-containing protein (*DREB1*) mRNA (AF303376.1). The forward primer sequence was 5’-GAAGAAAGTGCGCAGGAGAAG-3’ (Dreb1F), reversed primer was 5’- TCCCTATTGCTCCGCATGAC-3’(Dreb1R). The sequence was amplified in a 15 μl reaction containing 20 ng template DNA, 0.25 mM dNTP, 2.0 mM MgCL_2_, 0.25 μM of each primer and 2.0 U *Taq* polymerase (TransGen, Beijing, China). The amplification profile was as follows: an initial denaturation at 95°C for 5 min and 36cycles of 95°C for 45 sec, 58°C for 50 sec, 72°C for 150 sec. The cycling ended with 72°C for 10 min. PCR products were purified using the EasyPure Quick Gel Extraction Kit (TransGen, Beijing, China) according to manufacturer’s instruction.

PCR products were commercially sequenced by the Shanghai Sangon Biological Engineering & Technology Service Ltd (Shanghai, China). To enhance the sequence quality, both forward and reverse strands were sequenced independently. To avoid any error which would be induced by *Taq* DNA polymerase during PCR amplification, each sample was independently amplified twice and sequenced.

### Data analysis

Automated sequence outputs were visually inspected with chromatographs. Multiple sequence alignments were performed using ClustalX with default parameters. Maximum-parsimony (MP) method was used to perform phylogenetic analysis using computer program PAUP* ver. 4 beta 10 [28]. All characters were specified as unweighted and unordered. The most parsimonious trees were constructed by performing a heuristic search using the Tree Bisection-Reconnection (TBR) with the following parameters: MulTrees on and ten replications of random addition sequences with the stepwise addition option. A strict consensus tree was generated from multiple parsimonious trees. The consistency index (CI) and the retention index (RI) were used to estimate the overall character congruence. Bootstrap (BS) values with 1,000 replications [29] that was calculated by performing a heuristic search using the TBR option with Multree on were used to test the robustness of clades.

Bayesian analysis was used to for phylogeny analysis of haplotypes. The jModelTest 2.1.10 [30] was used to calculate the best-fitting model of sequence evolution using default parameters. The Maximum likelihood value (-LnL), Akaike information criterion (AIC) [31] and Bayesian Information Criterion (BIC) [32] were estimated. The model test showed the TrN+I substitution model led to best BIC and AIC scores, therefore, the TrN+I model was used in the Bayesian analysis using MrBayes 3.1 [33]. MrBayes 3.1 was run with the program’s standard setting of two analyses in parallel, each with four chains, and estimates of convergence of results were determined by calculating standard deviation of split frequencies between analyses. 689,000 generations were run to make the standard deviation of split frequencies < 0.01. Samples were taken every 1000 generations. The first 25% of samples from each run were discarded as burn-in to ensure the stationary of the chains. Bayesian posterior probability (PP) values were used to test the robustness of clades.

Nucleotide diversity values of Tajima’s π [34] and Watterson’s θ [35] as well as tests of neutral evolution [36] were performed using the software program DnaSP 4.0 [37].

The analysis of protein domain and conserved motif of sequences was characterized using Pfam (https://pfam.xfam.org/search) and Multiple Em for Motif Elicitation (MEME) software [38].

## Results

### Sequence analysis

**Fig 1:**
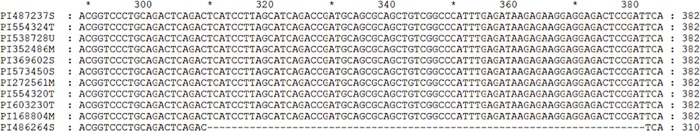
Partial alignment of the amplified sequences of *DREB1*. Note that a large size (70 bp) insertion/deletion in the sequence from the accession PI486264 of *Ae*. *speltoides*.

The DNA from 13 accessions of *Aegilops speltoides* (S genome), 12 accessions of *Ae*. *tauschii* (D genome); 24 accession of *Triticum monococcum* (A^m^ genome); and 19 accessions of *T*. *urartu* (A^u^ genome) were amplified using the Dreb1F/R primer pair. The size of amplified products from these DNA was approximately 850 bp. Complete alignment of the 68 *DREB*1 sequences detected a large-size (70 bp) insertion/deletion in the accession PI486264 of *Ae*. *speltoides* (Fig 1). BLAST search against NCBI found that this sequence shared 100% identity with *T*. *aestivum DREB* transcription factor 6 (*DREB*6) mRNA (AY781361.1); the sequence on *T*. *aestivum* chromosome 3B (LS992087); and *T*. *aestivum* genome B dehydration-responsive element-binding protein (*DREB*1) gene, partial cds (DQ195069.1). BLASTX search found that protein sequence of this accession matched with *DREB* transcription factor 6 (AAX13289.1) of *T*. *aestivum* with 100% identity, while it lost “KDESESPPSLISNAPTAALHRSDA” when compared with AP2-containing protein in *T*. *aestivum* (AAL01124.1).

The haplotypes of *DREB*1 sequences from *Ae*. *speltoides*, *Ae*. *tauschii*, *T*. *monococcum*, and *T*. *urartu* were calculated. A total of 19 haplotypes were identified in the 68 accessions of these four species. Seven, 7, 10, and 4 haplotypes were detected from 13 sequences of *Ae*. *speltoides*, 12 sequences of *Ae*. *tauschii*, 24 sequences of *T*. *monococcum*, and 19 sequences of *T*. *urartu*, respectively (Table 2). Twenty-six out of 68 accessions belonged to the Hap 2, and 15 belonged to the Hap 7. For each species, most *Ae*. *speltoides* sequences belonged to Hap 2 with frequency of 0.538. The Hap 2, Hap 7, and Hap 2 showed the highest frequency in *Ae*. *tauschii*, *T*. *monococcum*, and *T*. *urartu*, respectively. Thirteen haplotypes were species-specific, while two haplotypes (Hap 1 and Hap 7) were shared by *Ae*. *speltoides*, *Ae*. *tauschii*, and *T*. *monococcum*; Hap 8 and Hap 10 was shared by *Ae*. *speltoides* and *T*. *monococcum*, and by *Ae*. *tauschii* and *T*. *monococcum*, respectively. Only one haplotype (Hap 2) was commonly detected among four species (Table 2).

**Table 2:** Haplotype frequencies of Dreb1 gene in *Ae*. *speltoides*, *Ae tauschii*, *T*. *monococcum* and *T*. *urartu* populations.

Dreb1	*Ae*. *speltoides*	*Ae tauschii*	*T*. *monococcum*	*T*. *urartu*	Overall
**Hap1**	0.077 (1)	0.083 (1)	0.042 (1)	0	0.044 (3)
**Hap2**	0.538 (7)	0.333 (4)	0.083 (2)	0.684 (13)	0.382 (26)
**Hap3**	0	0.167 (2)	0	0	0.029 (2)
**Hap4**	0	0	0	0.158 (3)	0.044 (3)
**Hap5**	0	0	0	0.053 (1)	0.015 (1)
**Hap6**	0	0	0.125 (3)	0	0.044 (3)
**Hap7**	0.077 (1)	0.167 (2)	0.500 (12)	0	0.221 (15)
**Hap8**	0.077 (1)	0	0.042 (1)	0	0.029 (2)
**Hap9**	0	0	0.042 (1)	0	0.015 (1)
**Hap10**	0	0.083 (1)	0.042 (1)	0	0.029 (2)
**Hap11**	0	0	0	0.105 (2)	0.029 (2)
**Hap12**	0	0	0.042 (1)	0	0.015 (1)
**Hap13**	0.077 (1)	0	0	0	0.015 (1)
**Hap14**	0.077 (1)	0	0	0	0.015 (1)
**Hap15**	0	0	0.042 (1)	0	0.015 (1)
**Hap16**	0	0.083 (1)	0	0	0.015 (1)
**Hap17**	0	0.083 (1)	0	0	0.015 (1)
**Hap18**	0	0	0.042 (1)	0	0.015 (1)
**Hap19**	0.077 (1)	0	0	0	0.015 (1)

### Nucleotide diversity

Tajima’s *π* and Watterson’s *θ* were used to determine nucleotide diversity for each species studied here (Table 3). The highest nucleotide diversity values (*π* and *θ*) were detected in *Ae*. *speltoides* with *π* = 0.00456 and *θ* = 0.00685, followed by *Ae*. *tauschii* (*π* = 0.00320 and *θ* = 0.00281), and *T*. *monococcum* (*π* = 0.00301 and *θ* = 0.00325). The lowest nucleotide diversity values (*π* and *θ*) were observed in *T*. *urartu* with *π* = 0.00146 and *θ* = 0.00173. Tajima and Fu and Li’s D statistics were also calculated for each species. Tajima’s D values for *Ae*. *speltoides*, *Ae*. *tauschii*, *T*. *monococcum*, and *T*. *urartu* were -1.40161, 0.53752, -0.23968 and -0.49107, respectively, which were all not significant. Fu and Li’s D values and Fu and Li’s F test for these four species were also not significant (Table 3).

**Table 3:** Estimates of nucleotide diversity per base pair and test statistics for *Dreb1*gene in *Aegilops speltoides*, *Ae*. *tauschii*, *Triticum monococcum* and *T*. *urartu* populations.

Population	No. of accessions	No. of haplotypes (H)	Haplotype diversity (Hd)	Theta (per site) from S(θ)	Nucleotide diversity (π)	Tajima's D test	Fu and Li's D test	Fu and Li's F test
All	68	19	0.806	0.00555±0.00186	0.00364±0.00049	-1.04475	-2.33023	-2.22299
*Aegilops speltoides*	13	7	0.731	0.00685±0.00171	0.00456±0.00208	-1.40161	-1.74285	-1.88685
*Aegilops tauschii*	12	7	0.879	0.00281±0.00145	0.00320±0.00058	0.53752	0.28246	0.39461
*Triticum monococcum*	24	10	0.746	0.00325±0.00143	0.00301±0.00063	-0.23968	-0.10361	-0.16762
*Triticum urartu*	19	4	0.520	0.00173±0.00094	0.00146±0.00040	-0.49107	0.40275	0.17861

### Phylogenetic analysis

**Fig 2:**
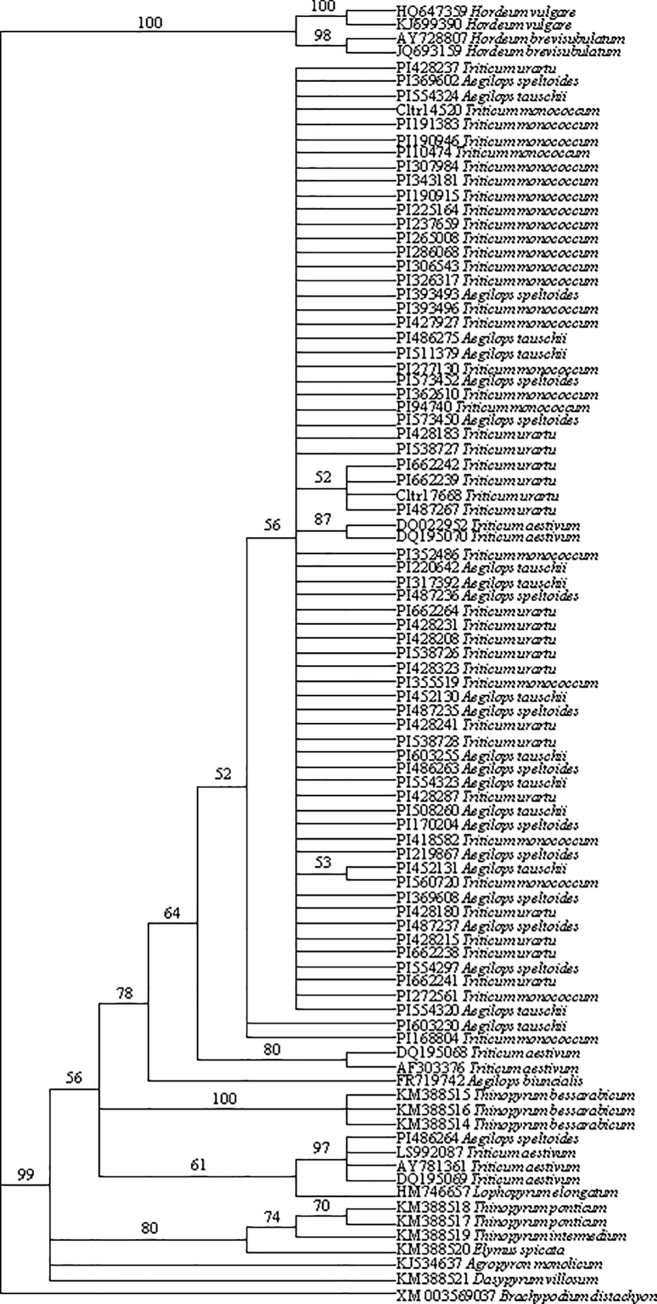
The consensus trees derived from *DREB*1 sequence data was conducted using heuristic search with TBR branch swapping. Numbers above are bootstrap values from maximum parsimony. *Brachypodium distachyon* was used as an outgroup. Consistency index (CI) = 0.864, retention index (RI) = 0.935.

The phylogenetic relationship of 90 *DREB*1 sequences from *Ae*. *speltoides*, *Ae*. *tauschii*, *T*. *monococcum*, and *T*. *urartu* along with *DREB*1 sequences from other Triticeae species was analyzed using the maximum parsimony. The sequence from *Brachypodium distachyon* was used as an outgroup. The maximum parsimony analysis resulted in 236 most parsimonious trees (642 constant characters, 110 parsimony-uninformative characters, 77 parsimony-informative characters, CI excluding uninformative characters = 0.864; RI = 0.935). The strict consensus phylogenetic tree yielded obvious *Aegilops* + *Triticum* and *Hordeum* species group (Fig 2) with highly supported bootstrap values (78% and 100%, respectively). All sequences from *Aegilops* and *Triticum* species studied here were grouped into the *Aegilops* + *Triticum* except the sequences of *T*. *aestivum* from B genome and *Ae*. *speltoides* accession PI 486264, which formed a group with 97% bootstrap support. Within the *Aegilops* + *Triticum* clade, the sequence DQ195070 encoding dehydration responsive element binding protein (*DREB1*) on A genome of *T*. *aestivum* was grouped with the DQ022952, dehydration responsive element binding protein W73 mRNA (87% bootstrap support), and was nested within the most sequences from *Ae*. *speltoides*, *Ae*. *tauschii*, *T*. *monococcum*, and *T*. *urartu* (56% bootstrap support). The sequence DQ195068 encoding dehydration-responsive element binding protein on D genome of *T*. *aestivum* was grouped with the sequence AF303376, *AP*2-containing protein (*DREB*1) mRNA (80% bootstrap value), and was sister to the largest group containing sequences from *Ae*. *speltoides*, *Ae*. *tauschii*, *T*. *monococcum*, *T*. *urartu*, and A genome of *T*. *aestivum*.

**Fig 3:**
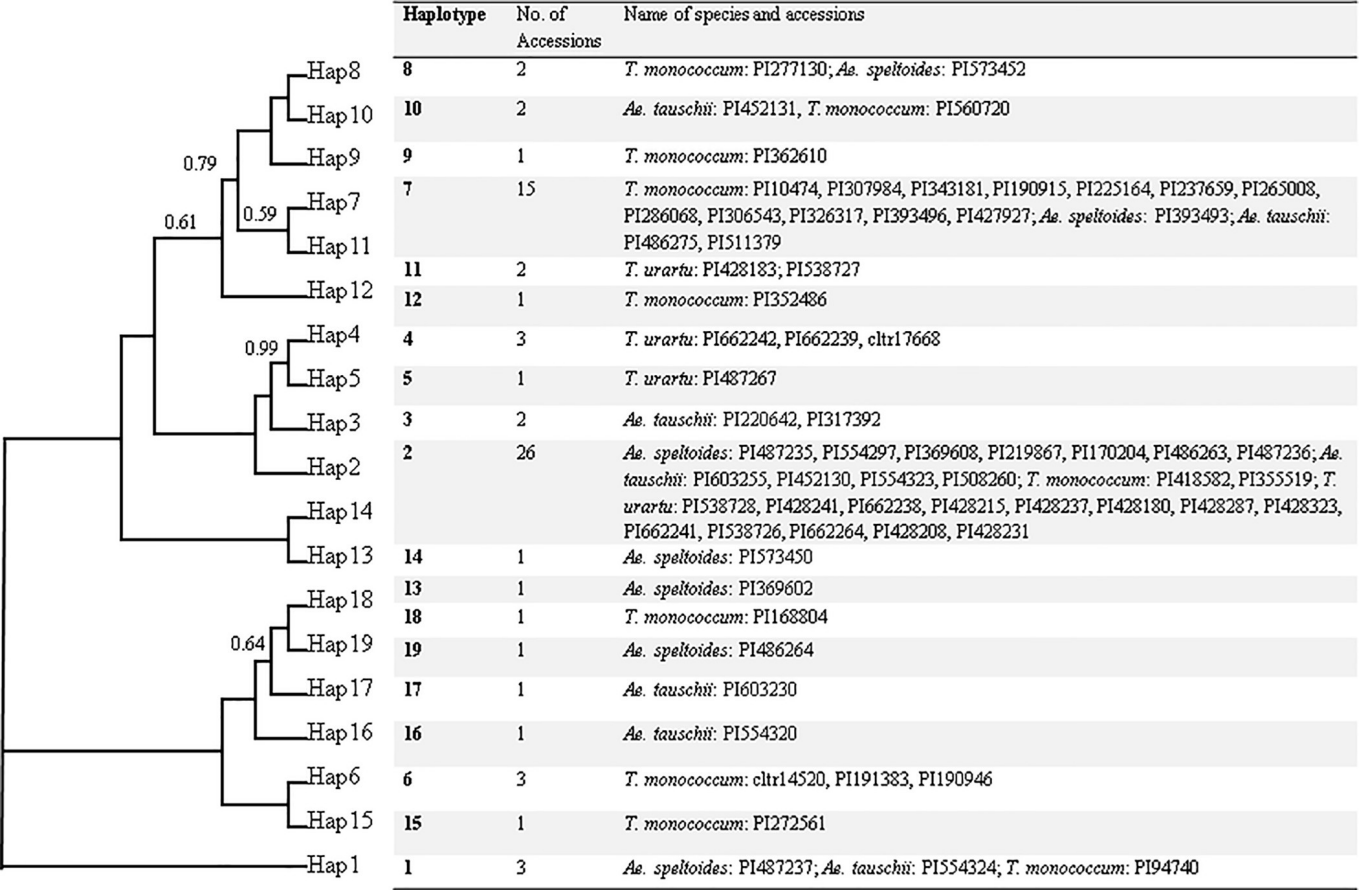
The relationship of the 19 haplotypes from accessions of *Ae*. *speltoides*, *Ae*. *tauschii*, *T*. *monococcum*, *T*. *urartu* was revealed by Bayesian analysis. The value above branch was Bayesian posterior probability (PP) values. The full list of haplotype was placed aside the tree.

The relationship of the 19 haplotypes from accessions of *Ae*. *speltoides*, *Ae*. *tauschii*, *T*. *monococcum*, and *T*. *urartu* was revealed by Bayesian analysis, and shown in Fig 3 with Bayesian posterior probability (PP) values above branch. Haplotypes of *Ae*. *speltoides*, *Ae*. *tauschii*, *T*. *monococcum*, and *T*. *urartu* were grouped into different clades (Fig 3). The Hap 4 and Hap 5 showed a close relationship with a well-supported value (PP = 0.99).

**Fig 4:**
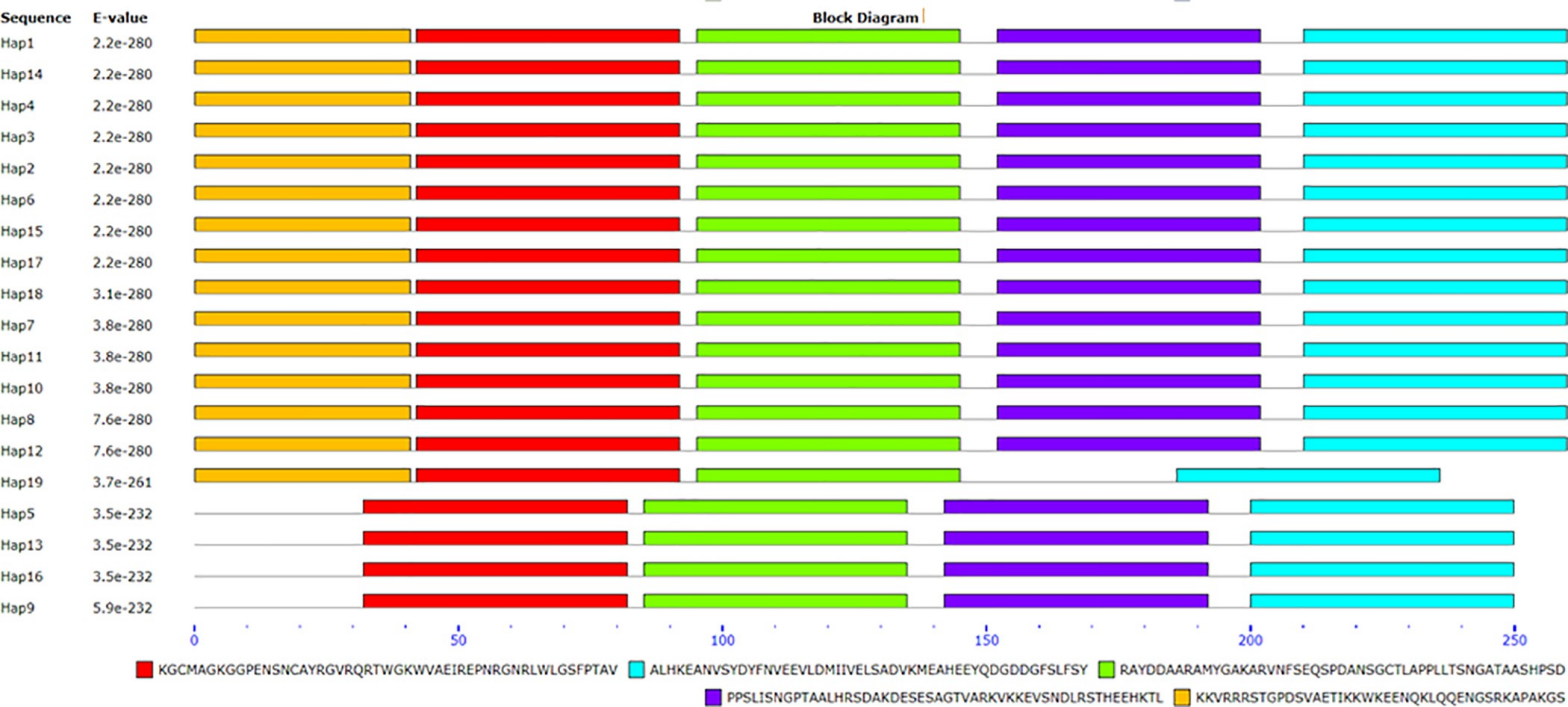
The conserved motif analysis of *DREB* proteins in the 19 haplotypes from accessions of *Ae*. *speltoides*, *Ae*. *tauschii*, *T*. *monococcum*, *T*. *urartu* using MEME server. Each motif was represented in boxes with different colors.

Pfam analysis showed that all sequences contain *AP*2 domain structure. The conserved motif analysis of the *DREB* proteins found that the haplotype 19 of *DREB* sequence did not have motif “PPSLISNGPTAALHRSDAKDESESAGTVARK VKKEVSNDLRSTHEEHKTL”, the haplotype 5, 9, 13, and 16 did not have motif “KKVRRRSTGPDSVAETIKKWKEENQKLQQENGSRKAPAKGS” (Fig 4).

## Discussion

### Nucleotide diversity in *Ae*. *speltoides*, *Ae*. *tauschii*, *T*. *monococcum*, and *T*. *urartu*

Previous studies have provided evidence that crop domestication and modern breeding strategies resulted in serious reduction of genetic diversity on various species [39, 40], which led to a loss of elite genes underlying abiotic stress tolerance in crop [21]; therefore, exploitation of the genetic resources of wild relatives is widely used strategy to increase biodiversity for crop improvement. Characterization of genetic diversity not only has significant effect on genetic improvement and resistance research, but also provides new direction for the conservation and utilization of genetic resources in germplasm gene banks [41]. The nucleotide diversity of *Ae*. *speltoides*, *Ae*. *tauschii*, *T*. *monococcum*, and *T*. *urartu* was examined here.

The genomes of *T*. *urartu* Thum ex Gand (genome A^u^) and *T*. *monococcum* Linn (genome A^m^) have similar genome size and gene content [42]. *Triticum urartu*, the wild diploid wheat from the Fertile Crescent region, has long been considered as the A-genome donor to tetraploid and hexaploid wheat species [17, 43]. The diploid wheat *T*. *monococcum* was among the first domesticated crops in the Fertile Crescent 10,000 years ago [44]. Our results showed that both the number of haplotypes and nucleotide diversity values of *T*. *monococcum* were much higher than those of *T*. *urartu*, which might suggest “no reduction of nucleotide diversity during *T*. *monococcum* domestication” made from a study of 18 loci in 321 wild and 92 domesticated lines of *Triticum* species [44], but was not consistent with the Qi et al. [45]. That higher variability of *DREB*1 in *T*. *monococcum* than in *T*. *urartu* could be due to *DREB*1 regulating role in response to abiotic stress, as this kind of gene might be experienced elevated rates of mutations and adaptation. This was evidenced by the highest number of haplotypes detected in *T*. *monococcum* among the four species analyzed here. Indirectly, this variation generated in *T*. *monococcum* might be a source to wheat breeding to improve the resistance to abiotic stress.

Higher nucleotide diversity value of *DREB*1 in *Ae*. *speltoides* than that in *Ae*. *tauschii*; *T*. *monococcum*; and *T*. *urartu* was expected, and might agree well with previous studies [45, 46]. This might attribute to the mating system of these species. *Aegilops speltoides* is an outcrossing species, while *Ae*. *tauschii*, *T*. *monococcum*, and *T*. *urartu* are inbreeding species. Mating system is one of the major factors controlling molecular diversity [44, 47, 48]. It was reported that averaged π value (0.01323) of *Acc*-1 gene in the genomes of outcrossing species was two-fold of the value (0.005664) in the genomes of selfer in Triticeae species [49].

In general, genetic bottlenecks acting on neutrally evolving loci during either the domestication process or subsequent breeding, or both, are sufficient to account for reduced diversity [50]. In domesticated forms, this reduction is evident in a shift toward more positive values of Tajima’s D in the domesticated relative to wild species population [51, 52]. Domesticated *T*. *monococcum* showed negative Tajima’s D values (Table 3), suggesting that there might be no genetic bottlenecks effects on or a signature of a recent population expansion of *T*. *monococcum*.

### Comparison of nucleotide diversity of *DREB*1 gene with other species

Since *DREB*1*/CBF* (dehydration responsive element binding/C-repeat binding factor) encoding genes in abiotic stress have important roles in responding to abiotic stress, nucleotide diversity of *DREB*1 gene has been characterized in several plants [53–57]. In 126 wheat lines, the nucleotide diversity π and *θ* values of wheat *DREB* gene on 1A chromosome were 0.180 and 0.392, respectively [27], which were much higher than the values detected in A genome *T*. *monococcum* and *T*. *urartu*. *DREB*1*A* nucleotide diversity was calculated from the 126 wheat lines that were developed by the International Maize and Wheat Improvement Center from entries in the elite spring wheat yield trial, semiarid wheat yield trial, and high temperature wheat yield trial [57]. These lines, during the breeding procedure, might suffer different natural selection pressure, resulting in the wide range of diversity of this gene. Speculatively, the significant Tajima’s D value of *DREB*1*A* in these wheat lines might be an indicator of presence of selection footprints, while the Tajima’s D value of *DREB*1 in *T*. *monococcum* and *T*. *urartu* accessions studied here did not reach significant level.

The haplotype (gene) diversity of *DREB*1 gene among the 10 promising upland and lowland cultivars rice was 0.756 [53], which is comparable to the haplotype diversity detected in *Ae*. *speltoides* and *T*. *monococcum*, but higher than that in *T*. *urartu*. The nucleotide diversity (π) of *DREB*1 in 191 chickpea was 0.0011 [54], which is similar to the values detected in this study, while the nucleotide diversity in *C*. *canephora* CDS region was π = 0.0101, θ = 0.0080) [56], which was much higher than that in our study. This might be attributed to the nature of species.

### Conserved motif of *DREB* proteins

The conserved motif analysis of the *DREB* proteins found that some sequences did not have “PPSLISNGPTAALHRSDAKDESESAGTVARKVKKEVSNDLRST HEEHKTL”, and motif “KKVRRRSTGPDSVAETIKKWKEENQKLQQENGS RKAPAKGS”. All sequences contain *AP*2 domain structure, suggesting the structural diversity and functional similarity of the *DREB* gene in these species. Allele mining across *DREB*1*A* and *DREB*1*B* in diverse rice genotypes also found indels across *DREB*1*A* and *DREB*1*B* [55]. Since *DREBs* are important transcriptional factors regulating stress-responsive gene expression, the highly conserved domains in these genes are essential for their specific biological functions. Further correlated the SNPs and indels in the *DREB*1 with its genotype responding to stress will enhance our understanding the role played by this gene.

### Implication of sequences variation of *Ae*. *speltoides* on origination of B genome in wheat

Overwhelming evidences have suggested that the diploid ancestor of the B genome of tetraploid and hexaploid wheat species is closely related to the S genome of *Aegilops speltoides* in the *Sitopsis* section (SS, 2n = 14) [19, 42, 43, 58–60]. However, none of the presently known species in this group have all properties of the B-genome [60]. A study on transposable elements (TEs) suggested that the S genome of *Ae*. *speltoides* has diverged very early from the progenitor of the B genome which remains to be identified [58]. Analysis of the *Pgk-1* gene among the *Ae*. *speltoides* accessions revealed an 89 bp indel in the intron of the *Pgk-1* gene, indicating that likely existence of two different ancestral *Ae*. *speltoides* forms, which gave rise to two evolutionarily close lineages of polyploid wheats [61]. The *Wcor15* results suggested that *Ae*. *speltoides* might be the direct donor of the *Wcor15-2B* in tetraploid and hexaploid wheat varieties [42]. Our study here also revealed two forms of *DREB*1 sequences in *Ae*. *speltoides*, suggesting “likely existence of two different ancestral *Ae*. *speltoides* forms” [61]. The form in the accession PI486264 shared 100% identity with the sequences on *T*. *aestivum* chromosome 3B, which might be more likely the B donor genome of wheat.

Recent studies suggested that mono-or polyphyletic B subgenome origin cannot explain entirely the observed accumulation of mutations during evolution in shaping the modern bread wheat B subgenome. The consequences of a differential evolutionary plasticity of the B subgenome was proposed as an alternative scenario where the increased divergence of the B subgenome in the hexaploid wheat compared to *Ae*. *speltoides* at the sequences level [62]. Phylogenetic analysis routinely applied to test evolutionary questions and to trace the origin of polyploidy is based on assumptions that intraspecifc variation is smaller than interspecific variation, and that within and between species, sample sizes are sufficiently large enough to capture variation at both levels [63]. When sampling a single individual per species or treating each individual or haplotype as a separate terminal taxon could delineate the potential risk of bias [64]. Intraspecific variation is abundant in all types of systematic characters which could cause bias in the phylogenetic analyses [65], such as in *Ae*. *speltoides*. Our results suggested that, in order to reveal the origination of B subgenome in the modern bread, it is critical to include wide range of accessions of *Ae*. *speltoides* in phylogenetic analysis.

In summary, the highest *DREB*1 gene diversity was detected in *Ae*. *speltoides*, followed by *Ae*. *tauschii* and *T*. *monococcum*. The lowest nucleotide diversity value was observed in *T*. *urartu*. Both the number of haplotypes and nucleotide diversity values of *T*. *monococcum* were much higher than those of *T*. *urartu*, which likely supports no reduction of nucleotide diversity during *T*. *monococcum* domestication [44]. Our study here revealed two forms of *DREB*1 sequences in *Ae*. *speltoides*. The form in the accession PI486264 shared 100% identity with the sequences on *T*. *aestivum* chromosome 3B, which might be more likely the B donor genome of wheat. Our results suggested that, in order to reveal the origination of B subgenome in the modern bread, it is critical to include wide range of accessions of *Ae*. *speltoides* in phylogenetic analysis. Stress tolerance study such as drought on these materials will be conducted to make possibly link of the haplotype with gene expression in future.

## Supporting information

S1 Table Sequences used in phylogenetic analysis. (DOCX)
