# Supplementary material for: Nucleotide diversity patterns at the DREB1 transcriptional factor gene in the genome donor species of wheat (Triticum aestivum L)
Source: PLoS One. 2019 May 28;14(5):e0217081. doi: 10.1371/journal.pone.0217081 (PMC6538315; doi:10.1371/journal.pone.0217081)
Supplement: S1 Table — (DOCX) [file pone.0217081.s001.docx]

S1 Table: Sequences used in phylogenetic analysis

HQ647359 GAAGAAAGTGCGCAGGAGAACCACTGGTCCAGATTCGGTTGCTGAAACTA

AK376344 GAAGAAAGTGCGCAGGAGAACCACTGGTCCAGATTCGGTTGCTGAAACTA

DQ012941 GAAGAAAGTGCGCAGGAGAACCACTGGTCCAGATTCGGTTGCTGAAACTA

KJ699390 GAAGAAAGTGCGCAGGAGAACCACTGGTCCAGATTCGGTTGCTGAAACTA

AY728807 GAAGAAAGTGCGCAGGAGAAGCACTGGTCCTGATTCGGTTGCTGAAACCA

JN107537 GAAGAAAGTGCGCAGGAGAAGCACTGGTCCTGATTCGGTTGCTGAAACCA

JQ693159 GAAGAAAGTGCGCAGGAGAAGCACTGGTCCTGATTCGGTTGCTGAAACCA

PI428208U GAAGAAAGTGCGCAGGAGAAGCACCGGT-CTGATTCGGTTGCTGAAACCA

PI487236S GAAGAAAGTGCGCAGGAGAAGCACCGGT-CTGATTCGGTTGCTGAAACCA

PI662241U GAAGAAAGTGCGCAGGAGAAGCACCGGT-CTGATTCGGTTGCTGAAACCA

PI428323U GAAGAAAGTGCGCAGGAGAAGCACCGGT-CTGATTCGGTTGCTGAAACCA

PI538726U GAAGAAAGTGCGCAGGAGAAGCACCGGT-CTGATTCGGTTGCTGAAACCA

PI428231U GAAGAAAGTGCGCAGGAGAAGCACCGGT-CTGATTCGGTTGCTGAAACCA

PI554297S GAAGAAAGTGCGCAGGAGAAGCACCGGTCCTGATTCGGTTGCTGAAACCA

PI355519M GAAGAAAGTGCGCAGGAGAAGCACCGGTCCTGATTCGGTTGCTGAAACCA

PI487235S GAAGAAAGTGCGCAGGAGAAGCACCGGTCCTGATTCGGTTGCTGAAACCA

PI554323 GAAGAAAGTGCGCAGGAGAAGCACCGGTCCTGATTCGGTTGCTGAAACCA

PI508260 GAAGAAAGTGCGCAGGAGAAGCACCGGTCCTGATTCGGTTGCTGAAACCA

PI487268U GAAGAAAGTGCGCAGGAGAAGCACCGGTCCTGATTCGGTTGCTGAAACCA

PI662238U GAAGAAAGTGCGCAGGAGAAGCACCGGTCCTGATTCGGTTGCTGAAACCA

PI369608S GAAGAAAGTGCGCAGGAGAAGCACCGGTCCTGATTCGGTTGCTGAAACCA

PI486263S GAAGAAAGTGCGCAGGAGAAGCACCGGTCCTGATTCGGTTGCTGAAACCA

PI538728U GAAGAAAGTGCGCAGGAGAAGCACCGGTCCTGATTCGGTTGCTGAAACCA

PI428241U GAAGAAAGTGCGCAGGAGAAGCACCGGTCCTGATTCGGTTGCTGAAACCA

PI428237U GAAGAAAGTGCGCAGGAGAAGCACCGGTCCTGATTCGGTTGCTGAAACCA

PI219867S GAAGAAAGTGCGCAGGAGAAGCACCGGTCCTGATTCGGTTGCTGAAACCA

PI170204S GAAGAAAGTGCGCAGGAGAAGCACCGGTCCTGATTCGGTTGCTGAAACCA

PI418582M GAAGAAAGTGCGCAGGAGAAGCACCGGTCCTGATTCGGTTGCTGAAACCA

PI428287U GAAGAAAGTGCGCAGGAGAAGCACCGGTCCTGATTCGGTTGCTGAAACCA

PI603255 GAAGAAAGTGCGCAGGAGAAGCACCGGTCCTGATTCGGTTGCTGAAACCA

PI452130 GAAGAAAGTGCGCAGGAGAAGCACCGGTCCTGATTCGGTTGCTGAAACCA

PI428215U GAAGAAAGTGCGCAGGAGAAGCACCGGTCCTGATTCGGTTGCTGAAACCA

PI428180U GAAGAAAGTGCGCAGGAGAAGCACCGGTCCTGATTCGGTTGCTGAAACCA

PI662264U GAAGAAAGTGCGCAGGAGAAGCACCGGTCGTGATTCGGTTGCTGAAACCA

PI220642 GAAGAAAGTGCGCAGGAGAAGCACTGGTCCTGATTCGGTTGCTGAAACCA

PI317392 GAAGAAAGTGCGCAGGAGAAGCACTGGT-CTGATTCGGTTGCTGAAACCA

DQ195070 GAAGAAAGTGCGCAGGAGAAGCACCGGTCCTGATTCGGTTGCTGAAACCA

DQ022952 GAAGAAAGTGCGCAGGAGAAGCACCGGTCCTGATTCGGTTGCTGAAACCA

PI662242U GAAGAAAGTGCGCAGGAGAAGCACCGGTCCTGATTCGGTTGCTGAAACCA

cltr17668U GAAGAAAGTGCGCAGGAGAAGCACCGGTCCTGATTCGGTTGCTGAAACCA

PI662239U GAAGAAAGTGCGCAGGAGAAGCACCGGTCCTGATTCGGTTGCTGAAACCA

PI487267U GAAGAAAGTGCGCAGGAGAAGCACTGGT-CTGATTCGGTTGCTGAAACCA

PI352486M GAAGAAAGTGCGCAGGAGAAGCACCGGTCCTGATTCGGTTGCTGAAACCA

PI277130M GAAGAAAGTGCGCAGGAGAAGCACTGGT-CTGATTCGGTTGCTGAAACCA

PI362610M GAAGAAAGTGCGCAGGAGAAGCACTGGTCCTGATTCGGTTGCTGAAACCA

PI573452S GAAGAAAGTGCGCAGGAGAAGCACTGGTCCTGATTCGGTTGCTGAAACCA

cltr14520M GAAGAAAGTGCGCAGGAGAAGCACCGGTCCTGATTCGGTTGCTGAAACCA

PI190946M GAAGAAAGTGCGCAGGAGAAGCACCGGT-CTGATTCGGTTGCTGAAACCA

PI191383M GAAGAAAGTGCGCAGGAGAAGCACCGGTCCTGATTCGGTTGCTGAAACCA

PI307984M GAAGAAAGTGCGCAGGAGAAGCACTGGT-CTGATTCGGTTGCTGAAACCA

PI343181M GAAGAAAGTGCGCAGGAGAAGCACTGGT-CTGATTCGGTTGCTGAAACCA

PI190915M GAAGAAAGTGCGCAGGAGAAGCACTGGTCCTGATTCGGTTGCTGAAACCA

PI225164M GAAGAAAGTGCGCAGGAGAAGCACTGGTCCTGATTCGGTTGCTGAAACCA

PI237659M GAAGAAAGTGCGCAGGAGAAGCACTGGTCCTGATTCGGTTGCTGAAACCA

PI265008M GAAGAAAGTGCGCAGGAGAAGCACTGGTCCTGATTCGGTTGCTGAAACCA

PI286068M GAAGAAAGTGCGCAGGAGAAGCACTGGTCCTGATTCGGTTGCTGAAACCA

PI306543M GAAGAAAGTGCGCAGGAGAAGCACTGGTCCTGATTCGGTTGCTGAAACCA

PI326317M GAAGAAAGTGCGCAGGAGAAGCACTGGTCCTGATTCGGTTGCTGAAACCA

PI393493S GAAGAAAGTGCGCAGGAGAAGCACTGGTCCTGATTCGGTTGCTGAAACCA

PI393496M GAAGAAAGTGCGCAGGAGAAGCACTGGTCCTGATTCGGTTGCTGAAACCA

PI427927M GAAGAAAGTGCGCAGGAGAAGCACTGGTCCTGATTCGGTTGCTGAAACCA

PI486275 GAAGAAAGTGCGCAGGAGAAGCACTGGTCCTGATTCGGTTGCTGAAACCA

PI511379 GAAGAAAGTGCGCAGGAGAAGCACTGGTCCTGATTCGGTTGCTGAAACCA

PI10474M GAAGAAAGTGCGCAGGAGAAGCACTGGTCGTGATTCGGTTGCTGAAACCA

PI452131 GAAGAAAGTGCGCAGGAGAAGCACCGGTCCTGATTCGGTTGCTGAAACCA

PI560720M GAAGAAAGTGCGCAGGAGAAGCACCGGTCCTGATTCGGTTGCTGAAACCA

PI573450S GAAGAAAGTGCGCAGGAGAAGCACCGGTCGTGATTCGGTTGCTGAAACCA

PI94740M GAAGAAAGTGCGCAGGAGAAGCACCGGTCCTGATTCGGTTGCTGAAACCA

PI428183U GAAGAAAGTGCGCAGGAGAAGCACCGGT-CTGATTCGGTTGCTGAAACCA

PI538727U GAAGAAAGTGCGCAGGAGAAGCACCGGTCCTGATTCGGTTGCTGAAACCA

PI554324 GAAGAAAGTGCGCAGGAGAAGCACCGGTCCTGATTCGGTTGCTGAAACCA

PI369602S GAAGAAAGTGCGCAGGAGAAGCACCGGTCCTGATTCGGTTGCTGAAACCA

PI487237S GAAGAAAGTGCGCAGGAGAAGCACCGGTCCTGATTCGGTTGCTGAAACCA

PI272561M GAAGAAAGTGCGCAGGAGAAGCACCGGTCCTGATTCGGTTGCTGAAACCA

PI554320 GAAGAAAGTGCGCAGGAGAAGCACTGGT-CTGATTCGGTTGCTGAAACCA

PI603230 GAAGAAAGTGCGCAGGAGAAGCACTGGTCCTGATTCGGTTGCTGAAACCA

PI168804M GAAGAAAGTGCGCAGGAGAAGCACTGGTCCTGATTCTGTTGCTGAAACCA

DQ195068 GAAGAAAGTGCGCAGGAGAAGCACTGGTCCTGATTCGGTTGCTGAAACCA

AF303376 GAAGAAAGTGCGCAGGAGAAGCACTGGTCCTGATTCGGTTGCTGAAACCA

DQ022953 GAAGAAAGTGCGCAGGAGAAGCACTGGTCCTGATTCGGTTGCTGAAACCA

FR719742 GAAGAAAGTGCGCAGGAGAAGCACTGGTCCCGATTCGGTTGCTGAAACCA

KM388515 GAAGAAAGTGCGCAGGAGAAGCACTGGTCCCGATTCGGTTGCTGAAACCA

KM388516 GAAGAAAGTGCGCAGGAGAAGCACTGGTCCCGATTCGGTTGCTGAAACCA

KM388514 GAAGAAAGTGCGCAGGAGAAGCACTGGTCCCGATTCGGTTGCTGAAACCA

HM746657 GAAGAAAGTGCGCAGGAGAAGCACTGGTCCCGATTCGGTTGCTGAAACCA

PI486264S GAAGAAAGTGCGCAGGAGAAGCACTGGTCCTGATTCGGTTGCTGAAACCA

KM388518 GAAGAAAGTGCGCAGGAGAAGCACTGGTCCCGATTCGGTTGCTGAAACCA

KM388517 GAAGAGAGTGCGCAGGAGAAGCACTGGTCCCGATTCGGTTGCTGAAACCA

KM388519 GAAGAAAGTGCGCAGGAGAAGCACTGGTCCCGATTCGGTTGCTGAAACCA

KM388520 GAAGAAAGTGCGCAGGAGAAGCACTGGTCCCGATTCGGTTGCTGAAACCA

KJ534637 GAAGAAAGTGCGCAGGAGAAGCACTGGTCCCGATTCGGTTGCTGAAACCA

KM388521 GAAGAAAGTGCGCAGGAGAAGCACTGGTCCCGATTCGGTTGCTGAAACCA

XM_003569037 GAAGAAAGTGCGCAGGAGAAGCACTGGCCCTGATTCGATTGCTGAAACCA

HQ647359 TCAAGAAGTGGAAGGAGCAAAACCAGAAGCTCCAGCAAGAGAATGGATCC

AK376344 TCAAGAAGTGGAAGGAGCAAAACCAGAAGCTCCAGCAAGAGAATGGATCC

DQ012941 TCAAGAAGTGGAAGGAGCAAAACCAGAAGCTCCAGCAAGAGAATGGATCC

KJ699390 TCAAGAAGTGGAAGGAGCAAAACCAGAAGCTCCAGCAAGAGAATGGATCC

AY728807 TCAAGAAGTGGAAGGAGCAAAACCAGAAGCTCCAGCAAGAGAATGGATCC

JN107537 TCAAGAAGTGGAAGGAGCAAAACCAGAAGCTCCAGCAAGAGAATGGATCC

JQ693159 TCAAGAAGTGGAAGGAGCAAAACCAGAAGCTCCAGCAAGAGAATGGATCC

PI428208U TCAAGAAGTGGAAGGAGGAAAACCAGAAGCTCCAGCAAGAGAATGGATCC

PI487236S TCAAGAAGTGGAAGGAGGAAAACCAGAAGCTCCAGCAAGAGAATGGATCC

PI662241U TCAAGAAGTGGAAGGAGGAAAACCAGAAGCTCCAGCAAGAGAATGGATCC

PI428323U TCAAGAAGTGGAAGGAGGAAAACCAGAAGCTCCAGCAAGAGAATGGATCC

PI538726U TCAAGAAGTGGAAGGAGGAAAACCAGAAGCTCCAGCAAGAGAATGGATCC

PI428231U TCAAGAAGTGGAAGGAGGAAAACCAGAAGCTCCAGCAAGAGAATGGATCC

PI554297S TCAAGAAGTGGAAGGAGGAAAACCAGAAGCTCCAGCAAGAGAATGGATCC

PI355519M TCAAGAAGTGGAAGGAGGAAAACCAGAAGCTCCAGCAAGAGAATGGATCC

PI487235S TCAAGAAGTGGAAGGAGGAAAACCAGAAGCTCCAGCAAGAGAATGGATCC

PI554323 TCAAGAAGTGGAAGGAGGAAAACCAGAAGCTCCAGCAAGAGAATGGATCC

PI508260 TCAAGAAGTGGAAGGAGGAAAACCAGAAGCTCCAGCAAGAGAATGGATCC

PI487268U TCAAGAAGTGGAAGGAGGAAAACCAGAAGCTCCAGCAAGAGAATGGATCC

PI662238U TCAAGAAGTGGAAGGAGGAAAACCAGAAGCTCCAGCAAGAGAATGGATCC

PI369608S TCAAGAAGTGGAAGGAGGAAAACCAGAAGCTCCAGCAAGAGAATGGATCC

PI486263S TCAAGAAGTGGAAGGAGGAAAACCAGAAGCTCCAGCAAGAGAATGGATCC

PI538728U TCAAGAAGTGGAAGGAGGAAAACCAGAAGCTCCAGCAAGAGAATGGATCC

PI428241U TCAAGAAGTGGAAGGAGGAAAACCAGAAGCTCCAGCAAGAGAATGGATCC

PI428237U TCAAGAAGTGGAAGGAGGAAAACCAGAAGCTCCAGCAAGAGAATGGATCC

PI219867S TCAAGAAGTGGAAGGAGGAAAACCAGAAGCTCCAGCAAGAGAATGGATCC

PI170204S TCAAGAAGTGGAAGGAGGAAAACCAGAAGCTCCAGCAAGAGAATGGATCC

PI418582M TCAAGAAGTGGAAGGAGGAAAACCAGAAGCTCCAGCAAGAGAATGGATCC

PI428287U TCAAGAAGTGGAAGGAGGAAAACCAGAAGCTCCAGCAAGAGAATGGATCC

PI603255 TCAAGAAGTGGAAGGAGGAAAACCAGAAGCTCCAGCAAGAGAATGGATCC

PI452130 TCAAGAAGTGGAAGGAGGAAAACCAGAAGCTCCAGCAAGAGAATGGATCC

PI428215U TCAAGAAGTGGAAGGAGGAAAACCAGAAGCTCCAGCAAGAGAATGGATCC

PI428180U TCAAGAAGTGGAAGGAGGAAAACCAGAAGCTCCAGCAAGAGAATGGATCC

PI662264U TCAAGAAGTGGAAGGAGGAAAACCAGAAGCTCCAGCAAGAGAATGGATCC

PI220642 TCAAGAAGTGGAAGGAGGAAAACCAGAAGCTCCAGCAAGAGAATGGATCC

PI317392 TCAAGAAGTGGAAGGAGGAAAACCAGAAGCTCCAGCAAGAGAATGGATCC

DQ195070 TCAAGAAGTGGAAGGAGGAAAACCAGAAGCTCCAGCAAGAGAATGGATCC

DQ022952 TCAAGAAGTGGAAGGAGGAAAACCAGAAGCTCCAGCAAGAGAATGGATCC

PI662242U TCAAGAAGTGGAAGGAGGAAAACCAGAAGCTCCAGCAAGAGAATGGATCC

cltr17668U TCAAGAAGTGGAAGGAGGAAAACCAGAAGCTCCAGCAAGAGAATGGATCC

PI662239U TCAAGAAGTGGAAGGAGGAAAACCAGAAGCTCCAGCAAGAGAATGGATCC

PI487267U TCAAGAAGTGGAAGGAGGAAAACCAGAAGCTCCAGCAAGAGAATGGATCC

PI352486M TCAAGAAGTGGAAGGAGGAAAACCAGAAGCTCCAGCAAGAGAATGGATCC

PI277130M TCAAGAAGTGGAAGGAGGAAAACCAGAAGCTCCAGCAAGAGAATGGATCC

PI362610M TCAAGAAGTGGAAGGAGGAAAACCAGAAGCTCCAGCAAGAGAATGGATCC

PI573452S TCAAGAAGTGGAAGGAGGAAAACCAGAAGCTCCAGCAAGAGAATGGATCC

cltr14520M TCAAGAAGTGGAAGGAGGAAAACCAGAAGCTCCAGCAAGAGAATGGATCC

PI190946M TCAAGAAGTGGAAGGAGGAAAACCAGAAGCTCCAGCAAGAGAATGGATCC

PI191383M TCAAGAAGTGGAAGGAGGAAAACCAGAAGCTCCAGCAAGAGAATGGATCC

PI307984M TCAAGAAGTGGAAGGAGGAAAACCAGAAGCTCCAGCAAGAGAATGGATCC

PI343181M TCAAGAAGTGGAAGGAGGAAAACCAGAAGCTCCAGCAAGAGAATGGATCC

PI190915M TCAAGAAGTGGAAGGAGGAAAACCAGAAGCTCCAGCAAGAGAATGGATCC

PI225164M TCAAGAAGTGGAAGGAGGAAAACCAGAAGCTCCAGCAAGAGAATGGATCC

PI237659M TCAAGAAGTGGAAGGAGGAAAACCAGAAGCTCCAGCAAGAGAATGGATCC

PI265008M TCAAGAAGTGGAAGGAGGAAAACCAGAAGCTCCAGCAAGAGAATGGATCC

PI286068M TCAAGAAGTGGAAGGAGGAAAACCAGAAGCTCCAGCAAGAGAATGGATCC

PI306543M TCAAGAAGTGGAAGGAGGAAAACCAGAAGCTCCAGCAAGAGAATGGATCC

PI326317M TCAAGAAGTGGAAGGAGGAAAACCAGAAGCTCCAGCAAGAGAATGGATCC

PI393493S TCAAGAAGTGGAAGGAGGAAAACCAGAAGCTCCAGCAAGAGAATGGATCC

PI393496M TCAAGAAGTGGAAGGAGGAAAACCAGAAGCTCCAGCAAGAGAATGGATCC

PI427927M TCAAGAAGTGGAAGGAGGAAAACCAGAAGCTCCAGCAAGAGAATGGATCC

PI486275 TCAAGAAGTGGAAGGAGGAAAACCAGAAGCTCCAGCAAGAGAATGGATCC

PI511379 TCAAGAAGTGGAAGGAGGAAAACCAGAAGCTCCAGCAAGAGAATGGATCC

PI10474M TCAAGAAGTGGAAGGAGGAAAACCAGAAGCTCCAGCAAGAGAATGGATCC

PI452131 TCAAGAAGTGGAAGGAGGAAAACCAGAAGCTCCAGCAAGAGAATGGATCC

PI560720M TCAAGAAGTGGAAGGAGGAAAACCAGAAGCTCCAGCAAGAGAATGGATCC

PI573450S TCAAGAAGTGGAAGGAGGAAAACCAGAAGCTCCAGCAAGAGAATGGATCC

PI94740M TCAAGAAGTGGAAGGAGGAAAACCAGAAGCTCCAGCAAGAGAATGGATCC

PI428183U TCAAGAAGTGGAAGGAGGAAAACCAGAAGCTCCAGCAAGAGAATGGATCC

PI538727U TCAAGAAGTGGAAGGAGGAAAACCAGAAGCTCCAGCAAGAGAATGGATCC

PI554324 TCAAGAAGTGGAAGGAGGAAAACCAGAAGCTCCAGCAAGAGAATGGATCC

PI369602S TCAAGAAGTGGAAGGAGGAAAACCAGAAGCTCCAGCAAGAGAATGGATCC

PI487237S TCAAGAAGTGGAAGGAGGAAAACCAGAAGCTCCAGCAAGAGAATGGATCC

PI272561M TCAAGAAGTGGAAGGAGGAAAACCAGAAGCTCCAGCAAGAGAATGGATCC

PI554320 TCAAGAAGTGGAAGGAGGAAAACCAGAAGCTCCAGCAAGAGAATGGATCC

PI603230 TCAAGAAGTGGAAGGAGGAAAACCAGAAGCTCCAGCAAGAGAATGGATCC

PI168804M TCAAGAAGTGGAAGGAGGAAAACCAGAAGCTCCAGCAAGAGAATGGATCC

DQ195068 TCAAGAAGTGGAAGGAGGAAAACCAGAAGCTCCAGCAAGAGAATGGATCC

AF303376 TCAAGAAGTGGAAGGAGGAAAACCAGAAGCTCCAGCAAGAGAATGGATCC

DQ022953 TCAAGAAGTGGAAGGAGGAAAACCAGAAGCTCCAGCAAGAGAATGGATCC

FR719742 TCAAGAAGTGGAAGGAGGAAAACCAGAAGCTCCAGCAAGAGAATGGATCC

KM388515 TCAAGAAGTGGAAGGAGGAAAACCAGAAGCTCCAGCAAGAGAATGGATCC

KM388516 TCAAGAAGTGGAAGGAGGAAAACCAGAAGCTCCAGCAAGAGAATGGATCC

KM388514 TCAAGAAGTGGAAGGAGGAAAACCAGAAGCTCCAGCAAGAGAATGGATCC

HM746657 TCAAGAAGTGGAAGGAGGAAAACCAGAAGCTCCAGCAAGAGAATGGATCC

PI486264S TCAAGAAGTGGAAGGAGGAAAACCAGAAGCTCCTGCAAGAGAATGGATCC

KM388518 TCAAGAAGTGGAAGGAGCAAAACCAGAAGCTCCAGCAAGAGAATGGATCC

KM388517 TCAAGAAGTGGAAGGAGCAAAACCAGAAGCTCCAGCAAGAGAATGGATCC

KM388519 TCAAGAAGTGGAAGGAGCAAAACCAGAAGCTCCAGCAAGAGAATGGATCC

KM388520 TCAAGAAGTGGAAGGAGCAAAACCAGAAGCTCCAGCAAGAGAATGGATCC

KJ534637 TCAAGAAGTGGAAGGAGCAAAACCAGAAGCTCCAGCAAGAGAATGGATTC

KM388521 TCAAGAAGTGGAAGGAGCAAAACCAGAAGCTCCAGCAAGAGAATGGATCC

XM_003569037 TCAAGAAATGGAAGGAGCAAAATCAGAAGCTCCAGGGAGAGAATGGACCC

HQ647359 CGGAAAGCGCCCGCCAAGGGTTCCAAGAAAGGGTGCATGGCAGGGAAAGG

AK376344 CGGAAAGCGCCCGCCAAGGGTTCCAAGAAAGGGTGCATGGCAGGGAAAGG

DQ012941 CGGAAAGCGCCCGCCAAGGGTTCCAAGAAAGGGTGCATGGCAGGGAAAGG

KJ699390 CGGAAAGCGCCCGCCAAGGGTTCCAAGAAAGGGTGCATGGCAGGGAAAGG

AY728807 CGGAAAGCGCCTGCCAAGGGTTCCAAGAAAGGGTGCATGGCAGGGAAAGG

JN107537 CGGAAAGCGCCTGCCAAGGGTTCCAAGAAAGGGTGCATGGCAGGGAAAGG

JQ693159 CGGAAAGCGCCTGCCAAGGGTTCCAAGAAAGGGTGCATGGCAGGGAAAGG

PI428208U CGGAAAGCACCGGCCAAGGGTTCCAAGAAAGGGTGCATGGCAGGGAAAGG

PI487236S CGGAAAGCACCGGCCAAGGGTTCCAAGAAAGGGTGCATGGCAGGGAAAGG

PI662241U CGGAAAGCACCGGCCAAGGGTTCCAAGAAAGGGTGCATGGCAGGGAAAGG

PI428323U CGGAAAGCACCGGCCAAGGGTTCCAAGAAAGGGTGCATGGCAGGGAAAGG

PI538726U CGGAAAGCACCGGCCAAGGGTTCCAAGAAAGGGTGCATGGCAGGGAAAGG

PI428231U CGGAAAGCACCGGCCAAGGGTTCCAAGAAAGGGTGCATGGCAGGGAAAGG

PI554297S CGGAAAGCACCGGCCAAGGGTTCCAAGAAAGGGTGCATGGCAGGGAAAGG

PI355519M CGGAAAGCACCGGCCAAGGGTTCCAAGAAAGGGTGCATGGCAGGGAAAGG

PI487235S CGGAAAGCACCGGCCAAGGGTTCCAAGAAAGGGTGCATGGCAGGGAAAGG

PI554323 CGGAAAGCACCGGCCAAGGGTTCCAAGAAAGGGTGCATGGCAGGGAAAGG

PI508260 CGGAAAGCACCGGCCAAGGGTTCCAAGAAAGGGTGCATGGCAGGGAAAGG

PI487268U CGGAAAGCACCGGCCAAGGGTTCCAAGAAAGGGTGCATGGCAGGGAAAGG

PI662238U CGGAAAGCACCGGCCAAGGGTTCCAAGAAAGGGTGCATGGCAGGGAAAGG

PI369608S CGGAAAGCACCGGCCAAGGGTTCCAAGAAAGGGTGCATGGCAGGGAAAGG

PI486263S CGGAAAGCACCGGCCAAGGGTTCCAAGAAAGGGTGCATGGCAGGGAAAGG

PI538728U CGGAAAGCACCGGCCAAGGGTTCCAAGAAAGGGTGCATGGCAGGGAAAGG

PI428241U CGGAAAGCACCGGCCAAGGGTTCCAAGAAAGGGTGCATGGCAGGGAAAGG

PI428237U CGGAAAGCACCGGCCAAGGGTTCCAAGAAAGGGTGCATGGCAGGGAAAGG

PI219867S CGGAAAGCACCGGCCAAGGGTTCCAAGAAAGGGTGCATGGCAGGGAAAGG

PI170204S CGGAAAGCACCGGCCAAGGGTTCCAAGAAAGGGTGCATGGCAGGGAAAGG

PI418582M CGGAAAGCACCGGCCAAGGGTTCCAAGAAAGGGTGCATGGCAGGGAAAGG

PI428287U CGGAAAGCACCGGCCAAGGGTTCCAAGAAAGGGTGCATGGCAGGGAAAGG

PI603255 CGGAAAGCACCGGCCAAGGGTTCCAAGAAAGGGTGCATGGCAGGGAAAGG

PI452130 CGGAAAGCACCGGCCAAGGGTTCCAAGAAAGGGTGCATGGCAGGGAAAGG

PI428215U CGGAAAGCACCGGCCAAGGGTTCCAAGAAAGGGTGCATGGCAGGGAAAGG

PI428180U CGGAAAGCACCGGCCAAGGGTTCCAAGAAAGGGTGCATGGCAGGGAAAGG

PI662264U CGGAAAGCACCGGCCAAGGGTTCCAAGAAAGGGTGCATGGCAGGGAAAGG

PI220642 CGGAAAGCACCGGCCAAGGGTTCCAAGAAAGGGTGCATGGCAGGGAAAGG

PI317392 CGGAAAGCACCGGCCAAGGGTTCCAAGAAAGGGTGCATGGCAGGGAAAGG

DQ195070 CGGAAAGCACCGGCCAAGGGTTCCAAGAAAGGGTGCATGGCAGGGAAAGG

DQ022952 CGGAAAGCACCGGCCAAGGGTTCCAAGAAAGGGTGCATGGCAGGGAAAGG

PI662242U CGGAAAGCACCGGCCAAGGGTTCCAAGAAAGGGTGCATGGCAGGGAAAGG

cltr17668U CGGAAAGCACCGGCCAAGGGTTCCAAGAAAGGGTGCATGGCAGGGAAAGG

PI662239U CGGAAAGCACCGGCCAAGGGTTCCAAGAAAGGGTGCATGGCAGGGAAAGG

PI487267U CGGAAAGCACCGGCCAAGGGTTCCAAGAAAGGGTGCATGGCAGGGAAAGG

PI352486M CGGAAAGCACCGGCCAAGGGTTCCAAGAAAGGGTGCATGGCAGGGAAAGG

PI277130M CGGAAAGCACCGGCCAAGGGTTCCAAGAAAGGGTGCATGGCAGGGAAAGG

PI362610M CGGAAAGCACCGGCCAAGGGTTCCAAGAAAGGGTGCATGGCAGGGAAAGG

PI573452S CGGAAAGCACCGGCCAAGGGTTCCAAGAAAGGGTGCATGGCAGGGAAAGG

cltr14520M CGGAAAGCACCGGCCAAGGGTTCCAAGAAAGGGTGCATGGCAGGGAAAGG

PI190946M CGGAAAGCACCGGCCAAGGGTTCCAAGAAAGGGTGCATGGCAGGGAAAGG

PI191383M CGGAAAGCACCGGCCAAGGGTTCCAAGAAAGGGTGCATGGCAGGGAAAGG

PI307984M CGGAAAGCACCGGCCAAGGGTTCCAAGAAAGGGTGCATGGCAGGGAAAGG

PI343181M CGGAAAGCACCGGCCAAGGGTTCCAAGAAAGGGTGCATGGCAGGGAAAGG

PI190915M CGGAAAGCACCGGCCAAGGGTTCCAAGAAAGGGTGCATGGCAGGGAAAGG

PI225164M CGGAAAGCACCGGCCAAGGGTTCCAAGAAAGGGTGCATGGCAGGGAAAGG

PI237659M CGGAAAGCACCGGCCAAGGGTTCCAAGAAAGGGTGCATGGCAGGGAAAGG

PI265008M CGGAAAGCACCGGCCAAGGGTTCCAAGAAAGGGTGCATGGCAGGGAAAGG

PI286068M CGGAAAGCACCGGCCAAGGGTTCCAAGAAAGGGTGCATGGCAGGGAAAGG

PI306543M CGGAAAGCACCGGCCAAGGGTTCCAAGAAAGGGTGCATGGCAGGGAAAGG

PI326317M CGGAAAGCACCGGCCAAGGGTTCCAAGAAAGGGTGCATGGCAGGGAAAGG

PI393493S CGGAAAGCACCGGCCAAGGGTTCCAAGAAAGGGTGCATGGCAGGGAAAGG

PI393496M CGGAAAGCACCGGCCAAGGGTTCCAAGAAAGGGTGCATGGCAGGGAAAGG

PI427927M CGGAAAGCACCGGCCAAGGGTTCCAAGAAAGGGTGCATGGCAGGGAAAGG

PI486275 CGGAAAGCACCGGCCAAGGGTTCCAAGAAAGGGTGCATGGCAGGGAAAGG

PI511379 CGGAAAGCACCGGCCAAGGGTTCCAAGAAAGGGTGCATGGCAGGGAAAGG

PI10474M CGGAAAGCACCGGCCAAGGGTTCCAAGAAAGGGTGCATGGCAGGGAAAGG

PI452131 CGGAAAGCACCGGCCAAGGGTTCCAAGAAAGGGTGCATGGCAGGGAAAGG

PI560720M CGGAAAGCACCGGCCAAGGGTTCCAAGAAAGGGTGCATGGCAGGGAAAGG

PI573450S CGGAAAGCACCGGCCAAGGGTTCCAAGAAAGGGTGCATGGCAGGGAAAGG

PI94740M CGGAAAGCACCGGCCAAGGGTTCCAAGAAAGGGTGCATGGCAGGGAAAGG

PI428183U CGGAAAGCACCGGCCAAGGGTTCCAAGAAAGGGTGCATGGCAGGGAAAGG

PI538727U CGGAAAGCACCGGCCAAGGGTTCCAAGAAAGGGTGCATGGCAGGGAAAGG

PI554324 CGGAAAGCACCGGCCAAGGGTTCCAAGAAAGGGTGCATGGCAGGGAAAGG

PI369602S CGGAAAGCACCGGCCAAGGGTTCCAAGAAAGGGTGCATGGCAGGGAAAGG

PI487237S CGGAAAGCACCGGCCAAGGGTTCCAAGAAAGGGTGCATGGCAGGGAAAGG

PI272561M CGGAAAGCACCGGCCAAGGGTTCCAAGAAAGGGTGCATGGCAGGGAAAGG

PI554320 CGGAAAGCACCGGCCAAGGGTTCCAAGAAAGGGTGCATGGCAGGGAAAGG

PI603230 CGGAAAGCACCGGCCAAGGGTTCCAAGAAAGGGTGCATGGCAGGGAAAGG

PI168804M CGGAAAGCACCGGCCAAGGGTTCCAAGAAAGGGTGCATGGCAGGGAAAGG

DQ195068 CGGAAAGCACCGGCCAAGGGTTCCAAGAAAGGGTGCATGGCAGGGAAAGG

AF303376 CGGAAAGCACCGGCCAAGGGTTCCAAGAAAGGGTGCATGGCAGGGAAAGG

DQ022953 CGGAAAGCACCGGCCAAGGGTTCCAAGAAAGGGTGCATGGCAGGGAAAGG

FR719742 CGGAAAGCACCGGCCAAGGGTTCCAAGAAAGGGTGCATGGCAGGGAAAGG

KM388515 CGGAAAGCACCGGCCAAGGGTTCCAAGAAAGGGTGCATGGCAGGGAAAGG

KM388516 CGGAAAGCACCGGCCAAGGGTTCCAAGAAAGGGTGCATGGCAGGGAAAGG

KM388514 CGGAAAGCACCGGCCAAGGGTTCCAAGAAAGGGTGCATGGCAGGGAAAGG

HM746657 CGGAAAGCACCGGCCAAGGGTTCCAAGAAAGGGTGCATGGCAGGGAAAGG

PI486264S CGGAAAGCACCGGCCAAGGGTTCCAAGAAAGGGTGCATGGCAGGGAAAGG

KM388518 CGGAAAGCACCGGCCAAGGGTTCCAAGAAAGGGTGCATGGCAGGGAAAGG

KM388517 CGGAAAGCACCGGCCAAGGGTTCCAAGAAAGGGTGCATGGCAGGGAAAGG

KM388519 CGGAAAGCACCGGCCAAGGGTTCCAAGAAAGGGTGCATGGCAGGGAAAGG

KM388520 CGGAAAGCACCGGCCAAGGGTTCCAAGAAAGGGTGCATGGCAGGGAAAGG

KJ534637 CGGAAGGCACCGGCCAAGGGTTCCAAGAAAGGGTGCATGGCAGGGAAAGG

KM388521 CGGAAAGCACCGGCCAAGGGTTCCAAGAAAGGGTGCATGGCAGGGAAAGG

XM_003569037 CGGAAAGCGCCGGCCAAGGGTTCCAAGAAAGGGTGCATGGCAGGGAAGGG

HQ647359 AGGTCCAGAGAATTCAAACTGCGCTTACCGCGGTGTGAGGCAGCGCACGT

AK376344 AGGTCCAGAGAATTCAAACTGCGCTTACCGCGGTGTGAGGCAGCGCACGT

DQ012941 AGGTCCAGAGAATTCAAACTGCGCTTACCGCGGTGTGAGGCAGCGCACGT

KJ699390 AGGTCCAGAGAATTCAAACTGCGCTTACCGCGGTGTGAGGCAGCGCACGT

AY728807 AGGTCCAGAGAATTCAAACTGCGCTTACCGTGGTGTGAGGCAGCGCACGT

JN107537 AGGTCCAGAGAATTCAAACTGCGCTTACCGTGGTGTGAGGCAGCGCACGT

JQ693159 AGGTCCAGAGAATTCAAACTGCGCTTACCGTGGTGTGAGGCAGCGCACGT

PI428208U AGGTCCAGAGAATTCAAACTGCGCTTACCGCGGTGTGAGGCAGAGGACGT

PI487236S AGGTCCAGAGAATTCAAACTGCGCTTACCGCGGTGTGAGGCAGAGGACGT

PI662241U AGGTCCAGAGAATTCAAACTGCGCTTACCGCGGTGTGAGGCAGAGGACGT

PI428323U AGGTCCAGAGAATTCAAACTGCGCTTACCGCGGTGTGAGGCAGAGGACGT

PI538726U AGGTCCAGAGAATTCAAACTGCGCTTACCGCGGTGTGAGGCAGAGGACGT

PI428231U AGGTCCAGAGAATTCAAACTGCGCTTACCGCGGTGTGAGGCAGAGGACGT

PI554297S AGGTCCAGAGAATTCAAACTGCGCTTACCGCGGTGTGAGGCAGAGGACGT

PI355519M AGGTCCAGAGAATTCAAACTGCGCTTACCGCGGTGTGAGGCAGAGGACGT

PI487235S AGGTCCAGAGAATTCAAACTGCGCTTACCGCGGTGTGAGGCAGAGGACGT

PI554323 AGGTCCAGAGAATTCAAACTGCGCTTACCGCGGTGTGAGGCAGAGGACGT

PI508260 AGGTCCAGAGAATTCAAACTGCGCTTACCGCGGTGTGAGGCAGAGGACGT

PI487268U AGGTCCAGAGAATTCAAACTGCGCTTACCGCGGTGTGAGGCAGAGGACGT

PI662238U AGGTCCAGAGAATTCAAACTGCGCTTACCGCGGTGTGAGGCAGAGGACGT

PI369608S AGGTCCAGAGAATTCAAACTGCGCTTACCGCGGTGTGAGGCAGAGGACGT

PI486263S AGGTCCAGAGAATTCAAACTGCGCTTACCGCGGTGTGAGGCAGAGGACGT

PI538728U AGGTCCAGAGAATTCAAACTGCGCTTACCGCGGTGTGAGGCAGAGGACGT

PI428241U AGGTCCAGAGAATTCAAACTGCGCTTACCGCGGTGTGAGGCAGAGGACGT

PI428237U AGGTCCAGAGAATTCAAACTGCGCTTACCGCGGTGTGAGGCAGAGGACGT

PI219867S AGGTCCAGAGAATTCAAACTGCGCTTACCGCGGTGTGAGGCAGAGGACGT

PI170204S AGGTCCAGAGAATTCAAACTGCGCTTACCGCGGTGTGAGGCAGAGGACGT

PI418582M AGGTCCAGAGAATTCAAACTGCGCTTACCGCGGTGTGAGGCAGAGGACGT

PI428287U AGGTCCAGAGAATTCAAACTGCGCTTACCGCGGTGTGAGGCAGAGGACGT

PI603255 AGGTCCAGAGAATTCAAACTGCGCTTACCGCGGTGTGAGGCAGAGGACGT

PI452130 AGGTCCAGAGAATTCAAACTGCGCTTACCGCGGTGTGAGGCAGAGGACGT

PI428215U AGGTCCAGAGAATTCAAACTGCGCTTACCGCGGTGTGAGGCAGAGGACGT

PI428180U AGGTCCAGAGAATTCAAACTGCGCTTACCGCGGTGTGAGGCAGAGGACGT

PI662264U AGGTCCAGAGAATTCAAACTGCGCTTACCGCGGTGTGAGGCAGAGGACGT

PI220642 AGGTCCAGAGAATTCAAACTGCGCTTACCGCGGTGTGAGGCAGAGGACGT

PI317392 AGGTCCAGAGAATTCAAACTGCGCTTACCGCGGTGTGAGGCAGAGGACGT

DQ195070 AGGTCCAGAGAATTCAAACTGCGCTTACCGCGGTGTGAGGCAGAGGACGT

DQ022952 AGGTCCAGAGAATTCAAACTGCGCTTACCGCGGTGTGAGGCAGAGGACGT

PI662242U AGGTCCAGAGAATTCAAACTGCGCTTACCGCGGTGTGAGGCAGAGGACGT

cltr17668U AGGTCCAGAGAATTCAAACTGCGCTTACCGCGGTGTGAGGCAGAGGACGT

PI662239U AGGTCCAGAGAATTCAAACTGCGCTTACCGCGGTGTGAGGCAGAGGACGT

PI487267U AGGTCCAGAGAATTCAAACTGCGCTTACCGCGGTGTGAGGCAGAGGACGT

PI352486M AGGTCCAGAGAATTCAAACTGCGCTTACCGCGGTGTGAGGCAGAGGACGT

PI277130M AGGTCCAGAGAATTCAAACTGCGCTTACCGCGGTGTGAGGCAGAGGACGT

PI362610M AGGTCCAGAGAATTCAAACTGCGCTTACCGCGGTGTGAGGCAGAGGACGT

PI573452S AGGTCCAGAGAATTCAAACTGCGCTTACCGCGGTGTGAGGCAGAGGACGT

cltr14520M AGGTCCAGAGAATTCAAACTGCGCTTACCGCGGTGTGAGGCAGAGGACGT

PI190946M AGGTCCAGAGAATTCAAACTGCGCTTACCGCGGTGTGAGGCAGAGGACGT

PI191383M AGGTCCAGAGAATTCAAACTGCGCTTACCGCGGTGTGAGGCAGAGGACGT

PI307984M AGGTCCAGAGAATTCAAACTGCGCTTACCGCGGTGTGAGGCAGAGGACGT

PI343181M AGGTCCAGAGAATTCAAACTGCGCTTACCGCGGTGTGAGGCAGAGGACGT

PI190915M AGGTCCAGAGAATTCAAACTGCGCTTACCGCGGTGTGAGGCAGAGGACGT

PI225164M AGGTCCAGAGAATTCAAACTGCGCTTACCGCGGTGTGAGGCAGAGGACGT

PI237659M AGGTCCAGAGAATTCAAACTGCGCTTACCGCGGTGTGAGGCAGAGGACGT

PI265008M AGGTCCAGAGAATTCAAACTGCGCTTACCGCGGTGTGAGGCAGAGGACGT

PI286068M AGGTCCAGAGAATTCAAACTGCGCTTACCGCGGTGTGAGGCAGAGGACGT

PI306543M AGGTCCAGAGAATTCAAACTGCGCTTACCGCGGTGTGAGGCAGAGGACGT

PI326317M AGGTCCAGAGAATTCAAACTGCGCTTACCGCGGTGTGAGGCAGAGGACGT

PI393493S AGGTCCAGAGAATTCAAACTGCGCTTACCGCGGTGTGAGGCAGAGGACGT

PI393496M AGGTCCAGAGAATTCAAACTGCGCTTACCGCGGTGTGAGGCAGAGGACGT

PI427927M AGGTCCAGAGAATTCAAACTGCGCTTACCGCGGTGTGAGGCAGAGGACGT

PI486275 AGGTCCAGAGAATTCAAACTGCGCTTACCGCGGTGTGAGGCAGAGGACGT

PI511379 AGGTCCAGAGAATTCAAACTGCGCTTACCGCGGTGTGAGGCAGAGGACGT

PI10474M AGGTCCAGAGAATTCAAACTGCGCTTACCGCGGTGTGAGGCAGAGGACGT

PI452131 AGGTCCAGAGAATTCAAACTGCGCTTACCGCGGTGTGAGGCAGAGGACGT

PI560720M AGGTCCAGAGAATTCAAACTGCGCTTACCGCGGTGTGAGGCAGAGGACGT

PI573450S AGGTCCAGAGAATTCAAACTGCGCTTACCGCGGTGTGAGGCAGAGGACGT

PI94740M AGGTCCAGAGAATTCAAACTGCGCTTACCGCGGTGTGAGGCAGAGGACGT

PI428183U AGGTCCAGAGAATTCAAACTGCGCTTACCGCGGTGTGAGGCAGAGGACGT

PI538727U AGGTCCAGAGAATTCAAACTGCGCTTACCGCGGTGTGAGGCAGAGGACGT

PI554324 AGGTCCAGAGAATTCAAACTGCGCTTACCGCGGTGTGAGGCAGAGGACGT

PI369602S AGGTCCAGAGAATTCAAACTGCGCTTACCGCGGTGTGAGGCAGAGGACGT

PI487237S AGGTCCAGAGAATTCAAACTGCGCTTACCGCGGTGTGAGGCAGAGGACGT

PI272561M AGGTCCAGAGAATTCAAACTGCGCTTACCGCGGTGTGAGGCAGAGGACGT

PI554320 AGGTCCAGAGAATTCAAACTGCGCTTACCGCGGTGTGAGGCAGAGGACGT

PI603230 AGGTCCAGAGAATTCAAACTGCGCTTACCGCGGTGTGAGGCAGAGGACGT

PI168804M AGGTCCAGAGAATTCAAACTGCGCTTACCGCGGTGTGAGGCAGAGGACGT

DQ195068 AGGTCCAGAGAATTCAAACTGCGCTTACCGCGGTGTGAGGCAGAGGACGT

AF303376 AGGTCCAGAGAATTCAAACTGCGCTTACCGCGGTGTGAGGCAGAGGACGT

DQ022953 AGGTCCAGAGAATTCAAACTGCGCTTACCGCGGTGTGAGGCAGAGGACGT

FR719742 GGGTCCAGAGAATTCAAACTGCGCTTACCGCGGTGTGAGGCAGAGGACGT

KM388515 AGGTCCAGAGAATTCAAACTGCGCTTACCGCGGTGTGAGGCAGAGGACGT

KM388516 AGGTCCAGAGAATTCAAACTGCGCTTACCGCGGTGTGAGGCAGAGGACGT

KM388514 AGGTCCAGAGAATTCAAACTGCGCTTACCGCGGTGTGAGGCATAGGACGT

HM746657 AGGTCCAGAGAATTCAAACTGCGCTTACCGCGGTGTGAGGCAGAGGACGT

PI486264S AGGTCCAGAGAATTCAAACTGCGCTTACCGCGGTGTGAGGCAGAGGACGT

KM388518 AGGTCCAGAGGATTCAAACTGCGCTTACCGCGGTGTGAGGCAGAGGACGT

KM388517 AGGTCCAGAGAATTCAAACTGCGCTTACCGCGGTGTGAGGCAGAGGACGT

KM388519 AGGTCCAGAGAATTCAAACTGCGCTTACCGCGGTGTGAGGCAGAGGACGT

KM388520 AGGTCCAGAGAATTCAAACTGCGCATACCGCGGTGTGAGGCAGAGGACGT

KJ534637 AGGTCCAGAGAATTCAAACTGCGCTTACCGCGGTGTGAGGCAGAGGACGT

KM388521 AGGCCCAGAGAATTCAAACTGCGCTTACCGCGGTGTGAGGCAGAGGACGT

XM_003569037 AGGTCCAGAAAATTCAAATTGTGCTTACCGCGGTGTGAGGCAGCGGACGT

HQ647359 GGGGCAAATGGGTGGCTGAGATCCGTGAGCCCAACCGTGGCAACCGGCTG

AK376344 GGGGCAAATGGGTGGCTGAGATCCGTGAGCCCAACCGTGGCAACCGGCTG

DQ012941 GGGGCAAATGGGTGGCTGAGATCCGTGAGCCCAACCGTGGCAACCGGCTG

KJ699390 GGGGCAAATGGGTGGCTGAGATCCGTGAGCCCAACCGTGGCAACCGGCTG

AY728807 GGGGCAAATGGGTGGCTGAGATCCGTGAGCCCAACCGTGGCAACCGGCTG

JN107537 GGGGCAAATGGGTGGCTGAGATCCGTGAGCCCAACCGTGGCAACCGGCTG

JQ693159 GGGGCAAATGGGTGGCTGAGATCCGTGAGCCCAACCGTGGCAACCGGCTG

PI428208U GGGGGAAATGGGTTGCTGAGATCCGTGAGCCCAACCGTGGCAATCGGCTG

PI487236S GGGGGAAATGGGTTGCTGAGATCCGTGAGCCCAACCGTGGCAATCGGCTG

PI662241U GGGGGAAATGGGTTGCTGAGATCCGTGAGCCCAACCGTGGCAATCGGCTG

PI428323U GGGGGAAATGGGTTGCTGAGATCCGTGAGCCCAACCGTGGCAATCGGCTG

PI538726U GGGGGAAATGGGTTGCTGAGATCCGTGAGCCCAACCGTGGCAATCGGCTG

PI428231U GGGGGAAATGGGTTGCTGAGATCCGTGAGCCCAACCGTGGCAATCGGCTG

PI554297S GGGGGAAATGGGTTGCTGAGATCCGTGAGCCCAACCGTGGCAATCGGCTG

PI355519M GGGGGAAATGGGTTGCTGAGATCCGTGAGCCCAACCGTGGCAATCGGCTG

PI487235S GGGGGAAATGGGTTGCTGAGATCCGTGAGCCCAACCGTGGCAATCGGCTG

PI554323 GGGGGAAATGGGTTGCTGAGATCCGTGAGCCCAACCGTGGCAATCGGCTG

PI508260 GGGGGAAATGGGTTGCTGAGATCCGTGAGCCCAACCGTGGCAATCGGCTG

PI487268U GGGGGAAATGGGTTGCTGAGATCCGTGAGCCCAACCGTGGCAATCGGCTG

PI662238U GGGGGAAATGGGTTGCTGAGATCCGTGAGCCCAACCGTGGCAATCGGCTG

PI369608S GGGGGAAATGGGTTGCTGAGATCCGTGAGCCCAACCGTGGCAATCGGCTG

PI486263S GGGGGAAATGGGTTGCTGAGATCCGTGAGCCCAACCGTGGCAATCGGCTG

PI538728U GGGGGAAATGGGTTGCTGAGATCCGTGAGCCCAACCGTGGCAATCGGCTG

PI428241U GGGGGAAATGGGTTGCTGAGATCCGTGAGCCCAACCGTGGCAATCGGCTG

PI428237U GGGGGAAATGGGTTGCTGAGATCCGTGAGCCCAACCGTGGCAATCGGCTG

PI219867S GGGGGAAATGGGTTGCTGAGATCCGTGAGCCCAACCGTGGCAATCGGCTG

PI170204S GGGGGAAATGGGTTGCTGAGATCCGTGAGCCCAACCGTGGCAATCGGCTG

PI418582M GGGGGAAATGGGTTGCTGAGATCCGTGAGCCCAACCGTGGCAATCGGCTG

PI428287U GGGGGAAATGGGTTGCTGAGATCCGTGAGCCCAACCGTGGCAATCGGCTG

PI603255 GGGGGAAATGGGTTGCTGAGATCCGTGAGCCCAACCGTGGCAATCGGCTG

PI452130 GGGGGAAATGGGTTGCTGAGATCCGTGAGCCCAACCGTGGCAATCGGCTG

PI428215U GGGGGAAATGGGTTGCTGAGATCCGTGAGCCCAACCGTGGCAATCGGCTG

PI428180U GGGGGAAATGGGTTGCTGAGATCCGTGAGCCCAACCGTGGCAATCGGCTG

PI662264U GGGGGAAATGGGTTGCTGAGATCCGTGAGCCCAACCGTGGCAATCGGCTG

PI220642 GGGGGAAATGGGTTGCTGAGATCCGTGAGCCCAACCGTGGCAATCGGCTG

PI317392 GGGGGAAATGGGTTGCTGAGATCCGTGAGCCCAACCGTGGCAATCGGCTG

DQ195070 GGGGGAAATGGGTTGCTGAGATCCGTGAGCCCAACCGTGGCAATCGGCTG

DQ022952 GGGGGAAATGGGTTGCTGAGATCCGTGAGCCCAACCGTGGCAATCGGCTG

PI662242U GGGGGAAATGGGTTGCTGAGATCCGTGAGCCCAACCGTGGCAATCGGCTG

cltr17668U GGGGGAAATGGGTTGCTGAGATCCGTGAGCCCAACCGTGGCAATCGGCTG

PI662239U GGGGGAAATGGGTTGCTGAGATCCGTGAGCCCAACCGTGGCAATCGGCTG

PI487267U GGGGGAAATGGGTTGCTGAGATCCGTGAGCCCAACCGTGGCAATCGGCTG

PI352486M GGGGGAAATGGGTTGCTGAGATCCGTGAGCCCAACCGTGGCAATCGGCTG

PI277130M GGGGGAAATGGGTTGCTGAGATCCGTGAGCCCAACCGTGGCAATCGGCTG

PI362610M GGGGGAAATGGGTTGCTGAGATCCGTGAGCCCAACCGTGGCAATCGGCTG

PI573452S GGGGGAAATGGGTTGCTGAGATCCGTGAGCCCAACCGTGGCAATCGGCTG

cltr14520M GGGGGAAATGGGTTGCTGAGATCCGTGAGCCCAACCGTGGCAATCGGCTG

PI190946M GGGGGAAATGGGTTGCTGAGATCCGTGAGCCCAACCGTGGCAATCGGCTG

PI191383M GGGGGAAATGGGTTGCTGAGATCCGTGAGCCCAACCGTGGCAATCGGCTG

PI307984M GGGGGAAATGGGTTGCTGAGATCCGTGAGCCCAACCGTGGCAATCGGCTG

PI343181M GGGGGAAATGGGTTGCTGAGATCCGTGAGCCCAACCGTGGCAATCGGCTG

PI190915M GGGGGAAATGGGTTGCTGAGATCCGTGAGCCCAACCGTGGCAATCGGCTG

PI225164M GGGGGAAATGGGTTGCTGAGATCCGTGAGCCCAACCGTGGCAATCGGCTG

PI237659M GGGGGAAATGGGTTGCTGAGATCCGTGAGCCCAACCGTGGCAATCGGCTG

PI265008M GGGGGAAATGGGTTGCTGAGATCCGTGAGCCCAACCGTGGCAATCGGCTG

PI286068M GGGGGAAATGGGTTGCTGAGATCCGTGAGCCCAACCGTGGCAATCGGCTG

PI306543M GGGGGAAATGGGTTGCTGAGATCCGTGAGCCCAACCGTGGCAATCGGCTG

PI326317M GGGGGAAATGGGTTGCTGAGATCCGTGAGCCCAACCGTGGCAATCGGCTG

PI393493S GGGGGAAATGGGTTGCTGAGATCCGTGAGCCCAACCGTGGCAATCGGCTG

PI393496M GGGGGAAATGGGTTGCTGAGATCCGTGAGCCCAACCGTGGCAATCGGCTG

PI427927M GGGGGAAATGGGTTGCTGAGATCCGTGAGCCCAACCGTGGCAATCGGCTG

PI486275 GGGGGAAATGGGTTGCTGAGATCCGTGAGCCCAACCGTGGCAATCGGCTG

PI511379 GGGGGAAATGGGTTGCTGAGATCCGTGAGCCCAACCGTGGCAATCGGCTG

PI10474M GGGGGAAATGGGTTGCTGAGATCCGTGAGCCCAACCGTGGCAATCGGCTG

PI452131 GGGGGAAATGGGTTGCTGAGATCCGTGAGCCCAACCGTGGCAATCGGCTG

PI560720M GGGGGAAATGGGTTGCTGAGATCCGTGAGCCCAACCGTGGCAATCGGCTG

PI573450S GGGGGAAATGGGTTGCTGAGATCCGTGAGCCCAACCGTGGCAATCGGCTG

PI94740M GGGGGAAATGGGTTGCTGAGATCCGTGAGCCCAACCGTGGCAATCGGCTG

PI428183U GGGGGAAATGGGTTGCTGAGATCCGTGAGCCCAACCGTGGCAATCGGCTG

PI538727U GGGGGAAATGGGTTGCTGAGATCCGTGAGCCCAACCGTGGCAATCGGCTG

PI554324 GGGGGAAATGGGTTGCTGAGATCCGTGAGCCCAACCGTGGCAATCGGCTG

PI369602S GGGGGAAATGGGTTGCTGAGATCCGTGAGCCCAACCGTGGCAATCGGCTG

PI487237S GGGGGAAATGGGTTGCTGAGATCCGTGAGCCCAACCGTGGCAATCGGCTG

PI272561M GGGGGAAATGGGTTGCTGAGATCCGTGAGCCCAACCGTGGCAATCGGCTG

PI554320 GGGGGAAATGGGTTGCTGAGATCCGTGAGCCCAACCGTGGCAATCGGCTG

PI603230 GGGGGAAATGGGTTGCTGAGATCCGTGAGCCCAACCGTGGCAATCGGCTG

PI168804M GGGGGAAATGGGTTGCTGAGATCCGTGAGCCCAACCGTGGCAATCGGCTG

DQ195068 GGGGGAAATGGGTTGCTGAGATCCGTGAGCCCAACCGTGGCAATCGGCTG

AF303376 GGGGGAAATGGGTTGCTGAGATCCGTGAGCCCAACCGTGGCAATCGGCTG

DQ022953 GGGGGAAATGGGTTGCTGAGATCCGTGAGCCCAACCGTGGCAATCGGCTG

FR719742 GGGGCAAATGGGTTGCTGAGATCCGTGAGCCCAACCGTGGCAATCGGCTG

KM388515 GGGGCAAATGGGTCGCTGAGATCCGTGAGCCCAACCGTGGCAACCGGCTG

KM388516 GGGGCAAATGGGTCGCTGAGATCCGTGAGCCCAACCGTGGCAACCGGCTG

KM388514 GGGGCAAATGGGTCGCTGAGATCCGTGAGCCCAACCGTGGCAACCGGCTG

HM746657 GGGGCAAATGGGTTGCTGAGATCCGTGAGCCCAACCGTGGCAACCGGCTG

PI486264S GGGGCAAATGGGTTGCTGAGATCCGTGAGCCCAACCGTGGCAACCGGCTG

KM388518 GGGGCAAATGGGTTGCTGAGATCCGTGAGCCCAACCGTGGCAACCGGCTG

KM388517 GGGGCAAATGGGTTGCTGAGATCCGTGAGCCCAACCGTGGCAACCGGCTG

KM388519 GGGGCAAATGGGTTGCTGAGATCCGTGAGCCCAACCGTGGCAACCGGCTG

KM388520 GGGGCAAATGGGTTGCTGAGATCCGTGAGCCCAACCGTGGCAACCGGCTG

KJ534637 GGGGCAAATGGGTTGCTGAGATCCGTGAGCCCAACCGTGGCAACCGGCTG

KM388521 GGGGCAAATGGGTTGCTGAGATCCGTGAGCCCAACCGTGGCAACCGGCTG

XM_003569037 GGGGCAAATGGGTCGCTGAGATCCGCGAGCCCAACCGCGGCAAGCGGCTA

HQ647359 TGGCTTGGTTCATTCCCTACCGCAGTCGAAGCTGCACGTGCATATGATGA

AK376344 TGGCTTGGTTCATTCCCTACCGCAGTCGAAGCTGCACGTGCATATGATGA

DQ012941 TGGCTTGGTTCATTCCCTACCGCAGTCGAAGCTGCACGTGCATATGATGA

KJ699390 TGGCTTGGTTCATTCCCTACCGCAGTCGAAGCTGCACGTGCATATGATGA

AY728807 TGGCTTGGTTCATTCCCTACCGCAGTCGAAGCTGCACGTGCATATGATGA

JN107537 TGGCTTGGTTCATTCCCTACCGCAGTCGAAGCTGCACGTGCATATGATGA

JQ693159 TGGCTTGGTTCATTCCCTACCGCAGTCGAAGCTGCACGTGCATATGATGA

PI428208U TGGCTTGGTTCATTCCCTACCGCAGTCGAAGCTGCACGTGCATATGATGA

PI487236S TGGCTTGGTTCATTCCCTACCGCAGTCGAAGCTGCACGTGCATATGATGA

PI662241U TGGCTTGGTTCATTCCCTACCGCAGTCGAAGCTGCACGTGCATATGATGA

PI428323U TGGCTTGGTTCATTCCCTACCGCAGTCGAAGCTGCACGTGCATATGATGA

PI538726U TGGCTTGGTTCATTCCCTACCGCAGTCGAAGCTGCACGTGCATATGATGA

PI428231U TGGCTTGGTTCATTCCCTACCGCAGTCGAAGCTGCACGTGCATATGATGA

PI554297S TGGCTTGGTTCATTCCCTACCGCAGTCGAAGCTGCACGTGCATATGATGA

PI355519M TGGCTTGGTTCATTCCCTACCGCAGTCGAAGCTGCACGTGCATATGATGA

PI487235S TGGCTTGGTTCATTCCCTACCGCAGTCGAAGCTGCACGTGCATATGATGA

PI554323 TGGCTTGGTTCATTCCCTACCGCAGTCGAAGCTGCACGTGCATATGATGA

PI508260 TGGCTTGGTTCATTCCCTACCGCAGTCGAAGCTGCACGTGCATATGATGA

PI487268U TGGCTTGGTTCATTCCCTACCGCAGTCGAAGCTGCACGTGCATATGATGA

PI662238U TGGCTTGGTTCATTCCCTACCGCAGTCGAAGCTGCACGTGCATATGATGA

PI369608S TGGCTTGGTTCATTCCCTACCGCAGTCGAAGCTGCACGTGCATATGATGA

PI486263S TGGCTTGGTTCATTCCCTACCGCAGTCGAAGCTGCACGTGCATATGATGA

PI538728U TGGCTTGGTTCATTCCCTACCGCAGTCGAAGCTGCACGTGCATATGATGA

PI428241U TGGCTTGGTTCATTCCCTACCGCAGTCGAAGCTGCACGTGCATATGATGA

PI428237U TGGCTTGGTTCATTCCCTACCGCAGTCGAAGCTGCACGTGCATATGATGA

PI219867S TGGCTTGGTTCATTCCCTACCGCAGTCGAAGCTGCACGTGCATATGATGA

PI170204S TGGCTTGGTTCATTCCCTACCGCAGTCGAAGCTGCACGTGCATATGATGA

PI418582M TGGCTTGGTTCATTCCCTACCGCAGTCGAAGCTGCACGTGCATATGATGA

PI428287U TGGCTTGGTTCATTCCCTACCGCAGTCGAAGCTGCACGTGCATATGATGA

PI603255 TGGCTTGGTTCATTCCCTACCGCAGTCGAAGCTGCACGTGCATATGATGA

PI452130 TGGCTTGGTTCATTCCCTACCGCAGTCGAAGCTGCACGTGCATATGATGA

PI428215U TGGCTTGGTTCATTCCCTACCGCAGTCGAAGCTGCACGTGCATATGATGA

PI428180U TGGCTTGGTTCATTCCCTACCGCAGTCGAAGCTGCACGTGCATATGATGA

PI662264U TGGCTTGGTTCATTCCCTACCGCAGTCGAAGCTGCACGTGCATATGATGA

PI220642 TGGCTTGGTTCATTCCCTACCGCAGTCGAAGCTGCACGTGCATATGATGA

PI317392 TGGCTTGGTTCATTCCCTACCGCAGTCGAAGCTGCACGTGCATATGATGA

DQ195070 TGGCTTGGTTCATTCCCTACTGCAGTCGAAGCTGCACGTGCATATGATGA

DQ022952 TGGCTTGGTTCATTCCCTACTGCAGTCGAAGCTGCACGTGCATATGATGA

PI662242U TGGCTTGGTTCATTCCCTACTGCAGTCGAAGCTGCACGTGCATATGATGA

cltr17668U TGGCTTGGTTCATTCCCTACTGCAGTCGAAGCTGCACGTGCATATGATGA

PI662239U TGGCTTGGTTCATTCCCTACTGCAGTCGAAGCTGCACGTGCATATGATGA

PI487267U TGGCTTGGTTCATTCCCTACTGCAGTCGAAGCTGCACGTGCATATGATGA

PI352486M TGGCTTGGTTCATTCCCTACTGCAGTCGAAGCTGCACGTGCATATGATGA

PI277130M TGGCTTGGTTCATTCCCTACTGCAGTCGAAGCTGCACGTGCATATGATGA

PI362610M TGGCTTGGTTCATTCCCTACTGCAGTCGAAGCTGCACGTGCATATGATGA

PI573452S TGGCTTGGTTCATTCCCTACCGCAGTCGAAGCTGCACGTGCATATGATGA

cltr14520M TGGCTTGGTTCATTCCCTACCGCAGTCGAAGCTGCACGTGCATATGATGA

PI190946M TGGCTTGGTTCATTCCCTACCGCAGTCGAAGCTGCACGTGCATATGATGA

PI191383M TGGCTTGGTTCATTCCCTACCGCAGTCGAAGCTGCACGTGCATATGATGA

PI307984M TGGCTTGGTTCATTCCCTACCGCAGTCGAAGCTGCACGTGCATATGATGA

PI343181M TGGCTTGGTTCATTCCCTACCGCAGTCGAAGCTGCACGTGCATATGATGA

PI190915M TGGCTTGGTTCATTCCCTACCGCAGTCGAAGCTGCACGTGCATATGATGA

PI225164M TGGCTTGGTTCATTCCCTACCGCAGTCGAAGCTGCACGTGCATATGATGA

PI237659M TGGCTTGGTTCATTCCCTACCGCAGTCGAAGCTGCACGTGCATATGATGA

PI265008M TGGCTTGGTTCATTCCCTACCGCAGTCGAAGCTGCACGTGCATATGATGA

PI286068M TGGCTTGGTTCATTCCCTACCGCAGTCGAAGCTGCACGTGCATATGATGA

PI306543M TGGCTTGGTTCATTCCCTACCGCAGTCGAAGCTGCACGTGCATATGATGA

PI326317M TGGCTTGGTTCATTCCCTACCGCAGTCGAAGCTGCACGTGCATATGATGA

PI393493S TGGCTTGGTTCATTCCCTACCGCAGTCGAAGCTGCACGTGCATATGATGA

PI393496M TGGCTTGGTTCATTCCCTACCGCAGTCGAAGCTGCACGTGCATATGATGA

PI427927M TGGCTTGGTTCATTCCCTACCGCAGTCGAAGCTGCACGTGCATATGATGA

PI486275 TGGCTTGGTTCATTCCCTACCGCAGTCGAAGCTGCACGTGCATATGATGA

PI511379 TGGCTTGGTTCATTCCCTACCGCAGTCGAAGCTGCACGTGCATATGATGA

PI10474M TGGCTTGGTTCATTCCCTACCGCAGTCGAAGCTGCACGTGCATATGATGA

PI452131 TGGCTTGGTTCATTCCCTACCGCAGTCGAAGCTGCACGTGCATATGATGA

PI560720M TGGCTTGGTTCATTCCCTACCGCAGTCGAAGCTGCACGTGCATATGATGA

PI573450S TGGCTTGGTTCATTCCCTACCGCAGTCGAAGCTGCACGTGCATATGATGA

PI94740M TGGCTTGGTTCATTCCCTACCGCAGTCGAAGCTGCACGTGCATATGATGA

PI428183U TGGCTTGGTTCATTCCCTACCGCAGTCGAAGCTGCACGTGCATATGATGA

PI538727U TGGCTTGGTTCATTCCCTACCGCAGTCGAAGCTGCACGTGCATATGATGA

PI554324 TGGCTTGGTTCATTCCCTACCGCAGTCGAAGCTGCACGTGCATATGATGA

PI369602S TGGCTTGGTTCATTCCCTACCGCAGTCGAAGCTGCACGTGCATATGATGA

PI487237S TGGCTTGGTTCATTCCCTACCGCAGTCGAAGCTGCACGTGCATATGATGA

PI272561M TGGCTTGGTTCATTCCCTACCGCAGTCGAAGCTGCACGTGCATATGATGA

PI554320 TGGCTTGGTTCATTCCCTACCGCAGTCGAAGCTGCACGTGCATATGATGA

PI603230 TGGCTTGGTTCATTCCCTACCGCAGTCGAAGCTGCACGTGCATATGATGA

PI168804M TGGCTTGGTTCATTCCCTACCGCAGTCGAAGCTGCACGTGCATATGATGA

DQ195068 TGGCTTGGTTCATTCCCTACCGCAGTCGAAGCTGCACGTGCATATGATGA

AF303376 TGGCTTGGTTCATTCCCTACCGCAGTCGAAGCTGCACGTGCATATGATGA

DQ022953 TGGCTTGGTTCATTCCCTACCGCAGTCGAAGCTGCACGTGCATATGATGA

FR719742 TGGCTTGGTTCGTTCCCTACCGCAGTCGAAGCTGCACGTGCATATGATGA

KM388515 TGGCTTGGTTCATTCCCTACCGCAGTCGAAGCTGCACGTGCATATGATGA

KM388516 TGGCTTGGTTCATTCCCTACCGCAGTCGGAGCTGCACGTGCATATGATGA

KM388514 TGGCTTGGTTCATTCCCTACCGCAGTCGAAGCTGCACGTGCATATGATGA

HM746657 TGGCTTGGTTCATTCCCTACCGCAGTCGAAGCTGCACGTGCATATGATGA

PI486264S TGGCTTGGTTCATTCCCTACCGCAGTCGAAGCTGCACGTGCATATGATGA

KM388518 TGGCTTGGTTCATTCCCTACCGCAGTCGAAGCTGCACGTGCATATGATGA

KM388517 TGGCTTGGTTCATTCCCTACCGCAGTCGAAGCTGCACGTGCATATGATGA

KM388519 TGGCTTGGTTCATTCCCTACCGCAGCCGAAGCTGCACGTGCATATGATGA

KM388520 TGGCTTGGTTCATTCCCTACCGCAGTCGAAGCTGCACGTGCATATGATGA

KJ534637 TGGCTTGGTTCATTCCCTACCGCAGTCGAAGCTGCACGTGCATATGATGA

KM388521 TGGCTTGGTTCATTCCCTACAGCAGTCGAAGCTGCACGTGCATATGATGA

XM_003569037 TGGCTTGGCTCATTCCCTACTGCGGTGGAAGCTGCGCATGCATATGACGA

HQ647359 TGCCGCAAGGGCAATGTATGGCGCCACAGCGCGTGTCAACTTCCCAGAGC

AK376344 TGCCGCAAGGGCAATGTATGGCGCCACAGCGCGTGTCAACTTCCCAGAGC

DQ012941 TGCCGCAAGGGCAATGTATGGCGCCACAGCGCGTGTCAACTTCCCAGAGC

KJ699390 TGCCGCAAGGGCAATGTATGGCGCCACAGCGCGTGTCAACTTCCCAGAGC

AY728807 TGCCGCAAGGGCAATGTATGGCGCCACAGCGCGCGTCAACTTCCCAGAGC

JN107537 TGCCGCAAGGGCAATGTATGGCGCCACAGCGCGCGTCAACTTCCCAGAGC

JQ693159 TGCCGCAAGGGCAATGTATGGCGCCACAGCGCGCGTCAACTTCCCAGAGC

PI428208U TGCGGCAAGGGCAATGTATGGCGCCAAAGCACGTGTCAACTTCTCAGAGC

PI487236S TGCGGCAAGGGCAATGTATGGCGCCAAAGCACGTGTCAACTTCTCAGAGC

PI662241U TGCGGCAAGGGCAATGTATGGCGCCAAAGCACGTGTCAACTTCTCAGAGC

PI428323U TGCGGCAAGGGCAATGTATGGCGCCAAAGCACGTGTCAACTTCTCAGAGC

PI538726U TGCGGCAAGGGCAATGTATGGCGCCAAAGCACGTGTCAACTTCTCAGAGC

PI428231U TGCGGCAAGGGCAATGTATGGCGCCAAAGCACGTGTCAACTTCTCAGAGC

PI554297S TGCGGCAAGGGCAATGTATGGCGCCAAAGCACGTGTCAACTTCTCAGAGC

PI355519M TGCGGCAAGGGCAATGTATGGCGCCAAAGCACGTGTCAACTTCTCAGAGC

PI487235S TGCGGCAAGGGCAATGTATGGCGCCAAAGCACGTGTCAACTTCTCAGAGC

PI554323 TGCGGCAAGGGCAATGTATGGCGCCAAAGCACGTGTCAACTTCTCAGAGC

PI508260 TGCGGCAAGGGCAATGTATGGCGCCAAAGCACGTGTCAACTTCTCAGAGC

PI487268U TGCGGCAAGGGCAATGTATGGCGCCAAAGCACGTGTCAACTTCTCAGAGC

PI662238U TGCGGCAAGGGCAATGTATGGCGCCAAAGCACGTGTCAACTTCTCAGAGC

PI369608S TGCGGCAAGGGCAATGTATGGCGCCAAAGCACGTGTCAACTTCTCAGAGC

PI486263S TGCGGCAAGGGCAATGTATGGCGCCAAAGCACGTGTCAACTTCTCAGAGC

PI538728U TGCGGCAAGGGCAATGTATGGCGCCAAAGCACGTGTCAACTTCTCAGAGC

PI428241U TGCGGCAAGGGCAATGTATGGCGCCAAAGCACGTGTCAACTTCTCAGAGC

PI428237U TGCGGCAAGGGCAATGTATGGCGCCAAAGCACGTGTCAACTTCTCAGAGC

PI219867S TGCGGCAAGGGCAATGTATGGCGCCAAAGCACGTGTCAACTTCTCAGAGC

PI170204S TGCGGCAAGGGCAATGTATGGCGCCAAAGCACGTGTCAACTTCTCAGAGC

PI418582M TGCGGCAAGGGCAATGTATGGCGCCAAAGCACGTGTCAACTTCTCAGAGC

PI428287U TGCGGCAAGGGCAATGTATGGCGCCAAAGCACGTGTCAACTTCTCAGAGC

PI603255 TGCGGCAAGGGCAATGTATGGCGCCAAAGCACGTGTCAACTTCTCAGAGC

PI452130 TGCGGCAAGGGCAATGTATGGCGCCAAAGCACGTGTCAACTTCTCAGAGC

PI428215U TGCGGCAAGGGCAATGTATGGCGCCAAAGCACGTGTCAACTTCTCAGAGC

PI428180U TGCGGCAAGGGCAATGTATGGCGCCAAAGCACGTGTCAACTTCTCAGAGC

PI662264U TGCGGCAAGGGCAATGTATGGCGCCAAAGCACGTGTCAACTTCTCAGAGC

PI220642 TGCGGCAAGGGCAATGTATGGCGCCAAAGCACGTGTCAACTTCTCAGAGC

PI317392 TGCGGCAAGGGCAATGTATGGCGCCAAAGCACGTGTCAACTTCTCAGAGC

DQ195070 TGCGGCAAGGGCAATGTATGGCGCCAAAGCACGTGTCAACTTCTCAGAGC

DQ022952 TGCGGCAAGGGCAATGTATGGCGCCAAAGCACGTGTCAACTTCTCAGAGC

PI662242U TGCGGCAAGGGCAATGTATGGCGCCAAAGCACGTGTCAACTTCTCAGAGC

cltr17668U TGCGGCAAGGGCAATGTATGGCGCCAAAGCACGTGTCAACTTCTCAGAGC

PI662239U TGCGGCAAGGGCAATGTATGGCGCCAAAGCACGTGTCAACTTCTCAGAGC

PI487267U TGCGGCAAGGGCAATGTATGGCGCCAAAGCACGTGTCAACTTCTCAGAGC

PI352486M TGCGGCAAGGGCAATGTATGGCGCCAAAGCACGTGTCAACTTCTCAGAGC

PI277130M TGCGGCAAGGGCAATGTATGGCGCCAAAGCACGTGTCAACTTCTCAGAGC

PI362610M TGCGGCAAGGGCAATGTATGGCGCCAAAGCACGTGTCAACTTCTCAGAGC

PI573452S TGCGGCAAGGGCAATGTATGGCGCCAAAGCACGTGTCAACTTCTCAGAGC

cltr14520M TGCGGCAAGGGCAATGTATGGCGCCAAAGCACGCGTCAACTTCTCAGAGC

PI190946M TGCGGCAAGGGCAATGTATGGCGCCAAAGCACGCGTCAACTTCTCAGAGC

PI191383M TGCGGCAAGGGCAATGTATGGCGCCAAAGCACGCGTCAACTTCTCAGAGC

PI307984M TGCGGCAAGGGCAATGTATGGCGCCAAAGCACGCGTCAACTTCTCAGAGC

PI343181M TGCGGCAAGGGCAATGTATGGCGCCAAAGCACGCGTCAACTTCTCAGAGC

PI190915M TGCGGCAAGGGCAATGTATGGCGCCAAAGCACGCGTCAACTTCTCAGAGC

PI225164M TGCGGCAAGGGCAATGTATGGCGCCAAAGCACGCGTCAACTTCTCAGAGC

PI237659M TGCGGCAAGGGCAATGTATGGCGCCAAAGCACGCGTCAACTTCTCAGAGC

PI265008M TGCGGCAAGGGCAATGTATGGCGCCAAAGCACGCGTCAACTTCTCAGAGC

PI286068M TGCGGCAAGGGCAATGTATGGCGCCAAAGCACGCGTCAACTTCTCAGAGC

PI306543M TGCGGCAAGGGCAATGTATGGCGCCAAAGCACGCGTCAACTTCTCAGAGC

PI326317M TGCGGCAAGGGCAATGTATGGCGCCAAAGCACGCGTCAACTTCTCAGAGC

PI393493S TGCGGCAAGGGCAATGTATGGCGCCAAAGCACGCGTCAACTTCTCAGAGC

PI393496M TGCGGCAAGGGCAATGTATGGCGCCAAAGCACGCGTCAACTTCTCAGAGC

PI427927M TGCGGCAAGGGCAATGTATGGCGCCAAAGCACGCGTCAACTTCTCAGAGC

PI486275 TGCGGCAAGGGCAATGTATGGCGCCAAAGCACGCGTCAACTTCTCAGAGC

PI511379 TGCGGCAAGGGCAATGTATGGCGCCAAAGCACGCGTCAACTTCTCAGAGC

PI10474M TGCGGCAAGGGCAATGTATGGCGCCAAAGCACGCGTCAACTTCTCAGAGC

PI452131 TGCGGCAAGGGCAATGTATGGCGCCAAAGCACGCGTCAACTTCTCAGAGC

PI560720M TGCGGCAAGGGCAATGTATGGCGCCAAAGCACGCGTCAACTTCTCAGAGC

PI573450S TGCGGCAAGGGCAATGTATGGCGCCAAAGCACGTGTCAACTTCTCAGAGC

PI94740M TGCGGCAAGGGCAATGTATGGCGCCAAAGCACGTGTCAACTTCTCAGAGC

PI428183U TGCGGCAAGGGCAATGTATGGCGCCAAAGCACGCGTCAACTTCTCAGAGC

PI538727U TGCGGCAAGGGCAATGTATGGCGCCAAAGCACGCGTCAACTTCTCAGAGC

PI554324 TGCGGCAAGGGCAATGTATGGCGCCAAAGCACGTGTCAACTTCTCAGAGC

PI369602S TGCGGCAAGGGCAATGTATGGCGCCAAAGCACGTGTCAACTTCTCAGAGC

PI487237S TGCGGCAAGGGCAATGTATGGCGCCAAAGCACGTGTCAACTTCTCAGAGC

PI272561M TGCGGCAAGGGCAATGTATGGCGCCAAAGCACGTGTCAACTTCTCAGAGC

PI554320 TGCGGCAAGGGCAATGTATGGCGCCAAAGCACGTGTCAACTTCTCAGAGC

PI603230 TGCGGCAAGGGCAATGTATGGCGCCAAAGCACGTGTCAACTTCTCAGAGC

PI168804M TGCGGCAAGGGCAATGTATGGCGCCAAAGCACGTGTCAACTTCTCAGAGC

DQ195068 TGCGGCAAGGGCAATGTATGGCGCCAAAGCACGTGTCAACTTCTCAGAGC

AF303376 TGCGGCAAGGGCAATGTATGGCGCCAAAGCACGTGTCAACTTCTCAGAGC

DQ022953 TGCGGCAAGGGCAATGTATGGCGCCAAAGCACGTGTCAACTTCTCAGAGC

FR719742 TGCGGCAAGGGCAATGTATGGCGCCAAAGCACGTGTCAACTTCTCAGAGC

KM388515 TGCGGCAAGGGCAATGTATGGTGCCAAAGCACGTGTCAACTTCTCAGAGC

KM388516 TGCGGCAAGGGCAATGTATGGTGCCAAAGCACGTGTCAACTTCTCAGAGC

KM388514 TGCGGCAAGGGCAATGTATGGTGCCAAAGCACGTGTCAACTTCTCAGAGC

HM746657 TGCGGCAAGGGCAATGTATGGCGCCAAAGCACGTGTCAACTTCTCAGAGC

PI486264S TGCGGCAAGGGCAATGTATGGCGCCAAAGCACGTGTCAACTTCTCAGAGC

KM388518 TGCGGCAAGGGCAATGTATGGCGCCAAAGCACGTGTCAACTTCTCAGAGC

KM388517 TGCGGCAAGGGCAATGTATGGCGCCAAAGCACGTGTCAACTTCTCAGAGC

KM388519 TGCGGCAAGGGCAATGTATGGCGCCAAAGCACGTGTCAACTTCTCAGAGC

KM388520 TGCGGCAAGGGCAATGTATGGCGCCAAAGCACGTGTCAACTTCTCAGAGC

KJ534637 TGCGGCAAGGGCAATGTATGGCGCCAAAGCACGTGTCAACTTCTCAGAGC

KM388521 TGCGGCAAGGGCAATGTATGGCGCCAAAGCACGTGTCAATTTCTCTGAGC

XM_003569037 GGCGGCAAGGGCAATGTATGGCGCCAAAGCACGTGTCAACTTCTCAGAGC

HQ647359 ATTCCCCAGATGCCAACTCTGGTTGCACGATGGCACCTTCACTGCTGACG

AK376344 ATTCCCCAGATGCCAACTCTGGTTGCACGATGGCACCTTCACTGCTGACG

DQ012941 ATTCCCCAGATGCCAACTCTGGTTGCACGATGGCACCTTCACTGCTGACG

KJ699390 ATTCCCCAGATGCCAACTCTGGTTGCACGATGGCACCTTCACTGCTGACG

AY728807 ATTCCCCAGATGCCAACTCTGGTTGCACGATGGCACCTTCACTGCTGTTG

JN107537 ATTCCCCAGATGCCAACTCTGGTTGCACGATGGCACCTTCACTGCTGATG

JQ693159 ATTCCCCAGATGCCAACTCTGGTTGCACGATGGCACCTTCACTGCTGATG

PI428208U AGTCCCCGGATGCCAACTCTGGTTGCACGCTGGCACCTCCATTGCTGACG

PI487236S AGTCCCCGGATGCCAACTCTGGTTGCACGCTGGCACCTCCATTGCTGACG

PI662241U AGTCCCCGGATGCCAACTCTGGTTGCACGCTGGCACCTCCATTGCTGACG

PI428323U AGTCCCCGGATGCCAACTCTGGTTGCACGCTGGCACCTCCATTGCTGACG

PI538726U AGTCCCCGGATGCCAACTCTGGTTGCACGCTGGCACCTCCATTGCTGACG

PI428231U AGTCCCCGGATGCCAACTCTGGTTGCACGCTGGCACCTCCATTGCTGACG

PI554297S AGTCCCCGGATGCCAACTCTGGTTGCACGCTGGCACCTCCATTGCTGACG

PI355519M AGTCCCCGGATGCCAACTCTGGTTGCACGCTGGCACCTCCATTGCTGACG

PI487235S AGTCCCCGGATGCCAACTCTGGTTGCACGCTGGCACCTCCATTGCTGACG

PI554323 AGTCCCCGGATGCCAACTCTGGTTGCACGCTGGCACCTCCATTGCTGACG

PI508260 AGTCCCCGGATGCCAACTCTGGTTGCACGCTGGCACCTCCATTGCTGACG

PI487268U AGTCCCCGGATGCCAACTCTGGTTGCACGCTGGCACCTCCATTGCTGACG

PI662238U AGTCCCCGGATGCCAACTCTGGTTGCACGCTGGCACCTCCATTGCTGACG

PI369608S AGTCCCCGGATGCCAACTCTGGTTGCACGCTGGCACCTCCATTGCTGACG

PI486263S AGTCCCCGGATGCCAACTCTGGTTGCACGCTGGCACCTCCATTGCTGACG

PI538728U AGTCCCCGGATGCCAACTCTGGTTGCACGCTGGCACCTCCATTGCTGACG

PI428241U AGTCCCCGGATGCCAACTCTGGTTGCACGCTGGCACCTCCATTGCTGACG

PI428237U AGTCCCCGGATGCCAACTCTGGTTGCACGCTGGCACCTCCATTGCTGACG

PI219867S AGTCCCCGGATGCCAACTCTGGTTGCACGCTGGCACCTCCATTGCTGACG

PI170204S AGTCCCCGGATGCCAACTCTGGTTGCACGCTGGCACCTCCATTGCTGACG

PI418582M AGTCCCCGGATGCCAACTCTGGTTGCACGCTGGCACCTCCATTGCTGACG

PI428287U AGTCCCCGGATGCCAACTCTGGTTGCACGCTGGCACCTCCATTGCTGACG

PI603255 AGTCCCCGGATGCCAACTCTGGTTGCACGCTGGCACCTCCATTGCTGACG

PI452130 AGTCCCCGGATGCCAACTCTGGTTGCACGCTGGCACCTCCATTGCTGACG

PI428215U AGTCCCCGGATGCCAACTCTGGTTGCACGCTGGCACCTCCATTGCTGACG

PI428180U AGTCCCCGGATGCCAACTCTGGTTGCACGCTGGCACCTCCATTGCTGACG

PI662264U AGTCCCCGGATGCCAACTCTGGTTGCACGCTGGCACCTCCATTGCTGACG

PI220642 AGTCCCCGGATGCCAACTCTGGTTGCACGCTGGCACCTCCATTGCTGACG

PI317392 AGTCCCCGGATGCCAACTCTGGTTGCACGCTGGCACCTCCATTGCTGACG

DQ195070 AGTCCCCGGATGCCAACTCTGGTTGCACGCTGGCACCTCCATTGCTGACG

DQ022952 AGTCCCCGGATGCCAACTCTGGTTGCACGCTGGCACCTCCATTGCTGACG

PI662242U AGTCCCCGGATGCCAACTCTGGTTGCACGCTGGCACCTCCATTGCTGACG

cltr17668U AGTCCCCGGATGCCAACTCTGGTTGCACGCTGGCACCTCCATTGCTGACG

PI662239U AGTCCCCGGATGCCAACTCTGGTTGCACGCTGGCACCTCCATTGCTGACG

PI487267U AGTCCCCGGATGCCAACTCTGGTTGCACGCTGGCACCTCCATTGCTGACG

PI352486M AGTCCCCGGATGCCAACTCTGGTTGCACGCTGGCACCTCCATTGCTGACG

PI277130M AGTCCCCGGATGCCAACTCTGGTTGCACGCTGGCACCTCCATTGCTGACG

PI362610M AGTCCCCGGATGCCAACTCTGGTTGCACGCTGGCACCTCCATTGCTGACG

PI573452S AGTCCCCGGATGCCAACTCTGGTTGCACGCTGGCACCTCCATTGCTGACG

cltr14520M AGTCCCCGGATGCCAACTCTGGTTGCACGCTGGCACCTCCATTGCTGACG

PI190946M AGTCCCCGGATGCCAACTCTGGTTGCACGCTGGCACCTCCATTGCTGACG

PI191383M AGTCCCCGGATGCCAACTCTGGTTGCACGCTGGCACCTCCATTGCTGACG

PI307984M AGTCCCCGGATGCCAACTCTGGTTGCACGCTGGCACCTCCATTGCTGACG

PI343181M AGTCCCCGGATGCCAACTCTGGTTGCACGCTGGCACCTCCATTGCTGACG

PI190915M AGTCCCCGGATGCCAACTCTGGTTGCACGCTGGCACCTCCATTGCTGACG

PI225164M AGTCCCCGGATGCCAACTCTGGTTGCACGCTGGCACCTCCATTGCTGACG

PI237659M AGTCCCCGGATGCCAACTCTGGTTGCACGCTGGCACCTCCATTGCTGACG

PI265008M AGTCCCCGGATGCCAACTCTGGTTGCACGCTGGCACCTCCATTGCTGACG

PI286068M AGTCCCCGGATGCCAACTCTGGTTGCACGCTGGCACCTCCATTGCTGACG

PI306543M AGTCCCCGGATGCCAACTCTGGTTGCACGCTGGCACCTCCATTGCTGACG

PI326317M AGTCCCCGGATGCCAACTCTGGTTGCACGCTGGCACCTCCATTGCTGACG

PI393493S AGTCCCCGGATGCCAACTCTGGTTGCACGCTGGCACCTCCATTGCTGACG

PI393496M AGTCCCCGGATGCCAACTCTGGTTGCACGCTGGCACCTCCATTGCTGACG

PI427927M AGTCCCCGGATGCCAACTCTGGTTGCACGCTGGCACCTCCATTGCTGACG

PI486275 AGTCCCCGGATGCCAACTCTGGTTGCACGCTGGCACCTCCATTGCTGACG

PI511379 AGTCCCCGGATGCCAACTCTGGTTGCACGCTGGCACCTCCATTGCTGACG

PI10474M AGTCCCCGGATGCCAACTCTGGTTGCACGCTGGCACCTCCATTGCTGACG

PI452131 AGTCCCCGGATGCCAACTCTGGTTGCACGCTGGCACCTCCATTGCTGACG

PI560720M AGTCCCCGGATGCCAACTCTGGTTGCACGCTGGCACCTCCATTGCTGACG

PI573450S AGTCCCCGGATGCCAACTCTGGTTGCACGCTGGCACCTCCATTGCTGACG

PI94740M AGTCCCCGGATGCCAACTCTGGTTGCACGCTGGCACCTCCATTGCTGACG

PI428183U AGTCCCCGGATGCCAACTCTGGTTGCACGCTGGCACCTCCATTGCTGACG

PI538727U AGTCCCCGGATGCCAACTCTGGTTGCACGCTGGCACCTCCATTGCTGACG

PI554324 AGTCCCCGGATGCCAACTCTGGTTGCACGCTGGCACCTCCATTGCTGACG

PI369602S AGTCCCCGGATGCCAACTCTGGTTGCACGCTGGCACCTCCATTGCTGACG

PI487237S AGTCCCCGGATGCCAACTCTGGTTGCACGCTGGCACCTCCATTGCTGACG

PI272561M AGTCCCCGGATGCCAACTCTGGTTGCACGCTGGCACCTCCATTGCTGACG

PI554320 AGTCCCCGGATGCCAACTCTGGTTGCACGCTGGCACCTCCATTGCTGACG

PI603230 AGTCCCCGGATGCCAACTCTGGTTGCACGCTGGCACCTCCATTGCTGACG

PI168804M AGTCCCCGGATGCCAACTCTGGTTGCACGCTGGCACCTCCATTGCTGATG

DQ195068 AGTCCCCGGATGCCAACTCTGGTTGCACGCTGGCACCTCCATTGCCGATG

AF303376 AGTCCCCGGATGCCAACTCTGGTTGCACGCTGGCACCTCCATTGCCGATG

DQ022953 AGTCCCCGGATGCCAACTCTGGTTGCACGCTGGCACCTCCATTGCCGATG

FR719742 AGTCCCCGGATGCCAACTCTGGTTGCACGCTGGCACCTCCATTGCCGATG

KM388515 AGTCCCCAGATGCCAACTCTGGTTGCACGCTGGCACCTCCATTGCCGATG

KM388516 AGTCCCCAGATGCCAACTCTGGTTGCACGCTGGCACCTCCATTGCCGATG

KM388514 AGTCCCCAGATGCCAACTCTGGTTGCACGCTGGCACCTCCATTGCCGATG

HM746657 AGTCCCCAGATGCCAGCTCTGGTTGCACGCTGGCACCTCCATTGCTGCTG

PI486264S AGTCCCCAGATGCCAGCTCTGGTTGCACGCTGGCACCTCCATTGCTGATG

KM388518 AGTCCCCAGATGCCAATTCTGGTTGCACGCTGGCACCTCCATTGCTGATG

KM388517 AGTCCCCAGATGCCAATTCTGGTTGCACGCTGGCACCTCCATTGCTGATG

KM388519 AGTCCCCAGATGCCAATTCTGGTTGCACGCTGGCACCTCCATTGCTGATG

KM388520 AGTCCCCAGATGCCAATTCTGGTTGCACGCTGGCACCTCCATTGCTGATG

KJ534637 AGTCCCCAGATGCCAACTCTGGTTGCACGCTGGCATCTCCATTGCTGATG

KM388521 AGTCCCCAGATGCCAACTCTGGTTGCACGCTGGCACCTCCAGTGCTGATG

XM_003569037 ATTCCACGGATGCCAACTCTGGTTGCACCTCAGCACCTTCATTGCTGATG

HQ647359 TCTAATGGGGCAACCGCTGTGTCACATCCGTCTGATGGGAAGGATGAATC

AK376344 TCTAATGGGGCAACCGCTGTGTCACATCCGTCTGATGGGAAGGATGAATC

DQ012941 TCTAATGGGGCAACCGCTGTGTCACATCCGTCTGATGGGAAGGATGAATC

KJ699390 TCTAATGGGGCAACCGCTGTGTCACATCCGTCTGATGGGAAGGATGAATC

AY728807 TCTAATGGGGCAACCGCTGTGTCACATCCGTCTGATGGGAAGGATGAATC

JN107537 TCTAATGGGGCAACCGCTGTGTCACATCCGTCTGATGGGAAGGATGAATC

JQ693159 TCTAATGGGGCAACCGCTGTGTCACATCCGTCTGATGGGAAGGATGAATC

PI428208U TCTAATGGGGCAACCGCTGCGTCACATCCTTCTGATGGGAAGGATGAATC

PI487236S TCTAATGGGGCAACCGCTGCGTCACATCCTTCTGATGGGAAGGATGAATC

PI662241U TCTAATGGGGCAACCGCTGCGTCACATCCTTCTGATGGGAAGGATGAATC

PI428323U TCTAATGGGGCAACCGCTGCGTCACATCCTTCTGATGGGAAGGATGAATC

PI538726U TCTAATGGGGCAACCGCTGCGTCACATCCTTCTGATGGGAAGGATGAATC

PI428231U TCTAATGGGGCAACCGCTGCGTCACATCCTTCTGATGGGAAGGATGAATC

PI554297S TCTAATGGGGCAACCGCTGCGTCACATCCTTCTGATGGGAAGGATGAATC

PI355519M TCTAATGGGGCAACCGCTGCGTCACATCCTTCTGATGGGAAGGATGAATC

PI487235S TCTAATGGGGCAACCGCTGCGTCACATCCTTCTGATGGGAAGGATGAATC

PI554323 TCTAATGGGGCAACCGCTGCGTCACATCCTTCTGATGGGAAGGATGAATC

PI508260 TCTAATGGGGCAACCGCTGCGTCACATCCTTCTGATGGGAAGGATGAATC

PI487268U TCTAATGGGGCAACCGCTGCGTCACATCCTTCTGATGGGAAGGATGAATC

PI662238U TCTAATGGGGCAACCGCTGCGTCACATCCTTCTGATGGGAAGGATGAATC

PI369608S TCTAATGGGGCAACCGCTGCGTCACATCCTTCTGATGGGAAGGATGAATC

PI486263S TCTAATGGGGCAACCGCTGCGTCACATCCTTCTGATGGGAAGGATGAATC

PI538728U TCTAATGGGGCAACCGCTGCGTCACATCCTTCTGATGGGAAGGATGAATC

PI428241U TCTAATGGGGCAACCGCTGCGTCACATCCTTCTGATGGGAAGGATGAATC

PI428237U TCTAATGGGGCAACCGCTGCGTCACATCCTTCTGATGGGAAGGATGAATC

PI219867S TCTAATGGGGCAACCGCTGCGTCACATCCTTCTGATGGGAAGGATGAATC

PI170204S TCTAATGGGGCAACCGCTGCGTCACATCCTTCTGATGGGAAGGATGAATC

PI418582M TCTAATGGGGCAACCGCTGCGTCACATCCTTCTGATGGGAAGGATGAATC

PI428287U TCTAATGGGGCAACCGCTGCGTCACATCCTTCTGATGGGAAGGATGAATC

PI603255 TCTAATGGGGCAACCGCTGCGTCACATCCTTCTGATGGGAAGGATGAATC

PI452130 TCTAATGGGGCAACCGCTGCGTCACATCCTTCTGATGGGAAGGATGAATC

PI428215U TCTAATGGGGCAACCGCTGCGTCACATCCTTCTGATGGGAAGGATGAATC

PI428180U TCTAATGGGGCAACCGCTGCGTCACATCCTTCTGATGGGAAGGATGAATC

PI662264U TCTAATGGGGCAACCGCTGCGTCACATCCTTCTGATGGGAAGGATGAATC

PI220642 TCTAATGGGGCAACCGCTGCGTCACATCCTTCTGATGGGAAGGATGAATC

PI317392 TCTAATGGGGCAACCGCTGCGTCACATCCTTCTGATGGGAAGGATGAATC

DQ195070 TCTAATGGGGCAACCGCTGCATCACATCCTTCTGATGGGAAGGATGAATC

DQ022952 TCTAATGGGGCAACCGCTGCATCACATCCTTCTGATGGGAAGGATGAATC

PI662242U TCTAATGGGGCAACCGCTGCGTCACATCCTTCTGATGGGAAGGATGAATC

cltr17668U TCTAATGGGGCAACCGCTGCGTCACATCCTTCTGATGGGAAGGATGAATC

PI662239U TCTAATGGGGCAACCGCTGCGTCACATCCTTCTGATGGGAAGGATGAATC

PI487267U TCTAATGGGGCAACCGCTGCGTCACATCCTTCTGATGGGAAGGATGAATC

PI352486M TCTAATGGGGCAACCGCTGCGTCACATCCTTCTGATGGGAAGGATGAATC

PI277130M TCTAATGGGGCAACCGCTGCGTCACATCCTTCTGATGGGAAGGATGAATC

PI362610M TCTAATGGGGCAACCGCTGCGTCACATCCTTCTGATGGGAAGGATGAATC

PI573452S TCTAATGGGGCAACCGCTGCGTCACATCCTTCTGATGGGAAGGATGAATC

cltr14520M TCTAATGGGGCAACCGCTGCGTCACATCCTTCTGATGGGAAGGATGAATC

PI190946M TCTAATGGGGCAACCGCTGCGTCACATCCTTCTGATGGGAAGGATGAATC

PI191383M TCTAATGGGGCAACCGCTGCGTCACATCCTTCTGATGGGAAGGATGAATC

PI307984M TCTAATGGGGCAACCGCTGCGTCACATCCTTCTGATGGGAAGGATGAATC

PI343181M TCTAATGGGGCAACCGCTGCGTCACATCCTTCTGATGGGAAGGATGAATC

PI190915M TCTAATGGGGCAACCGCTGCGTCACATCCTTCTGATGGGAAGGATGAATC

PI225164M TCTAATGGGGCAACCGCTGCGTCACATCCTTCTGATGGGAAGGATGAATC

PI237659M TCTAATGGGGCAACCGCTGCGTCACATCCTTCTGATGGGAAGGATGAATC

PI265008M TCTAATGGGGCAACCGCTGCGTCACATCCTTCTGATGGGAAGGATGAATC

PI286068M TCTAATGGGGCAACCGCTGCGTCACATCCTTCTGATGGGAAGGATGAATC

PI306543M TCTAATGGGGCAACCGCTGCGTCACATCCTTCTGATGGGAAGGATGAATC

PI326317M TCTAATGGGGCAACCGCTGCGTCACATCCTTCTGATGGGAAGGATGAATC

PI393493S TCTAATGGGGCAACCGCTGCGTCACATCCTTCTGATGGGAAGGATGAATC

PI393496M TCTAATGGGGCAACCGCTGCGTCACATCCTTCTGATGGGAAGGATGAATC

PI427927M TCTAATGGGGCAACCGCTGCGTCACATCCTTCTGATGGGAAGGATGAATC

PI486275 TCTAATGGGGCAACCGCTGCGTCACATCCTTCTGATGGGAAGGATGAATC

PI511379 TCTAATGGGGCAACCGCTGCGTCACATCCTTCTGATGGGAAGGATGAATC

PI10474M TCTAATGGGGCAACCGCTGCGTCACATCCTTCTGATGGGAAGGATGAATC

PI452131 TCTAATGGGGCAACCGCTGCGTCACATCCTTCTGATGGGAAGGATGAATC

PI560720M TCTAATGGGGCAACCGCTGCGTCACATCCTTCTGATGGGAAGGATGAATC

PI573450S TCTAATGGGGCAACCGCTGCGTCACATCCTTCTGATGGGAAGGATGAATC

PI94740M TCTAATGGGGCAACCGCTGCGTCACATCCTTCTGATGGGAAGGATGAATC

PI428183U TCTAATGGGGCAACCGCTGCGTCACATCCTTCTGATGGGAAGGATGAATC

PI538727U TCTAATGGGGCAACCGCTGCGTCACATCCTTCTGATGGGAAGGATGAATC

PI554324 TCTAATGGGGCAACCGCTGCGTCACATCCTTCTGATGGGAAGGATGAATC

PI369602S TCTAATGGGGCAACCGCTGCGTCACATCCTTCTGATGGGAAGGATGAATC

PI487237S TCTAATGGGGCAACCGCTGCGTCACATCCTTCTGATGGGAAGGATGAATC

PI272561M TCTAATGGGGCAACCGCTGCGTCACATCCTTCTGATGGGAAGGATGAATC

PI554320 TCTAATGGGGCAACCGCTGCGTCACATCCTTCTGATGGGAAGGATGAATC

PI603230 TCTAATGGGGCAACCGCTGCGTCACATCCTTCTGATGGGAAGGATGAATC

PI168804M TCTAATGGGGCAACCGCTGCGTCACATCCTTCTGATGGGAAGGATGAATC

DQ195068 TCTAATGGGGCAACCGCTGCGTCACATCCTTCTGATGGGAAGGATGAATC

AF303376 TCTAATGGGGCAACCGCTGCGTCACATCCTTCTGATGGGAAGGATGAATC

DQ022953 TCTAATGGGGCAACCGCTGCGTCACATCCTTCTGATGGGAAGGATGAATC

FR719742 TCTAATGGGGCAACCGCTGCGTCACATCCTTCTGATGGGAAGGATGAATC

KM388515 TCTAATGGGGCAACCGCTGTGTCACATCCTTCTGATGGGAAGGATGAATC

KM388516 TCTAATGGGGCAACCGCTGTGTCACATCCTTCTGATGGGAAGGATGAATC

KM388514 TCTAATGGGGCAACCGCTGTGTCACATCCTTCTGATGGGAAGGATGAATC

HM746657 TCTAATGGGGCAACCGCCGCGTCACATCCTTCTGATGGGAAGGATGAATC

PI486264S TCTAATGGGGCAACTGCCGCATCACATCCTTCTGATGGG-----------

KM388518 TCTAATGGGGCAACCGCTGCGTCACATCCTTCTGATGGGAAGGATGAATC

KM388517 TCTAATGGGGCAACCGCTGCGTCACATCCTTCTGATGGGAAGGATGAATC

KM388519 TCTAATGGGGCAACCGCTGCGTCACATCCTTCTGATGGGAAGGATGAATC

KM388520 TCTAATGGGGCAACTGCTGCGTCACATCCTTCTGATGGGAAGGATGAATC

KJ534637 TCTAATGGGGCAACCTCTGCGTCACATCCTTCTGATGGGAAGGATGAATC

KM388521 TCTAATGGGGCAACCGCTGCGTCACATCCTTCTGATGGGAAGGATGAATC

XM_003569037 TCTAATGGGCCAACCACTGCGTCACATCCATCTGATGAGAAGGATGAGTT

HQ647359 AGAAT---CTCCTCCTTCTCTTGTCTCAAATGCGCCGACAGCTGCGCTGC

AK376344 AGAAT---CTCCTCCTTCTCTTGTCTCAAATGCGCCGACAGCTGCGCTGC

DQ012941 AGAAT---CTCCTCCTTCTCTTGTCTCAAATGCGCCGACAGCTGCGCTGC

KJ699390 AGAAT---CTCCTCCTTCTCTTGTCTCAAATGCGCCAACAGCTGCGCTGC

AY728807 AGAAT---CACCTCCTTCTCTTGTCTCAAATGCGCCGACAGCTGCGCTGC

JN107537 AGAAT---CTCCTCCTTCTCTTGTCTCAAATGCGCCGACAGCTGCGCTGC

JQ693159 AGAAT---CTCCTCCTTCTCTTGTCTCAAATGCGCCGACAGCTGCGCTGC

PI428208U GGAGT---CTCCTCCTTCTCTTATCTCAAATGGGCCGACAGCTGCGCTGC

PI487236S GGAGT---CTCCTCCTTCTCTTATCTCAAATGGGCCGACAGCTGCGCTGC

PI662241U GGAGT---CTCCTCCTTCTCTTATCTCAAATGGGCCGACAGCTGCGCTGC

PI428323U GGAGT---CTCCTCCTTCTCTTATCTCAAATGGGCCGACAGCTGCGCTGC

PI538726U GGAGT---CTCCTCCTTCTCTTATCTCAAATGGGCCGACAGCTGCGCTGC

PI428231U GGAGT---CTCCTCCTTCTCTTATCTCAAATGGGCCGACAGCTGCGCTGC

PI554297S GGAGT---CTCCTCCTTCTCTTATCTCAAATGGGCCGACAGCTGCGCTGC

PI355519M GGAGT---CTCCTCCTTCTCTTATCTCAAATGGGCCGACAGCTGCGCTGC

PI487235S GGAGT---CTCCTCCTTCTCTTATCTCAAATGGGCCGACAGCTGCGCTGC

PI554323 GGAGT---CTCCTCCTTCTCTTATCTCAAATGGGCCGACAGCTGCGCTGC

PI508260 GGAGT---CTCCTCCTTCTCTTATCTCAAATGGGCCGACAGCTGCGCTGC

PI487268U GGAGT---CTCCTCCTTCTCTTATCTCAAATGGGCCGACAGCTGCGCTGC

PI662238U GGAGT---CTCCTCCTTCTCTTATCTCAAATGGGCCGACAGCTGCGCTGC

PI369608S GGAGT---CTCCTCCTTCTCTTATCTCAAATGGGCCGACAGCTGCGCTGC

PI486263S GGAGT---CTCCTCCTTCTCTTATCTCAAATGGGCCGACAGCTGCGCTGC

PI538728U GGAGT---CTCCTCCTTCTCTTATCTCAAATGGGCCGACAGCTGCGCTGC

PI428241U GGAGT---CTCCTCCTTCTCTTATCTCAAATGGGCCGACAGCTGCGCTGC

PI428237U GGAGT---CTCCTCCTTCTCTTATCTCAAATGGGCCGACAGCTGCGCTGC

PI219867S GGAGT---CTCCTCCTTCTCTTATCTCAAATGGGCCGACAGCTGCGCTGC

PI170204S GGAGT---CTCCTCCTTCTCTTATCTCAAATGGGCCGACAGCTGCGCTGC

PI418582M GGAGT---CTCCTCCTTCTCTTATCTCAAATGGGCCGACAGCTGCGCTGC

PI428287U GGAGT---CTCCTCCTTCTCTTATCTCAAATGGGCCGACAGCTGCGCTGC

PI603255 GGAGT---CTCCTCCTTCTCTTATCTCAAATGGGCCGACAGCTGCGCTGC

PI452130 GGAGT---CTCCTCCTTCTCTTATCTCAAATGGGCCGACAGCTGCGCTGC

PI428215U GGAGT---CTCCTCCTTCTCTTATCTCAAATGGGCCGACAGCTGCGCTGC

PI428180U GGAGT---CTCCTCCTTCTCTTATCTCAAATGGGCCGACAGCTGCGCTGC

PI662264U GGAGT---CTCCTCCTTCTCTTATCTCAAATGGGCCGACAGCTGCGCTGC

PI220642 GGAGT---CTCCTCCTTCTCTTATCTCAAATGGGCCGACAGCTGCGCTGC

PI317392 GGAGT---CTCCTCCTTCTCTTATCTCAAATGGGCCGACAGCTGCGCTGC

DQ195070 GGAGT---CTCCTCCTTCTCTTATCTCAAATGGGCCGACAGCTGCGCTGC

DQ022952 GGAGT---CTCCTCCTTCTCTTATCTCAAATGGGCCGACAGCTGCGCTGC

PI662242U GGAGT---CTCCTCCTTCTCTTATCTCAAATGGGCCGACAGCTGCGCTGC

cltr17668U GGAGT---CTCCTCCTTCTCTTATCTCAAATGGGCCGACAGCTGCGCTGC

PI662239U GGAGT---CTCCTCCTTCTCTTATCTCAAATGGGCCGACAGCTGCGCTGC

PI487267U GGAGT---CTCCTCCTTCTCTTATCTCAAATGGGCCGACAGCTGCGCTGC

PI352486M GGAGT---CTCCTCCTTCTCTTATCTCAAATGGGCCGACAGCTGCGCTGC

PI277130M GGAGT---CTCCTCCTTCTCTTATCTCAAATGGGCCGACAGCTGCGCTGC

PI362610M GGAGT---CTCCTCCTTCTCTTATCTCAAATGGGCCGACAGCTGCGCTGC

PI573452S GGAGT---CTCCTCCTTCTCTTATCTCAAATGGGCCGACAGCTGCGCTGC

cltr14520M GGAGT---CTCCTCCTTCTCTTATCTCAAATGGGCCGACAGCTGCGCTGC

PI190946M GGAGT---CTCCTCCTTCTCTTATCTCAAATGGGCCGACAGCTGCGCTGC

PI191383M GGAGT---CTCCTCCTTCTCTTATCTCAAATGGGCCGACAGCTGCGCTGC

PI307984M GGAGT---CTCCTCCTTCTCTTATCTCAAATGGGCCGACAGCTGCGCTGC

PI343181M GGAGT---CTCCTCCTTCTCTTATCTCAAATGGGCCGACAGCTGCGCTGC

PI190915M GGAGT---CTCCTCCTTCTCTTATCTCAAATGGGCCGACAGCTGCGCTGC

PI225164M GGAGT---CTCCTCCTTCTCTTATCTCAAATGGGCCGACAGCTGCGCTGC

PI237659M GGAGT---CTCCTCCTTCTCTTATCTCAAATGGGCCGACAGCTGCGCTGC

PI265008M GGAGT---CTCCTCCTTCTCTTATCTCAAATGGGCCGACAGCTGCGCTGC

PI286068M GGAGT---CTCCTCCTTCTCTTATCTCAAATGGGCCGACAGCTGCGCTGC

PI306543M GGAGT---CTCCTCCTTCTCTTATCTCAAATGGGCCGACAGCTGCGCTGC

PI326317M GGAGT---CTCCTCCTTCTCTTATCTCAAATGGGCCGACAGCTGCGCTGC

PI393493S GGAGT---CTCCTCCTTCTCTTATCTCAAATGGGCCGACAGCTGCGCTGC

PI393496M GGAGT---CTCCTCCTTCTCTTATCTCAAATGGGCCGACAGCTGCGCTGC

PI427927M GGAGT---CTCCTCCTTCTCTTATCTCAAATGGGCCGACAGCTGCGCTGC

PI486275 GGAGT---CTCCTCCTTCTCTTATCTCAAATGGGCCGACAGCTGCGCTGC

PI511379 GGAGT---CTCCTCCTTCTCTTATCTCAAATGGGCCGACAGCTGCGCTGC

PI10474M GGAGT---CTCCTCCTTCTCTTATCTCAAATGGGCCGACAGCTGCGCTGC

PI452131 GGAGT---CTCCTCCTTCTCTTATCTCAAATGGGCCGACAGCTGCGCTGC

PI560720M GGAGT---CTCCTCCTTCTCTTATCTCAAATGGGCCGACAGCTGCGCTGC

PI573450S GGAGT---CTCCTCCTTCTCTTATCTCAAATGGGCCGACAGCTGCGCTGC

PI94740M GGAGT---CTCCTCCTTCTCTTATCTCAAATGGGCCGACAGCTGCGCTGC

PI428183U GGAGT---CTCCTCCTTCTCTTATCTCAAATGGGCCGACAGCTGCGCTGC

PI538727U GGAGT---CTCCTCCTTCTCTTATCTCAAATGGGCCGACAGCTGCGCTGC

PI554324 GGAGT---CTCCTCCTTCTCTTATCTCAAATGGGCCGACAGCTGCGCTGC

PI369602S GGAGT---CTCCTCCTTCTCTTATCTCAAATGGGCCGACAGCTGCGCTGC

PI487237S GGAGT---CTCCTCCTTCTCTTATCTCAAATGGGCCGACAGCTGCGCTGC

PI272561M GGAGT---CTCCTCCTTCTCTTATCTCAAATGGGCCGACAGCTGCGCTGC

PI554320 GGAGT---CTCCTCCTTCTCTTATCTCAAATGGGCCGACAGCTGCGCTGC

PI603230 GGAGT---CTCCTCCTTCTCTTATCTCAAATGGGCCGACAGCTGCGCTGC

PI168804M GGAGT---CTCCTCCTTCTCTTATCTCAAATGGGCCGACAGCTGCGCTGC

DQ195068 GGAGT---CTCCTCCTTCTCTTATCTCAAATGCGCCGACAGCTGCGCTGC

AF303376 GGAGT---CTCCTCCTTCTCTTATCTCAAATGCGCCGACAGCTGCGCTGC

DQ022953 GGAGT---CTCCTCCTTCTCTTATCTCAAATGCGCCGACAGCTGCGCTGC

FR719742 GGAGT---CTCCTCCTTCTCTTATCTCAGATGCGCCGACAGCTCCGCTGC

KM388515 GGGGT---CTCCTCCTTCTCTTATCTCAAATGCGCCGACAGCTGCGCTGC

KM388516 GGGGT---CTCCTCCTTCTCTTATCTCAAATGCGCCGACAGCTGCGCTGC

KM388514 GGGGT---CTCCTCCTTCTCTTATCTCAAATGCGCCGACAGCTGCGCTGC

HM746657 GGAGT---CTCCTCCTTCTCTTATCTCAAATGCGCCGGCAGCTGCGCTGC

PI486264S --------------------------------------------------

KM388518 GGAGTCTCCTCCTCCTCCTCTTATCTCAAATGTGCCGACAGCTGCGCTGC

KM388517 GGAGTCTCCTCCTCCTCCTCTTATCTCAAATGTGCCGACAGCTGCGCTGC

KM388519 GGAGT---CTCCTCCTCCTCTTATCTCAAATGCGCCGACAGCTGCGCTGC

KM388520 GGAGT---CTCCTCCTCCTCTTATCTCAAATGCGCCGACAGCTGCGCTGC

KJ534637 GGAGT---CTCCTCCTTCTCTTATCTCAAATGCGCCGACAGCTGCGC---

KM388521 GGAGT---CTCCTCCTTCTCTTATCTCAAATGCGCCGACAGCTGCGCTGC

XM_003569037 GGAAT---CTCCTCCATTTGTGATGTCCAGTGCGCCGACTGATGGGCTGC

HQ647359 ATCGGTCTGATGCCAAGGATGAGTTTGAGTCTGCAGGGACTGTGGCGCAT

AK376344 ATCGGTCTGATGCCAAGGATGAGTTTGAGTCTGCAGGGACTGTGGCGCAT

DQ012941 ATCGGTCTGATGCCAAGGATGAGTTTGAGTCTGCAGGGACTGTGGCGCAT

KJ699390 ATCGGTCTGATGCCAAGGATGAGTTTGAGTCTGCAGGGACTGTGGCGCAT

AY728807 ATCGGTCTGATGCCAAGGATGAGTTTGAGTCTTCAGGGACTGTGGCGCAT

JN107537 ATCGGTCTGATGCCAAGGATGAGTTTGAGTCTTCAGGGACTGTGGCGCAT

JQ693159 ATCGGTCTGATGCCAAGGATGAGTTTGAGTCTTCAGGGACTGTGGCGCAT

PI428208U ATCGGTCTGATGCTAAGGATGAGTCTGAGTCTGCAGGGACCGTGGCACGT

PI487236S ATCGGTCTGATGCTAAGGATGAGTCTGAGTCTGCAGGGACCGTGGCACGT

PI662241U ATCGGTCTGATGCTAAGGATGAGTCTGAGTCTGCAGGGACCGTGGCACGT

PI428323U ATCGGTCTGATGCTAAGGATGAGTCTGAGTCTGCAGGGACCGTGGCACGT

PI538726U ATCGGTCTGATGCTAAGGATGAGTCTGAGTCTGCAGGGACCGTGGCACGT

PI428231U ATCGGTCTGATGCTAAGGATGAGTCTGAGTCTGCAGGGACCGTGGCACGT

PI554297S ATCGGTCTGATGCTAAGGATGAGTCTGAGTCTGCAGGGACCGTGGCACGT

PI355519M ATCGGTCTGATGCTAAGGATGAGTCTGAGTCTGCAGGGACCGTGGCACGT

PI487235S ATCGGTCTGATGCTAAGGATGAGTCTGAGTCTGCAGGGACCGTGGCACGT

PI554323 ATCGGTCTGATGCTAAGGATGAGTCTGAGTCTGCAGGGACCGTGGCACGT

PI508260 ATCGGTCTGATGCTAAGGATGAGTCTGAGTCTGCAGGGACCGTGGCACGT

PI487268U ATCGGTCTGATGCTAAGGATGAGTCTGAGTCTGCAGGGACCGTGGCACGT

PI662238U ATCGGTCTGATGCTAAGGATGAGTCTGAGTCTGCAGGGACCGTGGCACGT

PI369608S ATCGGTCTGATGCTAAGGATGAGTCTGAGTCTGCAGGGACCGTGGCACGT

PI486263S ATCGGTCTGATGCTAAGGATGAGTCTGAGTCTGCAGGGACCGTGGCACGT

PI538728U ATCGGTCTGATGCTAAGGATGAGTCTGAGTCTGCAGGGACCGTGGCACGT

PI428241U ATCGGTCTGATGCTAAGGATGAGTCTGAGTCTGCAGGGACCGTGGCACGT

PI428237U ATCGGTCTGATGCTAAGGATGAGTCTGAGTCTGCAGGGACCGTGGCACGT

PI219867S ATCGGTCTGATGCTAAGGATGAGTCTGAGTCTGCAGGGACCGTGGCACGT

PI170204S ATCGGTCTGATGCTAAGGATGAGTCTGAGTCTGCAGGGACCGTGGCACGT

PI418582M ATCGGTCTGATGCTAAGGATGAGTCTGAGTCTGCAGGGACCGTGGCACGT

PI428287U ATCGGTCTGATGCTAAGGATGAGTCTGAGTCTGCAGGGACCGTGGCACGT

PI603255 ATCGGTCTGATGCTAAGGATGAGTCTGAGTCTGCAGGGACCGTGGCACGT

PI452130 ATCGGTCTGATGCTAAGGATGAGTCTGAGTCTGCAGGGACCGTGGCACGT

PI428215U ATCGGTCTGATGCTAAGGATGAGTCTGAGTCTGCAGGGACCGTGGCACGT

PI428180U ATCGGTCTGATGCTAAGGATGAGTCTGAGTCTGCAGGGACCGTGGCACGT

PI662264U ATCGGTCTGATGCTAAGGATGAGTCTGAGTCTGCAGGGACCGTGGCACGT

PI220642 ATCGGTCTGATGCTAAGGATGAGTCTGAGTCTGCAGGGACCGTGGCACGT

PI317392 ATCGGTCTGATGCTAAGGATGAGTCTGAGTCTGCAGGGACCGTGGCACGT

DQ195070 GTCGGTCTGATGCTAAGGATGAGTCTGAGTCTGCAGGGACCGTGGCACGT

DQ022952 GTCGGTCTGATGCTAAGGATGAGTCTGAGTCTGCAGGGACCGTGGCACGT

PI662242U ATCGGTCTGATGCTAAGGATGAGTCTGAGTCTGCAGGGACCGTGGCACGT

cltr17668U ATCGGTCTGATGCTAAGGATGAGTCTGAGTCTGCAGGGACCGTGGCACGT

PI662239U ATCGGTCTGATGCTAAGGATGAGTCTGAGTCTGCAGGGACCGTGGCACGT

PI487267U ATCGGTCTGATGCTAAGGATGAGTCTGAGTCTGCAGGGACCGTGGCACGT

PI352486M ATCGGTCTGATGCTAAGGATGAGTCTGAGTCTGCAGGGACCGTGGCACGT

PI277130M ATCGTTCTGATGCTAAGGATGAGTCTGAGTCTGCAGGGACCGTGGCACGT

PI362610M ATCGTTCTGATGCTAAGGATGAGTCTGAGTCTGCAGGGACCGTGGCACGT

PI573452S ATCGTTCTGATGCTAAGGATGAGTCTGAGTCTGCAGGGACCGTGGCACGT

cltr14520M ATCGTTCTGATGCTAAGGATGAGTCTGAGTCTGCAGGGACCGTGGCACGT

PI190946M ATCGTTCTGATGCTAAGGATGAGTCTGAGTCTGCAGGGACCGTGGCACGT

PI191383M ATCGTTCTGATGCTAAGGATGAGTCTGAGTCTGCAGGGACCGTGGCACGT

PI307984M ATCGTTCTGATGCTAAGGATGAGTCTGAGTCTGCAGGGACCGTGGCACGT

PI343181M ATCGTTCTGATGCTAAGGATGAGTCTGAGTCTGCAGGGACCGTGGCACGT

PI190915M ATCGTTCTGATGCTAAGGATGAGTCTGAGTCTGCAGGGACCGTGGCACGT

PI225164M ATCGTTCTGATGCTAAGGATGAGTCTGAGTCTGCAGGGACCGTGGCACGT

PI237659M ATCGTTCTGATGCTAAGGATGAGTCTGAGTCTGCAGGGACCGTGGCACGT

PI265008M ATCGTTCTGATGCTAAGGATGAGTCTGAGTCTGCAGGGACCGTGGCACGT

PI286068M ATCGTTCTGATGCTAAGGATGAGTCTGAGTCTGCAGGGACCGTGGCACGT

PI306543M ATCGTTCTGATGCTAAGGATGAGTCTGAGTCTGCAGGGACCGTGGCACGT

PI326317M ATCGTTCTGATGCTAAGGATGAGTCTGAGTCTGCAGGGACCGTGGCACGT

PI393493S ATCGTTCTGATGCTAAGGATGAGTCTGAGTCTGCAGGGACCGTGGCACGT

PI393496M ATCGTTCTGATGCTAAGGATGAGTCTGAGTCTGCAGGGACCGTGGCACGT

PI427927M ATCGTTCTGATGCTAAGGATGAGTCTGAGTCTGCAGGGACCGTGGCACGT

PI486275 ATCGTTCTGATGCTAAGGATGAGTCTGAGTCTGCAGGGACCGTGGCACGT

PI511379 ATCGTTCTGATGCTAAGGATGAGTCTGAGTCTGCAGGGACCGTGGCACGT

PI10474M ATCGTTCTGATGCTAAGGATGAGTCTGAGTCTGCAGGGACCGTGGCACGT

PI452131 ATCGTTCTGATGCTAAGGATGAGTCTGAGTCTGCAGGGACCGTGGCACGT

PI560720M ATCGTTCTGATGCTAAGGATGAGTCTGAGTCTGCAGGGACCGTGGCACGT

PI573450S ATCGGTCTGATGCTAAGGATGAGTCTGAGTCTGCAGGGACCGTGGCACGT

PI94740M ATCGTTCTGATGCTAAGGATGAGTCTGAGTCTGCAGGGACCGTGGCACGT

PI428183U ATCGGTCTGATGCTAAGGATGAGTCTGAGTCTGCAGGGACCGTGGCACGT

PI538727U ATCGGTCTGATGCTAAGGATGAGTCTGAGTCTGCAGGGACCGTGGCACGT

PI554324 ATCGGTCTGATGCTAAGGATGAGTCTGAGTCTGCAGGGACCGTGGCACGT

PI369602S ATCGGTCTGATGCTAAGGATGAGTCTGAGTCTGCAGGGACCGTGGCACGT

PI487237S ATCGGTCTGATGCTAAGGATGAGTCTGAGTCTGCAGGGACCGTGGCACGT

PI272561M ATCGGTCTGATGCTAAGGATGAGTCTGAGTCTGCAGGGACCGTGGCACGT

PI554320 ATCGGTCTGATGCTAAGGATGAGTCTGAGTCTGCAGGGACCGTGGCACGT

PI603230 ATCGGTCTGATGCTAAGGATGAGTCTGAGTCTGCAGGGACCGTGGCACGT

PI168804M ATCGGTCTGATGCTAAGGATGAGTCTGAGTCTGCAGGGACCGTGGCACGT

DQ195068 ATCGGTCTGATGCTAAGGATGAGTCTGAGTCTGCAGGGACCGTGGCACGT

AF303376 ATCGGTCTGATGCTAAGGATGAGTCTGAGTCTGCAGGGACCGTGGCACGT

DQ022953 ATCGGTCTGATGCTAAGGATGAGTCTGAGTCTGCAGGGACCGTGGCACGT

FR719742 ATCGGTCTGATGCTAAGGGTGAGTCCGAGTCTGCAGGGACCGTGGCACGT

KM388515 ATCGATCTGATGCTAAGGATGAGTCTGAGTCTGCAGGGACCGTCGCACAT

KM388516 ATCGATCTGATGCTAAGGATGAGTCTGAGTCTGCAGGGACCGTCGCACAT

KM388514 ATCGATCTGATGCTAAGGATGAGTCTGAGTCTGCAGGGACCGTCGCACAT

HM746657 ATCGATCTGATGCTAAGGATGAGTCTGAGTCTGCAGGGACCATGGCACAT

PI486264S --------------AAGGATGAGTCTGAGTCTGCAGGGACCGTGGCACAT

KM388518 ATCGGTCTGATGCTATGT------CTGAGTCTGCAGGGACCGTGGCACAT

KM388517 ATCGGTCTGGTGCTATGT------CTGAGTCTGCAGGGACCGTGGCACAT

KM388519 ATCGGTCTGATGCTATGT------CTGAGTCTGCAGGGACCGTGGCACAT

KM388520 ATCGGTCTGATGCTAAGGATGAGTCTGAGTCTGCAGGGACAGTGGCACAT

KJ534637 ---------------A------GTCTGAGTCTGCAGGGACCGTGGCACAT

KM388521 ATCGGTCTGATGCTAAGGATGAGTCTGAGTCTGCAGGGACCATGGCACAT

XM_003569037 ATCAGCCTGATGCGAAGGATGAGTATGGATCTGCAGGGACACTGGTGCAC

HQ647359 AAGGTGAAAACAGAAGTGAGCAATGATTTGGGAAGTACCCATGAGGAGCA

AK376344 AAGGTGAAAACAGAAGTGAGCAATGATTTGGGAAGTACCCATGAGGAGCA

DQ012941 AAGGTGAAAACAGAAGTGAGCAATGATTTGGGAAGTACCCATGAGGAGCA

KJ699390 AAGGTGAAAACAGAAGTGAGCAATGATTTGGGAAGTACCCATGAGGAGCA

AY728807 AAGGTGAAAGCAGAAGTGAGCAATGATTTGAGAAGTACCCATGAGGAGCA

JN107537 AAGGTGAAAACAGAAGTGAGCAATGATTTGAGAAGTACCCATGAGGAGCA

JQ693159 AAGGTGAAAACAGAAGTGAGCAATGATTTGAGAAGTACCCATGAGGAGCA

PI428208U AAGGTGAAGAAAGAAGTGAGCAATGATTTGAGAAGTACCCATGAGGAGCA

PI487236S AAGGTGAAGAAAGAAGTGAGCAATGATTTGAGAAGTACCCATGAGGAGCA

PI662241U AAGGTGAAGAAAGAAGTGAGCAATGATTTGAGAAGTACCCATGAGGAGCA

PI428323U AAGGTGAAGAAAGAAGTGAGCAATGATTTGAGAAGTACCCATGAGGAGCA

PI538726U AAGGTGAAGAAAGAAGTGAGCAATGATTTGAGAAGTACCCATGAGGAGCA

PI428231U AAGGTGAAGAAAGAAGTGAGCAATGATTTGAGAAGTACCCATGAGGAGCA

PI554297S AAGGTGAAGAAAGAAGTGAGCAATGATTTGAGAAGTACCCATGAGGAGCA

PI355519M AAGGTGAAGAAAGAAGTGAGCAATGATTTGAGAAGTACCCATGAGGAGCA

PI487235S AAGGTGAAGAAAGAAGTGAGCAATGATTTGAGAAGTACCCATGAGGAGCA

PI554323 AAGGTGAAGAAAGAAGTGAGCAATGATTTGAGAAGTACCCATGAGGAGCA

PI508260 AAGGTGAAGAAAGAAGTGAGCAATGATTTGAGAAGTACCCATGAGGAGCA

PI487268U AAGGTGAAGAAAGAAGTGAGCAATGATTTGAGAAGTACCCATGAGGAGCA

PI662238U AAGGTGAAGAAAGAAGTGAGCAATGATTTGAGAAGTACCCATGAGGAGCA

PI369608S AAGGTGAAGAAAGAAGTGAGCAATGATTTGAGAAGTACCCATGAGGAGCA

PI486263S AAGGTGAAGAAAGAAGTGAGCAATGATTTGAGAAGTACCCATGAGGAGCA

PI538728U AAGGTGAAGAAAGAAGTGAGCAATGATTTGAGAAGTACCCATGAGGAGCA

PI428241U AAGGTGAAGAAAGAAGTGAGCAATGATTTGAGAAGTACCCATGAGGAGCA

PI428237U AAGGTGAAGAAAGAAGTGAGCAATGATTTGAGAAGTACCCATGAGGAGCA

PI219867S AAGGTGAAGAAAGAAGTGAGCAATGATTTGAGAAGTACCCATGAGGAGCA

PI170204S AAGGTGAAGAAAGAAGTGAGCAATGATTTGAGAAGTACCCATGAGGAGCA

PI418582M AAGGTGAAGAAAGAAGTGAGCAATGATTTGAGAAGTACCCATGAGGAGCA

PI428287U AAGGTGAAGAAAGAAGTGAGCAATGATTTGAGAAGTACCCATGAGGAGCA

PI603255 AAGGTGAAGAAAGAAGTGAGCAATGATTTGAGAAGTACCCATGAGGAGCA

PI452130 AAGGTGAAGAAAGAAGTGAGCAATGATTTGAGAAGTACCCATGAGGAGCA

PI428215U AAGGTGAAGAAAGAAGTGAGCAATGATTTGAGAAGTACCCATGAGGAGCA

PI428180U AAGGTGAAGAAAGAAGTGAGCAATGATTTGAGAAGTACCCATGAGGAGCA

PI662264U AAGGTGAAGAAAGAAGTGAGCAATGATTTGAGAAGTACCCATGAGGAGCA

PI220642 AAGGTGAAGAAAGAAGTGAGCAATGATTTGAGAAGTACCCATGAGGAGCA

PI317392 AAGGTGAAGAAAGAAGTGAGCAATGATTTGAGAAGTACCCATGAGGAGCA

DQ195070 AAGGTGAAGAAAGAAGTGAGCAATGATTTGAGAAGTACCCATGAGGAGCA

DQ022952 AAGGTGAAGAAAGAAGTGAGCAATGATTTGAGAAGTACCCATGAGGAGCA

PI662242U AAGGTGAAGAAAGAAGTGAGCAATGATTTGAGAAGTACCCATGAGGAGCA

cltr17668U AAGGTGAAGAAAGAAGTGAGCAATGATTTGAGAAGTACCCATGAGGAGCA

PI662239U AAGGTGAAGAAAGAAGTGAGCAATGATTTGAGAAGTACCCATGAGGAGCA

PI487267U AAGGTGAAGAAAGAAGTGAGCAATGATTTGAGAAGTACCCATGAGGAGCA

PI352486M AAGGTGAAGAAAGAAGTGAGCAATGATTTGAGAAGTACCCATGAGGAGCA

PI277130M AAGGTGAAGAAAGAAGTGAGCAATGATGTGAGAAGTACCCATGAGGAGCA

PI362610M AAGGTGAAGAAAGAAGTGAGCAATGATGTGAGAAGTACCCATGAGGAGCA

PI573452S AAGGTGAAGAAAGAAGTGAGCAATGATGTGAGAAGTACCCATGAGGAGCA

cltr14520M AAGGTGAAGAAAGAAGTGAGCAATGATGTGAGAAGTACCCATGAGGAGCA

PI190946M AAGGTGAAGAAAGAAGTGAGCAATGATGTGAGAAGTACCCATGAGGAGCA

PI191383M AAGGTGAAGAAAGAAGTGAGCAATGATGTGAGAAGTACCCATGAGGAGCA

PI307984M AAGGTGAAGAAAGAAGTGAGCAATGATGTGAGAAGTACCCATGAGGAGCA

PI343181M AAGGTGAAGAAAGAAGTGAGCAATGATGTGAGAAGTACCCATGAGGAGCA

PI190915M AAGGTGAAGAAAGAAGTGAGCAATGATGTGAGAAGTACCCATGAGGAGCA

PI225164M AAGGTGAAGAAAGAAGTGAGCAATGATGTGAGAAGTACCCATGAGGAGCA

PI237659M AAGGTGAAGAAAGAAGTGAGCAATGATGTGAGAAGTACCCATGAGGAGCA

PI265008M AAGGTGAAGAAAGAAGTGAGCAATGATGTGAGAAGTACCCATGAGGAGCA

PI286068M AAGGTGAAGAAAGAAGTGAGCAATGATGTGAGAAGTACCCATGAGGAGCA

PI306543M AAGGTGAAGAAAGAAGTGAGCAATGATGTGAGAAGTACCCATGAGGAGCA

PI326317M AAGGTGAAGAAAGAAGTGAGCAATGATGTGAGAAGTACCCATGAGGAGCA

PI393493S AAGGTGAAGAAAGAAGTGAGCAATGATGTGAGAAGTACCCATGAGGAGCA

PI393496M AAGGTGAAGAAAGAAGTGAGCAATGATGTGAGAAGTACCCATGAGGAGCA

PI427927M AAGGTGAAGAAAGAAGTGAGCAATGATGTGAGAAGTACCCATGAGGAGCA

PI486275 AAGGTGAAGAAAGAAGTGAGCAATGATGTGAGAAGTACCCATGAGGAGCA

PI511379 AAGGTGAAGAAAGAAGTGAGCAATGATGTGAGAAGTACCCATGAGGAGCA

PI10474M AAGGTGAAGAAAGAAGTGAGCAATGATGTGAGAAGTACCCATGAGGAGCA

PI452131 AAGGTGAAGAAAGAAGTGAGCAATGATGTGAGAAGTACCCATGAGGAGCA

PI560720M AAGGTGAAGAAAGAAGTGAGCAATGATGTGAGAAGTACCCATGAGGAGCA

PI573450S AAGGTGAAGAAAGAAGTGAGCAATGATGTGAGAAGTACCCATGAGGAGCA

PI94740M AAGGTGAAGAAAGAAGTGAGCAATGATTTGAGAAGTACCCATGAGGAGCA

PI428183U AAGGTGAAGAAAGAAGTGAGCAATGATTTGAGAAGTACCCATGAGGAGCA

PI538727U AAGGTGAAGAAAGAAGTGAGCAATGATTTGAGAAGTACCCATGAGGAGCA

PI554324 AAGGTGAAGAAAGAAGTGAGCAATGATTTGAGAAGTACCCATGAGGAGCA

PI369602S AAGGTGAAGAAAGAAGTGAGCAATGATTTGAGAAGTACCCATGAGGAGCA

PI487237S AAGGTGAAGAAAGAAGTGAGCAATGATTTGAGAAGTACCCATGAGGAGCA

PI272561M AAGGTGAAGAAAGAAGTGAGCAATGATTTGAGAAGTACCCATGAGGAGCA

PI554320 AAGGTGAAGAAAGAAGTGAGCAATGATTTGAGAAGTACCCATGAGGAGCA

PI603230 AAGGTGAAAAAAGAAGTGAGCAATGATTTGAGAAGTACCCATGAGGAGCA

PI168804M AAGGTGAAAAAAGAAGTGAGCAATGATTTGAGAAGTACCCATGAGGAGCA

DQ195068 AAGGTGAAAAAAGAAGTGAGCAATGATTTGAGAAGTACCCATGAGGAGCA

AF303376 AAGGTGAAAAAAGAAGTGAGCAATGATTTGAGAAGTACCCATGAGGAGCA

DQ022953 AAGGTGAAAAAAGAAGTGAGCAATGATTTGAGAAGTACCCATGAGGAGCA

FR719742 AAGGTGAAAAAAGAAGTGAGCAATGATTTGAGAAGTACCCATGAGGAGCA

KM388515 AAGGTGAAAAAAGAAGTGAGCAATGATTTGAGAAGTACCCATGAGGGGCA

KM388516 AAGGTGAAAAAAGAAGTGAGCAATGATTTGAGAAGTACCCATGAGGAGCA

KM388514 AAGGTGAAAAAAGAAGTGAGCAATGATTTGAGAAGTACCCATGAGGAGCA

HM746657 AAGGTGAAAAAAGAAGTGAGCAATGATTTGAGAAGTACCCATGAGGAGCA

PI486264S AAGGTGAAAAAAGAAGTGAGCAATGATTTGAGAAGTACCCATGAGGAGCA

KM388518 AAGGTGAAAACAGAAGTGAGCAATGATTTGAGAAGTACCCATGAGGAGCA

KM388517 AAGGTGAAAACAGAAGTGAGCAATGATTTGAGAAGTACCCATGAGGAGCA

KM388519 AAGGTGAAAACAGAAGTGAGCAATGATTTGAGAAGTACCCATGAGGAGCA

KM388520 AAGGTGAAAACAGAAGTAAGCAATGATTTGAGAAGTACCCATGAGGAGCA

KJ534637 AAGGTGAAAACAGAAGTGAGCAATGATTTGAGAAGTACACATGAGGAGCA

KM388521 AAGGTGAAAACAGAAGTGAGCAATGATTTGAGAAGCTCCCATGAGGAGCA

XM_003569037 GAGGTGAAAACGGAAGTGAGCAATGACTTGAGAAGTACCTGTGAGGAGCA

HQ647359 CAAGGCCCTGGAAGTATTCCAACCAAAAGGGAAGGCTTTACATAAAGAAG

AK376344 CAAGGCCCTGGAAGTATTCCAACCAAAAGGGAAGGCTTTACATAAAGAAG

DQ012941 CAAGGCCCTGGAAGTATTCCAACCAAAAGGGAAGGCTTTACATAAAGAAG

KJ699390 CAAGGCCCTGGAAGTATTCCAACCAAAAGGGAAGGCTTTACATAAAGAAG

AY728807 CAAGGCCCTGGAAGTAACCCAACCAAAAGGGAAGGCTTTACATAAAGAAG

JN107537 CAAGGCCCTGGAAGTAACCCAACCAAAAGGGAAGGCTTTACATAAAGAAG

JQ693159 CAAGGCCCTGGAAGTAACCCAACCAAAAGGGAAGGCTTTACATAAAGAAG

PI428208U CAAGACCCTGGAAGTATCCCAACCAAAAGGGAAGGCTTTACATAAAGAAG

PI487236S CAAGACCCTGGAAGTATCCCAACCAAAAGGGAAGGCTTTACATAAAGAAG

PI662241U CAAGACCCTGGAAGTATCCCAACCAAAAGGGAAGGCTTTACATAAAGAAG

PI428323U CAAGACCCTGGAAGTATCCCAACCAAAAGGGAAGGCTTTACATAAAGAAG

PI538726U CAAGACCCTGGAAGTATCCCAACCAAAAGGGAAGGCTTTACATAAAGAAG

PI428231U CAAGACCCTGGAAGTATCCCAACCAAAAGGGAAGGCTTTACATAAAGAAG

PI554297S CAAGACCCTGGAAGTATCCCAACCAAAAGGGAAGGCTTTACATAAAGAAG

PI355519M CAAGACCCTGGAAGTATCCCAACCAAAAGGGAAGGCTTTACATAAAGAAG

PI487235S CAAGACCCTGGAAGTATCCCAACCAAAAGGGAAGGCTTTACATAAAGAAG

PI554323 CAAGACCCTGGAAGTATCCCAACCAAAAGGGAAGGCTTTACATAAAGAAG

PI508260 CAAGACCCTGGAAGTATCCCAACCAAAAGGGAAGGCTTTACATAAAGAAG

PI487268U CAAGACCCTGGAAGTATCCCAACCAAAAGGGAAGGCTTTACATAAAGAAG

PI662238U CAAGACCCTGGAAGTATCCCAACCAAAAGGGAAGGCTTTACATAAAGAAG

PI369608S CAAGACCCTGGAAGTATCCCAACCAAAAGGGAAGGCTTTACATAAAGAAG

PI486263S CAAGACCCTGGAAGTATCCCAACCAAAAGGGAAGGCTTTACATAAAGAAG

PI538728U CAAGACCCTGGAAGTATCCCAACCAAAAGGGAAGGCTTTACATAAAGAAG

PI428241U CAAGACCCTGGAAGTATCCCAACCAAAAGGGAAGGCTTTACATAAAGAAG

PI428237U CAAGACCCTGGAAGTATCCCAACCAAAAGGGAAGGCTTTACATAAAGAAG

PI219867S CAAGACCCTGGAAGTATCCCAACCAAAAGGGAAGGCTTTACATAAAGAAG

PI170204S CAAGACCCTGGAAGTATCCCAACCAAAAGGGAAGGCTTTACATAAAGAAG

PI418582M CAAGACCCTGGAAGTATCCCAACCAAAAGGGAAGGCTTTACATAAAGAAG

PI428287U CAAGACCCTGGAAGTATCCCAACCAAAAGGGAAGGCTTTACATAAAGAAG

PI603255 CAAGACCCTGGAAGTATCCCAACCAAAAGGGAAGGCTTTACATAAAGAAG

PI452130 CAAGACCCTGGAAGTATCCCAACCAAAAGGGAAGGCTTTACATAAAGAAG

PI428215U CAAGACCCTGGAAGTATCCCAACCAAAAGGGAAGGCTTTACATAAAGAAG

PI428180U CAAGACCCTGGAAGTATCCCAACCAAAAGGGAAGGCTTTACATAAAGAAG

PI662264U CAAGACCCTGGAAGTATCCCAACCAAAAGGGAAGGCTTTACATAAAGAAG

PI220642 CAAGACCCTGGAAGTATCCCAACCAAAAGGGAAGGCTTTACATAAAGAAG

PI317392 CAAGACCCTGGAAGTATCCCAACCAAAAGGGAAGGCTTTACATAAAGAAG

DQ195070 CAAGACCCTGGAAGTATCCCAACCAAAAGGGAAGGCTTTACATAAAGAAG

DQ022952 CAAGACCCTGGAAGTATCCCAACCAAAAGGGAAGGCTTTACATAAAGAAG

PI662242U CAAGACCCTGGATGTATCCCAACCAAAAGGGAAGGCTTTACATAAAGAAG

cltr17668U CAAGACCCTGGATGTATCCCAACCAAAAGGGAAGGCTTTACATAAAGAAG

PI662239U CAAGACCCTGGATGTATCCCAACCAAAAGGGAAGGCTTTACATAAAGAAG

PI487267U CAAGACCCTGGATGTATCCCAACCAAAAGGGAAGGCTTTACATAAAGAAG

PI352486M CAAGACCCTGGAAGTATCCCAACCAAAAGGGAAGGCTTTACATAAAGAAG

PI277130M CAAGACCCTGGAAGTATCCCAACCAAAAGGGAAGGCTTTACATAAAGAAG

PI362610M CAAGACCCTGGAAGTATCCCAACCAAAAGGGAAGGCTTTACATAAAGAAG

PI573452S CAAGACCCTGGAAGTATCCCAACCAAAAGGGAAGGCTTTACATAAAGAAG

cltr14520M CAAGACCCTGGAAGTATCCCAACCAAAAGGGAAGGCTTTACATAAAGAAG

PI190946M CAAGACCCTGGAAGTATCCCAACCAAAAGGGAAGGCTTTACATAAAGAAG

PI191383M CAAGACCCTGGAAGTATCCCAACCAAAAGGGAAGGCTTTACATAAAGAAG

PI307984M CAAGACCCTGGAAGTATCCCAACCAAAAGGGAAGGCTTTACATAAAGAAG

PI343181M CAAGACCCTGGAAGTATCCCAACCAAAAGGGAAGGCTTTACATAAAGAAG

PI190915M CAAGACCCTGGAAGTATCCCAACCAAAAGGGAAGGCTTTACATAAAGAAG

PI225164M CAAGACCCTGGAAGTATCCCAACCAAAAGGGAAGGCTTTACATAAAGAAG

PI237659M CAAGACCCTGGAAGTATCCCAACCAAAAGGGAAGGCTTTACATAAAGAAG

PI265008M CAAGACCCTGGAAGTATCCCAACCAAAAGGGAAGGCTTTACATAAAGAAG

PI286068M CAAGACCCTGGAAGTATCCCAACCAAAAGGGAAGGCTTTACATAAAGAAG

PI306543M CAAGACCCTGGAAGTATCCCAACCAAAAGGGAAGGCTTTACATAAAGAAG

PI326317M CAAGACCCTGGAAGTATCCCAACCAAAAGGGAAGGCTTTACATAAAGAAG

PI393493S CAAGACCCTGGAAGTATCCCAACCAAAAGGGAAGGCTTTACATAAAGAAG

PI393496M CAAGACCCTGGAAGTATCCCAACCAAAAGGGAAGGCTTTACATAAAGAAG

PI427927M CAAGACCCTGGAAGTATCCCAACCAAAAGGGAAGGCTTTACATAAAGAAG

PI486275 CAAGACCCTGGAAGTATCCCAACCAAAAGGGAAGGCTTTACATAAAGAAG

PI511379 CAAGACCCTGGAAGTATCCCAACCAAAAGGGAAGGCTTTACATAAAGAAG

PI10474M CAAGACCCTGGAAGTATCCCAACCAAAAGGGAAGGCTTTACATAAAGAAG

PI452131 CAAGACCCTGGAAGTATCCCAACCAAAAGGGAAGGCTTTACATAAAGAAG

PI560720M CAAGACCCTGGAAGTATCCCAACCAAAAGGGAAGGCTTTACATAAAGAAG

PI573450S CAAGACCCTGGAAGTATCCCAACCAAAAGGGAAGGCTTTACATAAAGAAG

PI94740M CAAGACCCTGGAAGTATCCCAACCAAAAGGGAAGGCTTTACATAAAGAAG

PI428183U CAAGACCCTGGAAGTATCCCAACCAAAAGGGAAGGCTTTACATAAAGAAG

PI538727U CAAGACCCTGGAAGTATCCCAACCAAAAGGGAAGGCTTTACATAAAGAAG

PI554324 CAAGACCCTGGAAGTATCCCAACCAAAAGGGAAGGCTTTACATAAAGAAG

PI369602S CAAGACCCTGGAAGTATCCCAACCAAAAGGGAAGGCTTTACATAAAGAAG

PI487237S CAAGACCCTGGAAGTATCCCAACCAAAAGGGAAGGCTTTACATAAAGAAG

PI272561M CAAGACCCTGGAAGTATCCCAACCAAAAGGGAAGGCTTTACATAAAGAAG

PI554320 CAAGACCCTGGAAGTATCCCAACCAAAAGGGAAGGCTTTACATAAAGAAG

PI603230 CAAGACCCTGGAAGTATCCCAACCAAAAGGGAAGGCTTTACATAAAGAAG

PI168804M CAAGACCCTGGAAGTATCCCAACCAAAAGGGAAGGCTTTACATAAAGAAG

DQ195068 CAAGACCCTGGAAGTATCCCAACCAAAAGGGAAGGCTTTACATAAAGCAG

AF303376 CAAGACCCTGGAAGTATCCCAACCAAAAGGGAAGGCTTTACATAAAGCAG

DQ022953 CAAGACCCTGGAAGTATCCCAACCAAAAGGGAAGGCTTTACATAAAGCAG

FR719742 CAAGACCCTGGAAGTATCCCAACCAAAAGGGAAGGCTTTACATAAAGAAG

KM388515 CAAGACCCTGGAAGTATCCCAACCAAAAGGGAAGGCTTTACATAAAGAAG

KM388516 CAAGACCCTGGAAGTATCCCAACCAAAAGGGAAGGCTTTACATAAAGAAG

KM388514 CAAGACCCCGGAAGTATCCCAACCAAAAGGGAAGGCTTTACATAAAGAAG

HM746657 CAAGACCCTGGAAGTATCCCAACCAAAAGGGAAGGCTTTACATAAAGAAG

PI486264S CAAGACCCTGGAAGTATCCCAACCAAAAGGGAAGGCTTTACATAAAGAAG

KM388518 CAAGACCCTGGAAGTATCCCAACCAAAAGGGAAGGCTTTACATAAAGAAG

KM388517 CAAGACCCTGGAAGTATCCCAACCAAAAGGGAAGGCTTTACATAAAGAAG

KM388519 CAAGACCCTGGAAGTATCCCAACCAAAAGGGAAGGCTTTACATAAAGAAG

KM388520 CAAGACCCTGGAAGTATCCCAACCAAAAGGGAAGGCTTTACATAAAGAAG

KJ534637 CAAGACCCTGGAAGTATCCCAACCAAAAGGGAAGGCTTTACATAAAGAAG

KM388521 CAAGACCCTGGAAGTATCCCAACCAAAAGGGAAGGCTTTACATAGAGAAG

XM_003569037 CAAGACCGCGGAAGTATTCCAACAGGAGGGCAATGCTTTACACAAAGAAG

HQ647359 CGAACGTAAGTTATGATTACTTCAACGTTGAAGAAGTTGTCGACATGATA

AK376344 CGAACGTAAGTTATGATTACTTCAACGTTGAAGAAGTTGTCGACATGATA

DQ012941 CGAACGTAAGTTATGATTACTTCAACGTTGAAGAAGTTGTCGACATGATA

KJ699390 CGAACGTAAGTTATGATTACTTCAACGTTGAAGAAGTTGTCGACATGATA

AY728807 CAAACGTAAGTTATGATTACTTCAACGTTGAAGAAGTTCTCGACATGATA

JN107537 CGAACGTAAGTTATGATTACTTCAACGTTGAAGAAGTTCTCGACATGATA

JQ693159 CGAACGTAAGTTATGATTACTTCAACGTTGAAGAAGTTCTCGACATGATA

PI428208U CGAACGTAAGTTATGATTACTTCAACGTCGAGGAAGTTCTTGACATGATA

PI487236S CGAACGTAAGTTATGATTACTTCAACGTCGAGGAAGTTCTTGACATGATA

PI662241U CGAACGTAAGTTATGATTACTTCAACGTCGAGGAAGTTCTTGACATGATA

PI428323U CGAACGTAAGTTATGATTACTTCAACGTCGAGGAAGTTCTTGACATGATA

PI538726U CGAACGTAAGTTATGATTACTTCAACGTCGAGGAAGTTCTTGACATGATA

PI428231U CGAACGTAAGTTATGATTACTTCAACGTCGAGGAAGTTCTTGACATGATA

PI554297S CGAACGTAAGTTATGATTACTTCAACGTCGAGGAAGTTCTTGACATGATA

PI355519M CGAACGTAAGTTATGATTACTTCAACGTCGAGGAAGTTCTTGACATGATA

PI487235S CGAACGTAAGTTATGATTACTTCAACGTCGAGGAAGTTCTTGACATGATA

PI554323 CGAACGTAAGTTATGATTACTTCAACGTCGAGGAAGTTCTTGACATGATA

PI508260 CGAACGTAAGTTATGATTACTTCAACGTCGAGGAAGTTCTTGACATGATA

PI487268U CGAACGTAAGTTATGATTACTTCAACGTCGAGGAAGTTCTTGACATGATA

PI662238U CGAACGTAAGTTATGATTACTTCAACGTCGAGGAAGTTCTTGACATGATA

PI369608S CGAACGTAAGTTATGATTACTTCAACGTCGAGGAAGTTCTTGACATGATA

PI486263S CGAACGTAAGTTATGATTACTTCAACGTCGAGGAAGTTCTTGACATGATA

PI538728U CGAACGTAAGTTATGATTACTTCAACGTCGAGGAAGTTCTTGACATGATA

PI428241U CGAACGTAAGTTATGATTACTTCAACGTCGAGGAAGTTCTTGACATGATA

PI428237U CGAACGTAAGTTATGATTACTTCAACGTCGAGGAAGTTCTTGACATGATA

PI219867S CGAACGTAAGTTATGATTACTTCAACGTCGAGGAAGTTCTTGACATGATA

PI170204S CGAACGTAAGTTATGATTACTTCAACGTCGAGGAAGTTCTTGACATGATA

PI418582M CGAACGTAAGTTATGATTACTTCAACGTCGAGGAAGTTCTTGACATGATA

PI428287U CGAACGTAAGTTATGATTACTTCAACGTCGAGGAAGTTCTTGACATGATA

PI603255 CGAACGTAAGTTATGATTACTTCAACGTCGAGGAAGTTCTTGACATGATA

PI452130 CGAACGTAAGTTATGATTACTTCAACGTCGAGGAAGTTCTTGACATGATA

PI428215U CGAACGTAAGTTATGATTACTTCAACGTCGAGGAAGTTCTTGACATGATA

PI428180U CGAACGTAAGTTATGATTACTTCAACGTCGAGGAAGTTCTTGACATGATA

PI662264U CGAACGTAAGTTATGATTACTTCAACGTCGAGGAAGTTCTTGACATGATA

PI220642 CGAACGTAAGTTATGATTACTTCAACGTCGAGGAAGTTCTTGACATGATA

PI317392 CGAACGTAAGTTATGATTACTTCAACGTCGAGGAAGTTCTTGACATGATA

DQ195070 CGAACGTAAGTTATGATTACTTCAACGTCGAGGAAGTTCTTGACATGATA

DQ022952 CGAACGTAAGTTATGATTACTTCAACGTCGAGGAAGTTCTTGACATGATA

PI662242U CGAACGTAAGTTATGATTACTTCAACGTCGAGGAAGTTCTTGACATGATA

cltr17668U CGAACGTAAGTTATGATTACTTCAACGTCGAGGAAGTTCTTGACATGATA

PI662239U CGAACGTAAGTTATGATTACTTCAACGTCGAGGAAGTTCTTGACATGATA

PI487267U CGAACGTAAGTTATGATTACTTCAACGTCGAGGAAGTTCTTGACATGATA

PI352486M CGAACGTAAGTTATGATTACTTCAACGTCGAGGAAGTTCTTGACATGATA

PI277130M CGAACGTAAGTTATGATTACTTCAACGTCGAGGAAGTTCTTGACATGATA

PI362610M CGAACGTAAGTTATGATTACTTCAACGTCGAGGAAGTTCTTGACATGATA

PI573452S CGAACGTAAGTTATGATTACTTCAACGTCGAGGAAGTTCTTGACATGATA

cltr14520M CGAACGTAAGTTATGATTACTTCAACGTCGAGGAAGTTCTTGACATGATA

PI190946M CGAACGTAAGTTATGATTACTTCAACGTCGAGGAAGTTCTTGACATGATA

PI191383M CGAACGTAAGTTATGATTACTTCAACGTCGAGGAAGTTCTTGACATGATA

PI307984M CGAACGTAAGTTATGATTACTTCAACGTCGAGGAAGTTCTTGACATGATA

PI343181M CGAACGTAAGTTATGATTACTTCAACGTCGAGGAAGTTCTTGACATGATA

PI190915M CGAACGTAAGTTATGATTACTTCAACGTCGAGGAAGTTCTTGACATGATA

PI225164M CGAACGTAAGTTATGATTACTTCAACGTCGAGGAAGTTCTTGACATGATA

PI237659M CGAACGTAAGTTATGATTACTTCAACGTCGAGGAAGTTCTTGACATGATA

PI265008M CGAACGTAAGTTATGATTACTTCAACGTCGAGGAAGTTCTTGACATGATA

PI286068M CGAACGTAAGTTATGATTACTTCAACGTCGAGGAAGTTCTTGACATGATA

PI306543M CGAACGTAAGTTATGATTACTTCAACGTCGAGGAAGTTCTTGACATGATA

PI326317M CGAACGTAAGTTATGATTACTTCAACGTCGAGGAAGTTCTTGACATGATA

PI393493S CGAACGTAAGTTATGATTACTTCAACGTCGAGGAAGTTCTTGACATGATA

PI393496M CGAACGTAAGTTATGATTACTTCAACGTCGAGGAAGTTCTTGACATGATA

PI427927M CGAACGTAAGTTATGATTACTTCAACGTCGAGGAAGTTCTTGACATGATA

PI486275 CGAACGTAAGTTATGATTACTTCAACGTCGAGGAAGTTCTTGACATGATA

PI511379 CGAACGTAAGTTATGATTACTTCAACGTCGAGGAAGTTCTTGACATGATA

PI10474M CGAACGTAAGTTATGATTACTTCAACGTCGAGGAAGTTCTTGACATGATA

PI452131 CGAACGTAAGTTATGATTACTTCAACGTCGAGGAAGTTCTTGACATGATA

PI560720M CGAACGTAAGTTATGATTACTTCAACGTCGAGGAAGTTCTTGACATGATA

PI573450S CGAACGTAAGTTATGATTACTTCAACGTCGAGGAAGTTCTTGACATGATA

PI94740M CGAACGTAAGTTATGATTACTTCAACGTCGAGGAAGTTCTTGACATGATA

PI428183U CGAACGTAAGTTATGATTACTTCAACGTCGAGGAAGTTCTTGACATGATA

PI538727U CGAACGTAAGTTATGATTACTTCAACGTCGAGGAAGTTCTTGACATGATA

PI554324 CGAACGTAAGTTATGATTACTTCAACGTCGAGGAAGTTCTTGACATGATA

PI369602S CGAATGTAAGTTATGATTACTTCAACGTCGAGGAAGTTCTTGACATGATA

PI487237S CGAACGTAAGTTATGATTACTTCAACGTCGAGGAAGTTCTTGACATGATA

PI272561M CGAACGTAAGTTATGATTACTTCAACGTCGAGGAAGTTCTTGACATGATA

PI554320 CGAACGTAAGTTATGATTACTTCAACGTCGAGGAAGTTCTTGACATGATA

PI603230 CGAACGTAAGTTATGATTACTTCAACGTCGAGGAAGTTCTTGACATGATA

PI168804M CGAACGTAAGTTATGATTACTTCAACGTCGAGGAAGTTCTTGACATGATA

DQ195068 CGAACGTAAGTTATGATTACTTCAACGTCGAGGAAGTTCTTGACATGATA

AF303376 CGAACGTAAGTTATGATTACTTCAACGTCGAGGAAGTTCTTGACATGATA

DQ022953 CGAACGTAAGTTATGATTACTTCAACGTCGAGGAAGTTCTTGACATGATA

FR719742 CGAACGTAAGTTATGATTACTTCAACGTCGAGGAAGTTCTTGACATGATA

KM388515 CGAACGTAAGTTATAATTACTTCAACGTCGAGGAAGTTCTTGACATGATA

KM388516 CGAACGTAAGTTATAATTACTTCAACGTCGAGGAAGTTCTTGACATGATA

KM388514 CGAACGTAAGTTATAATTACTTCAACGTCGAGGAAGTTCTTGATATGATA

HM746657 CAAACGTAAGTTATGATCACTTCAACGTCGAGGAAGTTCTTGACATGATA

PI486264S CGAATGTAAGTTATGATTACTTCAACGTCGAGGAAGTTCTTGACATGATA

KM388518 CGAACGTAAGTTATGATTACTTCAACGTCGAGGAAGTTCTTGACATGATA

KM388517 CGAACGTAAGTTATGATTACTTCAACGTCGAGGAAGTTCTTGACATGATA

KM388519 CGAACGTAAGTTGTGATTACTTCAACGTCGAGGAAGTTCTTGACATGATA

KM388520 CGAACGTAAGTTATGATTACTTCAACGTCGAGGAAGTTCTTGACATGATA

KJ534637 CGAACGTAAGTTATGATTACTTCAACGTCGAGGAAGTTCTTGACATGATA

KM388521 CGAACGTAAGTTATGATTACTTCAACGTCGAGGAAGTTCTTGACATGATA

XM_003569037 TGAAAGTAAGTTATGATTACTTCAACGTCGAAGAAGTTCTCGACATGATA

HQ647359 ATTGTGGAATTGAGTGCTGATGTAAAAATGGAAGCACATGAAGAGTACCA

AK376344 ATTGTGGAATTGAGTGCTGATGTAAAAATGGAAGCACATGAAGAGTACCA

DQ012941 ATTGTGGAATTGAGTGCTGATGTAAAAATGGAAGCACATGAAGAGTACCA

KJ699390 ATTGTGGAATTGAGTGCTGATGTAAAAATGGAAGCACATGAAGAGTACCA

AY728807 ATTGTGGAATTGAGTGCTGATGTAAAAATGGAAGCACATGAAGAGTACCA

JN107537 ATTGTGGAATTGAGTGCTGATGTAAAAATGGAAGCACATGAAGAGTACCA

JQ693159 ATTGTGGAATTGAGTGCTGATGTAAAAATGGAAGCACATGAAGAGTACCA

PI428208U ATTGTGGAGTTGAGTGCTGATGTAAAAATGGAAGCACATGAAGAGTACCA

PI487236S ATTGTGGAGTTGAGTGCTGATGTAAAAATGGAAGCACATGAAGAGTACCA

PI662241U ATTGTGGAGTTGAGTGCTGATGTAAAAATGGAAGCACATGAAGAGTACCA

PI428323U ATTGTGGAGTTGAGTGCTGATGTAAAAATGGAAGCACATGAAGAGTACCA

PI538726U ATTGTGGAGTTGAGTGCTGATGTAAAAATGGAAGCACATGAAGAGTACCA

PI428231U ATTGTGGAGTTGAGTGCTGATGTAAAAATGGAAGCACATGAAGAGTACCA

PI554297S ATTGTGGAGTTGAGTGCTGATGTAAAAATGGAAGCACATGAAGAGTACCA

PI355519M ATTGTGGAGTTGAGTGCTGATGTAAAAATGGAAGCACATGAAGAGTACCA

PI487235S ATTGTGGAGTTGAGTGCTGATGTAAAAATGGAAGCACATGAAGAGTACCA

PI554323 ATTGTGGAGTTGAGTGCTGATGTAAAAATGGAAGCACATGAAGAGTACCA

PI508260 ATTGTGGAGTTGAGTGCTGATGTAAAAATGGAAGCACATGAAGAGTACCA

PI487268U ATTGTGGAGTTGAGTGCTGATGTAAAAATGGAAGCACATGAAGAGTACCA

PI662238U ATTGTGGAGTTGAGTGCTGATGTAAAAATGGAAGCACATGAAGAGTACCA

PI369608S ATTGTGGAGTTGAGTGCTGATGTAAAAATGGAAGCACATGAAGAGTACCA

PI486263S ATTGTGGAGTTGAGTGCTGATGTAAAAATGGAAGCACATGAAGAGTACCA

PI538728U ATTGTGGAGTTGAGTGCTGATGTAAAAATGGAAGCACATGAAGAGTACCA

PI428241U ATTGTGGAGTTGAGTGCTGATGTAAAAATGGAAGCACATGAAGAGTACCA

PI428237U ATTGTGGAGTTGAGTGCTGATGTAAAAATGGAAGCACATGAAGAGTACCA

PI219867S ATTGTGGAGTTGAGTGCTGATGTAAAAATGGAAGCACATGAAGAGTACCA

PI170204S ATTGTGGAGTTGAGTGCTGATGTAAAAATGGAAGCACATGAAGAGTACCA

PI418582M ATTGTGGAGTTGAGTGCTGATGTAAAAATGGAAGCACATGAAGAGTACCA

PI428287U ATTGTGGAGTTGAGTGCTGATGTAAAAATGGAAGCACATGAAGAGTACCA

PI603255 ATTGTGGAGTTGAGTGCTGATGTAAAAATGGAAGCACATGAAGAGTACCA

PI452130 ATTGTGGAGTTGAGTGCTGATGTAAAAATGGAAGCACATGAAGAGTACCA

PI428215U ATTGTGGAGTTGAGTGCTGATGTAAAAATGGAAGCACATGAAGAGTACCA

PI428180U ATTGTGGAGTTGAGTGCTGATGTAAAAATGGAAGCACATGAAGAGTACCA

PI662264U ATTGTGGAGTTGAGTGCTGATGTAAAAATGGAAGCACATGAAGAGTACCA

PI220642 ATTGTGGAGTTGAGTGCTGATGTAAAAATGGAAGCACATGAAGAGTACCA

PI317392 ATTGTGGAGTTGAGTGCTGATGTAAAAATGGAAGCACATGAAGAGTACCA

DQ195070 ATTGTGGAGTTGAGTGCTGATGTAAAAATGGAAGCACATGAAGAGTACCA

DQ022952 ATTGTGGAGTTGAGTGCTGATGTAAAAATGGAAGCACATGAAGAGTACCA

PI662242U ATTGTGGAGTTGAGTGCTGATGTAAAAATGGAAGCACATGAAGAGTACCA

cltr17668U ATTGTGGAGTTGAGTGCTGATGTAAAAATGGAAGCACATGAAGAGTACCA

PI662239U ATTGTGGAGTTGAGTGCTGATGTAAAAATGGAAGCACATGAAGAGTACCA

PI487267U ATTGTGGAGTTGAGTGCTGATGTAAAAATGGAAGCACATGAAGAGTACCA

PI352486M ATTGTGGAATTGAGTGCTGATGTAAAAATGGAAGCACATGAAGAGTACCA

PI277130M ATTGTGGAATTGAGTGCTGATGTAAAAATGGAAGCACATGAAGAGTACCA

PI362610M ATTGTGGAATTGAGTGCTGATGTAAAAATGGAAGCACATGAAGAGTACCA

PI573452S ATTGTGGAATTGAGTGCTGATGTAAAAATGGAAGCACATGAAGAGTACCA

cltr14520M ATTGTGGAATTGAGTGCTGATGTAAAAATGGAAGCACATGAAGAGTACCA

PI190946M ATTGTGGAATTGAGTGCTGATGTAAAAATGGAAGCACATGAAGAGTACCA

PI191383M ATTGTGGAATTGAGTGCTGATGTAAAAATGGAAGCACATGAAGAGTACCA

PI307984M ATTGTGGAATTGAGTGCTGATGTAAAAATGGAAGCACATGAAGAGTACCA

PI343181M ATTGTGGAATTGAGTGCTGATGTAAAAATGGAAGCACATGAAGAGTACCA

PI190915M ATTGTGGAATTGAGTGCTGATGTAAAAATGGAAGCACATGAAGAGTACCA

PI225164M ATTGTGGAATTGAGTGCTGATGTAAAAATGGAAGCACATGAAGAGTACCA

PI237659M ATTGTGGAATTGAGTGCTGATGTAAAAATGGAAGCACATGAAGAGTACCA

PI265008M ATTGTGGAATTGAGTGCTGATGTAAAAATGGAAGCACATGAAGAGTACCA

PI286068M ATTGTGGAATTGAGTGCTGATGTAAAAATGGAAGCACATGAAGAGTACCA

PI306543M ATTGTGGAATTGAGTGCTGATGTAAAAATGGAAGCACATGAAGAGTACCA

PI326317M ATTGTGGAATTGAGTGCTGATGTAAAAATGGAAGCACATGAAGAGTACCA

PI393493S ATTGTGGAATTGAGTGCTGATGTAAAAATGGAAGCACATGAAGAGTACCA

PI393496M ATTGTGGAATTGAGTGCTGATGTAAAAATGGAAGCACATGAAGAGTACCA

PI427927M ATTGTGGAATTGAGTGCTGATGTAAAAATGGAAGCACATGAAGAGTACCA

PI486275 ATTGTGGAATTGAGTGCTGATGTAAAAATGGAAGCACATGAAGAGTACCA

PI511379 ATTGTGGAATTGAGTGCTGATGTAAAAATGGAAGCACATGAAGAGTACCA

PI10474M ATTGTGGAATTGAGTGCTGATGTAAAAATGGAAGCACATGAAGAGTACCA

PI452131 ATTGTGGAGTTGAGTGCTGATGTAAAAATGGAAGCACATGAAGAGTACCA

PI560720M ATTGTGGAGTTGAGTGCTGATGTAAAAATGGAAGCACATGAAGAGTACCA

PI573450S ATTGTGGAATTGAGTGCTGATGTAAAAATGGAAGCACATGAAGAGTACCA

PI94740M ATTGTGGAATTGAGTGCTGATGTAAAAATGGAAGCACATGAAGAGTACCA

PI428183U ATTGTGGAATTGAGTGCTGATGTAAAAATGGAAGCACATGAAGAGTACCA

PI538727U ATTGTGGAATTGAGTGCTGATGTAAAAATGGAAGCACATGAAGAGTACCA

PI554324 ATTGTGGAATTGAGTGCTGATGTAAAAATGGAAGCACATGAAGAGTACCA

PI369602S ATTGTGGAATTGAGTGCTGATGTAAAAATGGAAGCACATGAAGAGTACCA

PI487237S ATTGTGGAATTGAGTGCTGATGTAAAAATGGAAGCACATGAAGAGTACCA

PI272561M ATTGTGGAATTGAGTGCTGATGTAAAAATGGAAGCACATGAAGAGTACCA

PI554320 ATTGTGGAATTGAGTGCTGATGTAAAAATGGAAGCACATGAAGAGTACCA

PI603230 ATTGTGGAATTGAGTGCTGATGTAAAAATGGAAGCACATGAAGAGTACCA

PI168804M ATTGTGGAATTGAGTGCTGATGTAAAAATGGAAGCACATGAAGAGTACCA

DQ195068 ATTGTGGAATTGAGTGCTGATGTAAAAATGGAAGCACATGAAGAGTACCA

AF303376 ATTGTGGAATTGAGTGCTGATGTAAAAATGGAAGCACATGAAGAGTACCA

DQ022953 ATTGTGGAATTGAGTGCTGATGTAAAAATGGAAGCACATGAAGAGTACCA

FR719742 ATTGTGGAATTGAGTGCTGATGTAAAAACGGAAGCACATGAAGAGTACCA

KM388515 ATTGTGGAATTGAGTGCTGATGTAAAAATGGAAGCACATGAAGAGTACCA

KM388516 ATTGTGGGATTGAGTGCTGATGTAAAAATGGAAGCACATGAAGAGTACCA

KM388514 ATTATGGAATTGAGTGCTGATGTAAAAATGGAAGCACATGAAGAGTACCA

HM746657 ATTGTGGAACTGAGTGCTGATGTAAAAATGGAAGCTCATGAAGAGTACCA

PI486264S ATTGTGGAATTGAGTGCTGATGTAAAAATGGAAGCACATGAAGAGTACCA

KM388518 ATTGTGGAATTGAGTGCTGATGTAAAAATGGAAGCACATGAAGAGTACCA

KM388517 ATTGTGGAATTGAGTGCTGATGTAAAAATGGAAGCACATGAAGAGTACCA

KM388519 ATTGTGGAATTGAGTGCTGATGTAAAAATGGAAGCACATGAAGAGTACCA

KM388520 ATTGTGGAATTGAGTGCTGATGTAAAAATGGAAGCACATGAAGAGTACCA

KJ534637 ATTGTGGAATTGAGTGCTGATGTAAAAATGGAAGCACATGAAGAGTACCA

KM388521 ATTGTGGAATTGAGTGCTGATGTAAAAATGGAAGCACATGAAGAGTACCA

XM_003569037 ATCGTGGAATTGAGTGCTGATAGAAAAATGGAAGTACATGAAGAGTACCA

HQ647359 AGAGGGCGATGACGGGTTTAGTCTTTTCTCATATTAGGGTTCTAGCTATG

AK376344 AGAGGGCGATGACGGGTTTAGTCTTTTCTCATATTAGGGTTCTAGCTATG

DQ012941 AGAGGGCGATGACGGGTTTAGTCTTTTCTCATATTAGGGTTCTAGCTATG

KJ699390 AGAGGGCGATGACGGGTTTAGTCTTTTCTCATATTAGGGTTCTAGCTATG

AY728807 AGAGGGCGATGACGGGTTTAGTCTTTTCTCATATTAGGGTCCTAGCTATG

JN107537 AGAGGGCGATGACGGGTTTAGTCTTTTCTCATATTAG-------------

JQ693159 AGAGGGCAATGACGGGTTTAGTCTTTTCTCATATTAGGGTTCTAGCTATG

PI428208U AGATGGTGATGATGGGTTTAGTCTTTTCTCATATTAGGGTTTTAGCTATG

PI487236S AGATGGTGATGATGGGTTTAGTCTTTTCTCATATTAGGGTTTTAGCTATG

PI662241U AGATGGTGATGATGGGTTTAGTCTTTTCTCATATTAGGGTTTTAGCTATG

PI428323U AGATGGTGATGATGGGTTTAGTCTTTTCTCATATTAGGGTTTTAGCTATG

PI538726U AGATGGTGATGATGGGTTTAGTCTTTTCTCATATTAGGGTTTTAGCTATG

PI428231U AGATGGTGATGATGGGTTTAGTCTTTTCTCATATTAGGGTTTTAGCTATG

PI554297S AGATGGTGATGATGGGTTTAGTCTTTTCTCATATTAGGGTTTTAGCTATG

PI355519M AGATGGTGATGATGGGTTTAGTCTTTTCTCATATTAGGGTTTTAGCTATG

PI487235S AGATGGTGATGATGGGTTTAGTCTTTTCTCATATTAGGGTTTTAGCTATG

PI554323 AGATGGTGATGATGGGTTTAGTCTTTTCTCATATTAGGGTTTTAGCTATG

PI508260 AGATGGTGATGATGGGTTTAGTCTTTTCTCATATTAGGGTTTTAGCTATG

PI487268U AGATGGTGATGATGGGTTTAGTCTTTTCTCATATTAGGGTTTTAGCTATG

PI662238U AGATGGTGATGATGGGTTTAGTCTTTTCTCATATTAGGGTTTTAGCTATG

PI369608S AGATGGTGATGATGGGTTTAGTCTTTTCTCATATTAGGGTTTTAGCTATG

PI486263S AGATGGTGATGATGGGTTTAGTCTTTTCTCATATTAGGGTTTTAGCTATG

PI538728U AGATGGTGATGATGGGTTTAGTCTTTTCTCATATTAGGGTTTTAGCTATG

PI428241U AGATGGTGATGATGGGTTTAGTCTTTTCTCATATTAGGGTTTTAGCTATG

PI428237U AGATGGTGATGATGGGTTTAGTCTTTTCTCATATTAGGGTTTTAGCTATG

PI219867S AGATGGTGATGATGGGTTTAGTCTTTTCTCATATTAGGGTTTTAGCTATG

PI170204S AGATGGTGATGATGGGTTTAGTCTTTTCTCATATTAGGGTTTTAGCTATG

PI418582M AGATGGTGATGATGGGTTTAGTCTTTTCTCATATTAGGGTTTTAGCTATG

PI428287U AGATGGTGATGATGGGTTTAGTCTTTTCTCATATTAGGGTTTTAGCTATG

PI603255 AGATGGTGATGATGGGTTTAGTCTTTTCTCATATTAGGGTTTTAGCTATG

PI452130 AGATGGTGATGATGGGTTTAGTCTTTTCTCATATTAGGGTTTTAGCTATG

PI428215U AGATGGTGATGATGGGTTTAGTCTTTTCTCATATTAGGGTTTTAGCTATG

PI428180U AGATGGTGATGATGGGTTTAGTCTTTTCTCATATTAGGGTTTTAGCTATG

PI662264U AGATGGTGATGATGGGTTTAGTCTTTTCTCATATTAGGGTTTTAGCTATG

PI220642 AGATGGTGATGATGGGTTTAGTCTTTTCTCATATTAGGGTTTTAGCTATG

PI317392 AGATGGTGATGATGGGTTTAGTCTTTTCTCATATTAGGGTTTTAGCTATG

DQ195070 AGATGGTGATGATGGGTTTAGTCTTTTCTCATATTAGGGTTTTAGCTATG

DQ022952 AGATGGTGATGATGGGTTTAGTCTTTTCTCATATTAGGGTTTTAGCTATG

PI662242U AGATGGTGATGATGGGTTTAGTCTTTTCTCATATTAGGGTTTTAGCTATG

cltr17668U AGATGGTGATGATGGGTTTAGTCTTTTCTCATATTAGGGTTTTAGCTATG

PI662239U AGATGGTGATGATGGGTTTAGTCTTTTCTCATATTAGGGTTTTAGCTATG

PI487267U AGATGGTGATGATGGGTTTAGTCTTTTCTCATATTAGGGTTTTAGCTATG

PI352486M AGATGGTGATGATGGGTTTAGTCTTTTCTCATATTAGGGTTTTAGCTATG

PI277130M AGATGGTGATGATGGGTTTAGTCTTTTCTCATATTAGGGTTTTAGCTATG

PI362610M AGATGGTGATGATGGGTTTAGTCTTTTCTCATATTAGGGTTTTAGCTATG

PI573452S AGATGGTGATGATGGGTTTAGTCTTTTCTCATATTAGGGTTTTAGCTATG

cltr14520M AGATGGTGATGATGGGTTTAGTCTTTTCTCATATTAGGGTTTTAGCTATG

PI190946M AGATGGTGATGATGGGTTTAGTCTTTTCTCATATTAGGGTTTTAGCTATG

PI191383M AGATGGTGATGATGGGTTTAGTCTTTTCTCATATTAGGGTTTTAGCTATG

PI307984M AGATGGTGATGATGGGTTTAGTCTTTTCTCATATTAGGGTTTTAGCTATG

PI343181M AGATGGTGATGATGGGTTTAGTCTTTTCTCATATTAGGGTTTTAGCTATG

PI190915M AGATGGTGATGATGGGTTTAGTCTTTTCTCATATTAGGGTTTTAGCTATG

PI225164M AGATGGTGATGATGGGTTTAGTCTTTTCTCATATTAGGGTTTTAGCTATG

PI237659M AGATGGTGATGATGGGTTTAGTCTTTTCTCATATTAGGGTTTTAGCTATG

PI265008M AGATGGTGATGATGGGTTTAGTCTTTTCTCATATTAGGGTTTTAGCTATG

PI286068M AGATGGTGATGATGGGTTTAGTCTTTTCTCATATTAGGGTTTTAGCTATG

PI306543M AGATGGTGATGATGGGTTTAGTCTTTTCTCATATTAGGGTTTTAGCTATG

PI326317M AGATGGTGATGATGGGTTTAGTCTTTTCTCATATTAGGGTTTTAGCTATG

PI393493S AGATGGTGATGATGGGTTTAGTCTTTTCTCATATTAGGGTTTTAGCTATG

PI393496M AGATGGTGATGATGGGTTTAGTCTTTTCTCATATTAGGGTTTTAGCTATG

PI427927M AGATGGTGATGATGGGTTTAGTCTTTTCTCATATTAGGGTTTTAGCTATG

PI486275 AGATGGTGATGATGGGTTTAGTCTTTTCTCATATTAGGGTTTTAGCTATG

PI511379 AGATGGTGATGATGGGTTTAGTCTTTTCTCATATTAGGGTTTTAGCTATG

PI10474M AGATGGTGATGATGGGTTTAGTCTTTTCTCATATTAGGGTTTTAGCTATG

PI452131 AGATGGTGATGATGGGTTTAGTCTTTTCTCATATTAGGGTTTTAGCTATG

PI560720M AGATGGTGATGATGGGTTTAGTCTTTTCTCATATTAGGGTTTTAGCTATG

PI573450S AGATGGTGATGATGGGTTTAGTCTTTTCTCATATTAGGGTTTTAGCTATG

PI94740M AGATGGTGATGATGGGTTTAGTCTTTTCTCATATTAGGGTTTTAGCTATG

PI428183U AGATGGTGATGATGGGTTTAGTCTTTTCTCATATTAGGGTTTTAGCTATG

PI538727U AGATGGTGATGATGGGTTTAGTCTTTTCTCATATTAGGGTTTTAGCTATG

PI554324 AGATGGTGATGATGGGTTTAGTCTTTTCTCATATTAGGGTTTTAGCTATG

PI369602S AGATGGTGATGATGGGTTTAGTCTTTTCTCATATTAGGGTTTTAGCTATG

PI487237S AGATGGTGATGATGGGTTTAGTCTTTTCTCATATTAGGGTTTTAGCTATG

PI272561M AGATGGTGATGATGGGTTTAGTCTTTTCTCATATTAGGGTTTTAGCTATG

PI554320 AGATGGTGATGATGGGTTTAGTCTTTTCTCATATTAGGGTTTTAGCTATG

PI603230 AGATGGTGATGATGGGTTTAGTCTTTTCTCATATTAGGGTTTTAGCTATG

PI168804M AGATGGTGATGATGGGTTTAGTCTTTTCTCATATTAGGGTTTTAGCTATG

DQ195068 AGATGGTGATGATGGGTTTAGTCTTTTCTCATATTAGGGTTTTAGCTATG

AF303376 AGATGGTGATGATGGGTTTAGTCTTTTCTCATATTAGGGTTTTAGCTATG

DQ022953 AGATGGTGATGATGGGTTTAGTCTTTTCTCATATTAGGGTTTTAGCTATG

FR719742 AGATGGTGATGATGGGTTTAGTCTTTTCTCATATTAGGGTTTTAGCTATG

KM388515 AGATGGTGATGATGGGTTTAGTCTGTTCTCATATTAGGGTTTTAGCTATG

KM388516 AGATGGTGATGATGGGTTTAGTCTGTTCTCATATTAGGGTTTTAGCTATG

KM388514 AGATGGTGATGATGGGTTTAGTCTGTTCTCATATTAGGGTTTTAGCTATG

HM746657 AGATGGTGATGATGGGTTTAGTCTTTTCTCATATTAGGGTTTTAGCTATG

PI486264S AGATGGTGATGATGGGTTTAGTCTTTTCTCATATTAGGGTTTTAGCTATG

KM388518 AGATGGTGATGATGGGTTTAGTCTTTTCTCATATTAGGGTTTTAGCTATG

KM388517 AGATGGTGATGATGGGTTTAGTCTTTTCTCATATTAGGGTTTTAGCTATG

KM388519 AGATGGTGACGATGGGTTTAGTCTTTTCTCATATTAGGGTTTTAGCTATG

KM388520 AGATGGTGATGATGGGTTTAGTCTTTTCTCATATTAGGGTTTTAGCTATG

KJ534637 AGATGGTGATGATGGGTTTAGTCTTTTCTCATATTAGGGTTTTAGCTATG

KM388521 AGATGGTGATGATGGGTTTAGTCTTTTCTCATATTAGGGTTTTAGCTATG

XM_003569037 AGATGGCGATGATGGGTTTAGTCTGTTCTCATATTAGGATTTTAGTTATG

HQ647359 AGGGTTGTAGTCATGCGGAGCAATAGGGA

AK376344 AGGGTTGTAGTCATGCGGAGCAATAGGGA

DQ012941 AGGGTTGTAGTCATGCGGAGCAATAGGGA

KJ699390 AGGGTTGTAGTCATGCGGAGCGATAGGGA

AY728807 AGGGTTGTAGTCATGCGGAGCAATAGGGA

JN107537 -----------------------------

JQ693159 AGGGTTGTAGTCATGCGGAGCAATAGGGA

PI428208U AGGGTTGTAGTCATGCGGAGCAATAGGGA

PI487236S AGGGTTGTAGTCATGCGGAGCAATAGGGA

PI662241U AGGGTTGTAGTCATGCGGAGCAATAGGGA

PI428323U AGGGTTGTAGTCATGCGGAGCAATAGGGA

PI538726U AGGGTTGTAGTCATGCGGAGCAATAGGGA

PI428231U AGGGTTGTAGTCATGCGGAGCAATAGGGA

PI554297S AGGGTTGTAGTCATGCGGAGCAATAGGGA

PI355519M AGGGTTGTAGTCATGCGGAGCAATAGGGA

PI487235S AGGGTTGTAGTCATGCGGAGCAATAGGGA

PI554323 AGGGTTGTAGTCATGCGGAGCAATAGGGA

PI508260 AGGGTTGTAGTCATGCGGAGCAATAGGGA

PI487268U AGGGTTGTAGTCATGCGGAGCAATAGGGA

PI662238U AGGGTTGTAGTCATGCGGAGCAATAGGGA

PI369608S AGGGTTGTAGTCATGCGGAGCAATAGGGA

PI486263S AGGGTTGTAGTCATGCGGAGCAATAGGGA

PI538728U AGGGTTGTAGTCATGCGGAGCAATAGGGA

PI428241U AGGGTTGTAGTCATGCGGAGCAATAGGGA

PI428237U AGGGTTGTAGTCATGCGGAGCAATAGGGA

PI219867S AGGGTTGTAGTCATGCGGAGCAATAGGGA

PI170204S AGGGTTGTAGTCATGCGGAGCAATAGGGA

PI418582M AGGGTTGTAGTCATGCGGAGCAATAGGGA

PI428287U AGGGTTGTAGTCATGCGGAGCAATAGGGA

PI603255 AGGGTTGTAGTCATGCGGAGCAATAGGGA

PI452130 AGGGTTGTAGTCATGCGGAGCAATAGGGA

PI428215U AGGGTTGTAGTCATGCGGAGCAATAGGGA

PI428180U AGGGTTGTAGTCATGCGGAGCAATAGGGA

PI662264U AGGGTTGTAGTCATGCGGAGCAATAGGGA

PI220642 AGGGTTGTAGTCATGCGGAGCAATAGGGA

PI317392 AGGGTTGTAGTCATGCGGAGCAATAGGGA

DQ195070 AGGGTTGTAGTCATGCGGAGCAATAGGGA

DQ022952 AGGGTTGTAGTCATGCGGAGCAATAGGGA

PI662242U AGGGTTGTAGTCATGCGGAGCAATAGGGA

cltr17668U AGGGTTGTAGTCATGCGGAGCAATAGGGA

PI662239U AGGGTTGTAGTCATGCGGAGCAATAGGGA

PI487267U AGGGTTGTAGTCATGCGGAGCAATAGGGA

PI352486M AGGGTTGTAGTCATGCGGAGCAATAGGGA

PI277130M AGGGTTGTAGTCATGCGGAGCAATAGGGA

PI362610M AGGGTTGTAGTCATGCGGAGCAATAGGGA

PI573452S AGGGTTGTAGTCATGCGGAGCAATAGGGA

cltr14520M AGGGTTGTAGTCATGCGGAGCAATAGGGA

PI190946M AGGGTTGTAGTCATGCGGAGCAATAGGGA

PI191383M AGGGTTGTAGTCATGCGGAGCAATAGGGA

PI307984M AGGGTTGTAGTCATGCGGAGCAATAGGGA

PI343181M AGGGTTGTAGTCATGCGGAGCAATAGGGA

PI190915M AGGGTTGTAGTCATGCGGAGCAATAGGGA

PI225164M AGGGTTGTAGTCATGCGGAGCAATAGGGA

PI237659M AGGGTTGTAGTCATGCGGAGCAATAGGGA

PI265008M AGGGTTGTAGTCATGCGGAGCAATAGGGA

PI286068M AGGGTTGTAGTCATGCGGAGCAATAGGGA

PI306543M AGGGTTGTAGTCATGCGGAGCAATAGGGA

PI326317M AGGGTTGTAGTCATGCGGAGCAATAGGGA

PI393493S AGGGTTGTAGTCATGCGGAGCAATAGGGA

PI393496M AGGGTTGTAGTCATGCGGAGCAATAGGGA

PI427927M AGGGTTGTAGTCATGCGGAGCAATAGGGA

PI486275 AGGGTTGTAGTCATGCGGAGCAATAGGGA

PI511379 AGGGTTGTAGTCATGCGGAGCAATAGGGA

PI10474M AGGGTTGTAGTCATGCGGAGCAATAGGGA

PI452131 AGGGTTGTAGTCATGCGGAGCAATAGGGA

PI560720M AGGGTTGTAGTCATGCGGAGCAATAGGGA

PI573450S AGGGTTGTAGTCATGCGGAGCAATAGGGA

PI94740M AGGGTTGTAGTCATGCGGAGCAATAGGGA

PI428183U AGGGTTGTAGTCATGCGGAGCAATAGGGA

PI538727U AGGGTTGTAGTCATGCGGAGCAATAGGGA

PI554324 AGGGTTGTAGTCATGCGGAGCAATAGGGA

PI369602S AGGGTTGTAGTCATGCGGAGCAATAGGGA

PI487237S AGGGTTGTAGTCATGCGGAGCAATAGGGA

PI272561M AGGGTTGCAGTCATGCGGAGCAATAGGGA

PI554320 AGGGTTGTAGTCATGCGGAGCAATAGGGA

PI603230 AGGGTTGCAGTCATGCGGAGCAATAGGGA

PI168804M AGGGTTGCAGTCATGCGGAGCAATAGGGA

DQ195068 AGGGTTGCAGTCATGCGGAGCAATAGGGA

AF303376 AGGGTTGCAGTCATGCGGAGCAATAGGGA

DQ022953 AGGGTTGCAGTCATGCGGAGCAATAGGGA

FR719742 AGGGTTGTAGTCATGCGGAGCAATAGGGA

KM388515 AGGGTTGTAGTCATGCGGAGCAATAGGGA

KM388516 AGGGTTGTAGTCATGCGGAGCAATAGGGA

KM388514 AGGGTTGTAGTCATGCGGAGCAATAGGGA

HM746657 AGGGTTGTAGTCATGCGGAGCAATAGGGA

PI486264S AGGGTTGTAGTCATGCGGAGCAATAGGGA

KM388518 AGGGTTGTAGTCATGCGGAGCAATAGGGA

KM388517 AGGGTTGTAGTCATGCGGAGCAATAGGGA

KM388519 AGGGTTGTAGTCATGCGGAGCAATAGGGA

KM388520 AGGGTTGTAGTCATGCGGAGCAATAGGGA

KJ534637 AGGGTTGTAGTCATGCGGAGCAATAGGGA

KM388521 AGGGTTGTAGTCATGCGGAGCAATAGGGA

XM_003569037 AGGGTTGTAGTCATGCGGAGCAATAGGAA
